# A stronger acceptor decreases the rates of charge transfer: ultrafast dynamics and on/off switching of charge separation in organometallic donor–bridge–acceptor systems†

**DOI:** 10.1039/d2sc06409j

**Published:** 2023-09-28

**Authors:** Alexander J. Auty, Paul A. Scattergood, Theo Keane, Tao Cheng, Guanzhi Wu, Heather Carson, James Shipp, Andrew Sadler, Thomas Roseveare, Igor V. Sazanovich, Anthony J. H. M. Meijer, Dimitri Chekulaev, Paul I. P. Elliot, Mike Towrie, Julia A. Weinstein

**Affiliations:** a Department of Chemistry, The University of Sheffield Sheffield S3 7HF UK Julia.Weinstein@Sheffield.ac.uk, a.meijer@sheffield.ac.uk; b Department of Chemical Sciences, University of Huddersfield HD1 3DH UK; c Laser for Science Facility, Rutherford Appleton Laboratory, RCaH, STFC OX11 0QX UK

## Abstract

To unravel the role of driving force and structural changes in directing the photoinduced pathways in donor–bridge–acceptor (DBA) systems, we compared the ultrafast dynamics in novel DBAs which share a phenothiazine (PTZ) electron donor and a Pt(ii) *trans*-acetylide bridge (–C≡C–Pt–C≡C–), but bear different acceptors conjugated into the bridge (naphthalene-diimide, NDI; or naphthalene-monoimide, NAP). The excited state dynamics were elucidated by transient absorption, time-resolved infrared (TRIR, directly following electron density changes on the bridge/acceptor), and broadband fluorescence-upconversion (FLUP, directly following sub-picosecond intersystem crossing) spectroscopies, supported by TDDFT calculations. Direct conjugation of a strong acceptor into the bridge leads to switching of the lowest excited state from the intraligand ^3^IL state to the desired charge-separated ^3^CSS state. We observe two surprising effects of an increased strength of the acceptor in NDI *vs.* NAP: a *ca.* 70-fold slow-down of the ^3^CSS formation—(971 ps)^−1^*vs.* (14 ps)^−1^, and a longer lifetime of the ^3^CSS (5.9 *vs.* 1 ns); these are attributed to differences in the driving force Δ*G*_et_, and to distance dependence. The 100-fold increase in the rate of intersystem crossing—to sub-500 fs—by the stronger acceptor highlights the role of delocalisation across the heavy-atom containing bridge in this process. The close proximity of several excited states allows one to control the yield of ^3^CSS from ∼100% to 0% by solvent polarity. The new DBAs offer a versatile platform for investigating the role of bridge vibrations as a tool to control excited state dynamics.

## Abstract 2

≡ The rate of intersystem crossing increases, whilst the rates of charge separation and recombination decrease in donor–CC–Pt–CC–acceptor systems with a stronger electron acceptor – as revealed by fluorescence upconversion and ultrafast IR methods.

## Graphical abstract

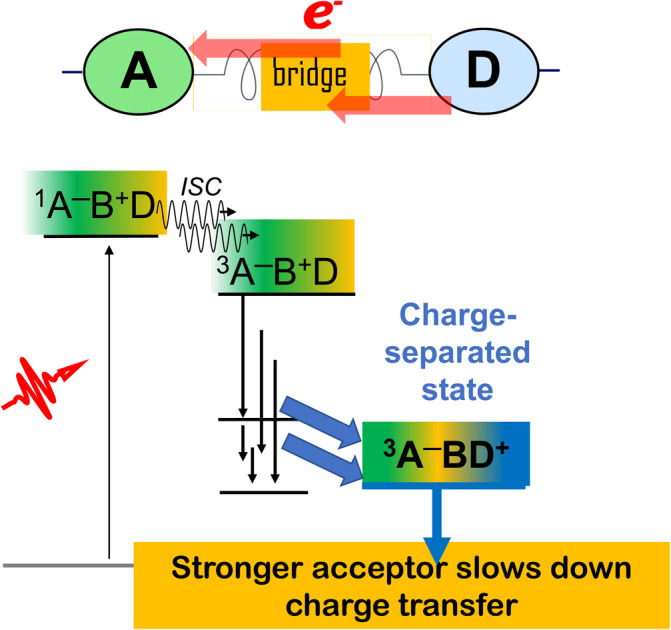

## Introduction

The formation of a charge-separated state (CSS) through photo-induced electron transfer is a key photophysical step in a plethora of light-driven chemical processes, with applications ranging from artificial photosynthesis to photocatalysis.^1–8^ The modular approaches to control charge separation, whereby, for example, separate molecular units are used for catalysis and light absorption, are accordingly of great interest. Such an approach involves a combination of an electron donor, D, an electron acceptor, A, and a bridge, B ‘modules’ assembled in a combinatorial fashion. The synthetic versatility of such modular DBA design, permitting to change the donor and/or acceptor units, bridge length, or the degree of coupling, has allowed for the extensive investigation of how photoinduced electron transfer is affected by structural and electronic properties of the components – the knowledge that can guide future design of efficient light-harvesting systems.

Recent experimental and theoretical studies of ultrafast dynamics in DBA systems have illuminated the crucial role of bridge-localised vibrations in mediating photo-induced electron transfer,^9–13^ with the outcome ranging from full inhibition^10,14^ to acceleration of charge separation.^15^ The Pt(ii) *trans*-acetylide unit [–C≡C–Pt–C≡C–] is often used as a bridge because it ensures directionality of electron transfer,^16–20^ and offers a relative ease of covalent attachment of D and A units.^21–25^ Multiple asymmetric DBA and symmetric (DBD, ABA) systems based on [–C≡C–Pt–C≡C–] bridges have been developed as models for charge transfer process^18,26–31^ relevant to applications including optoelectronics, power limiting, catalysis or upconversion.^32,33^

Recently, a suppression of charge separation with the efficiency of up to 100% has been achieved in organometallic DBA systems, where the two redox-active units were linked by a *trans*-Pt(ii)-acetylide bridge.^10,11,34–36^ The specific family of DBA molecules which exhibited controllable charge-separation contained an aromatic acid imide, 1,8-naphthalimide (NAP) acceptor, and phenothiazine derivatives (PTZ) as donors, in which the coupling between, and the relative energies of, multiple excited states was tuned by varying modes of attachment of the A and the D to the bridge. In “NAP–C≡C–Pt–C≡C–PTZ” DBA, absorption of light populates a charge-transfer (CT) manifold D–B^+^A^−^, which with a rate of (14 ps)^−1^ decays over three pathways, including formation of the CSS (D^+^BA^−^) *via* a reductive quenching of the oxidised bridge by the PTZ donor.^10^

Crucially, branching in such DBAs occurred on a picosecond timescale, commensurate with vibrational cooling, thus allowing one to perturb electron transfer pathway with vibrational excitation. The intriguing and previously unobserved in solution phenomenon was that mode-specific IR-excitation of the bridge-localised acetylide modes in the course of charge separation controlled population of the ^3^CSS, with up to 100% efficiency.^14^ This work, along with other studies,^37–40^ established that the acetylide vibrational modes of the bridge are key contributors to the reaction coordinate in photoinduced electron transfer in these molecules. However, the lowest excited state in the DBA family was an acceptor-based intra-ligand state, ^3^IL, and not the charge-separated state. A logical next step is to design the analogous DBA system, in which the potential for IR-excitation of the bridge is preserved, but the lowest excited state becomes a charge-separated state.

**Fig. 1:**
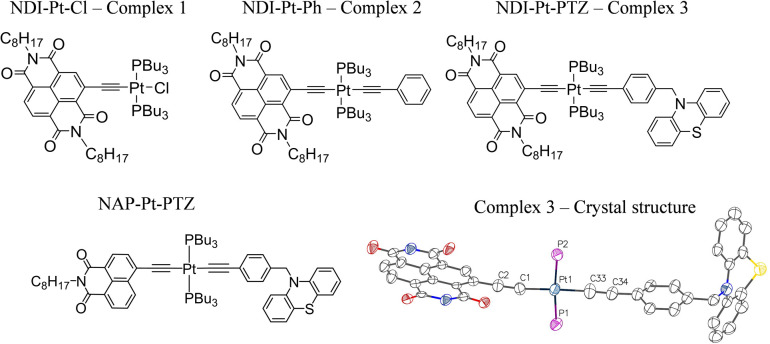
The Pt(ii) DBA complex, 3 and the ‘building block’ complexes 1 and 2. The X-ray crystal structure of 3 is also given, the alkyl chains have been omitted for clarity. Key bond lengths (Å): Pt1–C1 1.96(2), Pt1–C33 1.98(3), Pt1–P1 2.29 (7), Pt1–P2 2.28 (7), C1–C2 1.25 (3), C33–C34 1.25 (3). Key bond angles: C1–Pt1–P1 91.5 (7), P1–Pt1–C33 88.1 (8), C33–Pt1–P2 86.5 (8), C1–Pt1–P2 93.9 (7). CCDC number: 2190247. See ESI† for details. The structure of the 1,8-naphthalimide analog of 3, 3-NAP, is also shown.

Here, the goal of reaching the lowest charge-separated state in *trans*-acetylide DBA systems has been achieved, by replacing the monoimide NAP acceptor with a stronger acceptor, naphthalene diimide NDI (free NDI has *ca.* 0.9 V less negative reduction potential than NAP)^41^ whilst the bridge and the donor remain unchanged, NDI–CC–Pt–CC–PTZ (Fig. 1, 3). Such design reverses the order of the IL and CSS states, potentially enabling a change from a suppression (NAP) to an enhancement of the population of the CSS by exciting bridge vibrations.

**Fig. 2:**
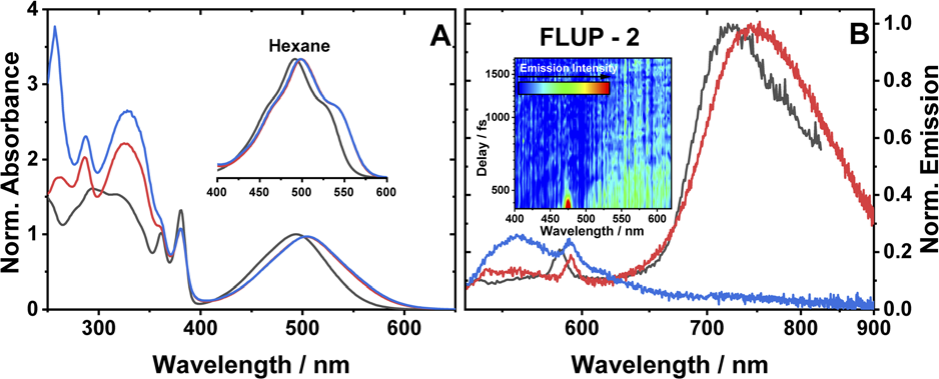
(A) UV-vis absorption spectra of 1 (black), 2 (red) and 3 (blue), in DCM. Spectra are normalised to the peak of the lowest energy absorption band. Inset: a zoomed-in view of the ML-LCT absorption envelope in hexane for 1–3. (B) Emission spectra of 1 (black), 2 (red) and 3 (blue), in aerated DCM at r. t., excitation 504 nm. The small sharp peaks at 585, 590 nm are solvent Raman bands. The emission spectra of 1 and 2 are normalised; the spectrum of 3 is displayed relative to 2 (measured under identical conditions). Global analysis of the data is given in Fig. S3.† (B), inset: a FLUPs 2D-map for 2, excitation 485 nm. FLUPs data for 1 are given in the ESI (Fig. S2).†

We report the unexpected ultrafast photophysics of the novel Pt(ii) DBA complex 3, and of its ‘building blocks’ (Fig. 1, 1 and 2). The chosen donor and acceptor form stable radical cations and anions, respectively,^42–46^ each with distinct spectral features in UV/vis absorption spectra, allowing to track electron transfer in real time. Further, the presence of strong carbonyl group vibrations *ν*(CO) in NDI/NDI^1−^ and of the bridge-localised *ν*(CC) vibrations the frequencies of which are highly sensitive to the changes in electron density, enables the use of time-resolved infrared spectroscopy^47–50^ to follow excited state evolution in the mid-IR region.

A combination of ultrafast time-resolved mid-infrared (TRIR) and transient absorption (TA) methods, flash photolysis, ns–μs time-correlated single photon counting (TCSPC), UV/vis/IR spectroelectrochemistry, broadband fluorescence up-conversion (FLUP) spectroscopy and DFT calculations, allowed us to map the time-resolved dynamics of the new family of Pt(ii) DBA systems over several orders of magnitude in time. We show that the lowest excited state is indeed a desired charge-separated state, and that the relative order of the ^3^IL and ^3^CCS states can also be controlled by solvent polarity. Surprisingly, the DBA containing a stronger electron acceptor and a lower energy ^3^CSS investigated in this work shows several profound differences compared to the previously reported analogues with weaker electron acceptors, namely: a decreased rate of charge separation, a significant increase in the rate of intersystem crossing, and the enhanced lifetime of the ^3^CSS. The role of structural changes across the bridge, the energetics of the individual steps in determining the differences between ultrafast dynamics in the systems bearing acceptors of variable strength, and solvent effects are discussed.

## Synthesis

The acceptor ligand precursor 2-Br-NDI was synthesised *via* a two-step procedure: 1,4,5,8-naphthalene tetra-carboxylic dianhydride was first treated with the brominating agent dibromoisocyanuric acid, followed by formation of the diimide through reaction with *n*-octylamine in refluxing acetic acid.

This synthetic route led to the formation of both 2-Br-NDI and the 2,6-dibrominated species, with the two compounds being efficiently separated by column chromatography.

Whilst Pd-catalysed cross-coupling of 2-Br-NDI and (Me)_3_Si–CCH was successful in introducing an alkynyl group to the 2-position of the naphthalene ring, subsequent deprotection of the (Me)_3_Si– group gave the corresponding free ethynyl species which was found to be unstable under the basic conditions usually employed for the Cu(i)-mediated complexation with Pt(PBu_3_)_2_Cl_2_.^26,40^ Consequently, a synthetic strategy was adopted whereby the ethynyl fragment was complexed with Pt(ii) first, followed by a one-pot deprotection of the alkyne and Pd-catalysed coupling with 2-Br-NDI. Interestingly, whilst this procedure successively furnished the desired ‘acceptor–bridge’ complex NDI–Pt–Cl (1), a second near identical complex was isolated, with mass spectrometry data indicating the formation of an NDI–Pt–Br complex, presumably through halide exchange with free bromide liberated from the 2-Br-NDI ligand-precursor. This latter complex may additionally be identified through a subtle upfield-shift of the ^31^P NMR resonance associated with the coordinated *trans*-phosphine groups (4.66 ppm *vs.* 7.37 ppm for 1 in CDCl_3_). Bis-ethynyl 2 was formed *via* a Cl–Pt–Ph intermediate, itself synthesised through Hagihara coupling of *cis*-Pt(PBu_3_)_2_Cl_2_ with phenylacetylene, with subsequent steps being analogous to those of 1. The triad DBA (3) was formed in an analogous manner. The donor fragment *N*-(4-ethynylbenzyl)-phenothiazine was first complexed with *cis*-Pt(PBu_3_)_2_Cl_2_, followed by a reaction with (Me)_3_Si–CCH to form the trans bis-acetylide (Me)_3_Si–Pt–PTZ. Finally, attachment of the NDI-acceptor was done through *in situ* deprotection and coupling with 2-Br-NDI with a yield of 60%. All characterisation, and synthetic details are given in the ESI.† The X-ray crystal structure of 3 (NDI–Pt–PTZ) is depicted in Fig. 1, with details in the ESI.†

### UV-vis absorption, emission and ultrafast broadband fluorescence upconversion (FLUP) spectroscopy

The ground state absorption spectra of 1–3 in CH_2_Cl_2_ (DCM) are shown in Fig. 2A. The region from 230 to 380 nm contains absorption bands due to ligand-localised transitions (π → π*). The sharp transitions centred at 380 and 360 nm present in all the spectra are assigned to NDI localised π → π* transitions.^35,48^ The transitions centred at 329 and 287 nm are common to 2 and 3 and hence assigned to transitions localised on the phenyl and acetylide fragments. The sharp transition at 257 nm that is only present in the spectrum of 3, is assigned to the PTZ ligand.

The broad transitions at 494 nm (1) and 504 nm (2, 3) are due to a mixed-metal–ligand to ligand charge transfer transition (ML-LCT), involving electron density shift from the Pt-bridge moiety towards the NDI acceptor. The energy of the ML-LCT transitions in 2 and 3 are almost identical, whereas 1, lacking the phenylacetylide fragment, has this transition at slightly higher energy. The broadness of the ML-LCT absorption band suggests that it comprises multiple electronic transitions. (TD)DFT calculations establish the presence of at least three low-lying ML-LCT states. Electron density difference plots (Fig. S15†) between the ground and the first 4 singlet excited states confirm that the transitions involve electron density shift from the whole Pt-acetylide bridge to NDI in 1–3. Calculated UV-vis spectra are in good agreement with the experiment (Fig. S13†).

The emission spectra of 1–3 in aerated DCM, following excitation of the ML-LCT absorption band, are shown in Fig. 2B. All complexes display weak emission in the region 550–650 nm. 1 and 2 also display near-IR emission, with peaks at 725 and 745 nm, which in similar [Pt–CC–NDI]^32,51^ compounds was assigned to emission of ^3^IL states localised on the [–CC–NDI].

Ultrafast broadband fluorescence upconversion was used to spectrally and temporally resolve emission of 1 and 2 in the sub-picosecond domain. The FLUP spectra for 2 (Fig. 2B, inset) shows weak emission in the region 500–620 nm (*λ*_max_ 565 nm) which decays fully within 1 ps. Such ultrafast decay suggests the emission emanates from the initially populated singlet FC state. The rate of intersystem crossing of >(300 fs)^−1^ is similar to that reported recently for some Pt(ii) *trans*-acetylides,^52^ but two orders of magnitude higher than in the NAP–Pt–PTZ analogue.^53^

Complex 3 in DCM does not show the broad near-IR emission from a ^3^IL state observed in 1 and 2, suggesting the presence of a new excited state in 3 involving the PTZ unit: population of this state would offer an additional decay channel for the ^3^IL state, quenching its emission. However, in hexane 2 and 3 show identical ^3^IL emission (Fig. S1†), suggesting that the energy, and therefore the probability of the population of this new excited state, are sensitive to solvent polarity.

### Fourier transform infrared spectroscopy (FTIR)

**Fig. 3:**
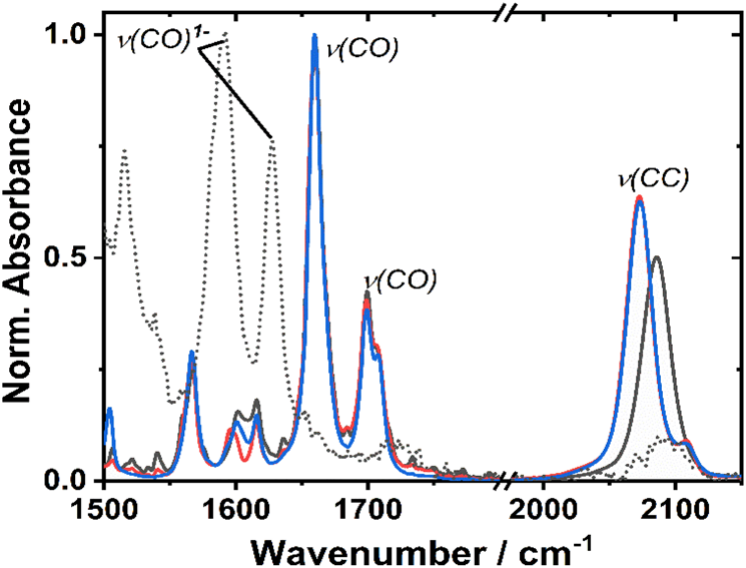
Infrared absorption spectra of 1 (black), 2 (red) and 3 (blue), at r. t. in DCM. The spectrum of the one-electron reduced species of 1, 1^−^, obtained spectro-electrochemically in DCM, applied potential to −1.3 V *vs.* Fc/Fc^+^ is shown as a dashed line.

The ground state FTIR spectra of 1–3 in DCM in the region from 1500 to 2150 cm^−1^ are shown in Fig. 3. 1 has a single *ν*(CC) at 2086 cm^−1^, whilst the two acetylide groups in 2 and 3 yield asymmetric and symmetric group vibrations at 2072 and 2107 cm^−1^, resp. The range 1650–1750 cm^−1^ is dominated by NDI carbonyl stretching vibrations *ν*(CO) at 1659, 1699 cm^−1^, and 1708 cm^−1^ (sh). Multiple bands below 1650 cm^−1^ correspond to aromatic ring stretching modes of the NDI (and Ph/PTZ) groups. The calculated vibrational spectra of the electronic ground state of 1–3 (Fig. S14†) are in good agreement with the experiment.

### Redox properties and (spectro)electrochemistry

Cyclic voltammetry of 1 and 2 in dry DCM (*vs.* Fc/Fc^+^) shows two reversible reduction processes (Fig. S4†) at *E*_1/2_ −1.12 and −1.53 V for 1, and −1.23 and −1.64 V for 2. These potentials are similar to other systems^32,33^ in which the NDI is bound to the Pt *via* direct attachment of the –CC-bond to the aromatic ring, and are assigned to the sequential reduction of the NDI moiety to NDI^1−^ and NDI^2−^ (also confirmed by the spectro-electrochemical data, see below). The reduction potentials are slightly more negative than those of the free NDI, or of the Pt(ii) *trans*-acetylides in which the NDI is linked to the bridge *via* the N-atom.^34^

The changes in the IR-spectrum in the course of the one-electron reduction of 1 (Fig. 3, dashed line) include disappearance of the *ν*(CO) vibrations of the neutral complex and appearance of new vibrations at lower energies at 1515, 1592 and 1628 cm^−1^, due to *ν*(CO) of the NDI radical-anion.^48^ The UV-vis spectrum of the one-electron reduced species, 1^−^ generated electrochemically in dry DCM (Fig. S5†), exhibits new absorption bands at 486, 530 (sh), 585, 625, and 708 nm. The slight difference in these band positions to those of the unsubstituted, symmetric NDI^1−^ (480, 608 nm (ref. 48, 55 and 56)), are consistent with the significant electronic coupling between the NDI ligand and the Pt–CC bridge in 1.

## Ultrafast dynamics of 1–3

The major transient signals for DBA complexes 1–3 in the UV-vis and IR spectral regions are given in Table 1. Kinetic parameters extracted from different methods are given in the ESI, Table S1.†

**Table 1:** Absorption, emission, electrochemical and photophysical properties of 1, 2 and 3 in aerated DCM

Complex	*λ*_max_/nm absorption	*λ*_max_/nm emission	*τ*_em_ b/ns	QY_em_^c^	*E*_1/2 red_ e/V	*E*_p,a_ e/V	TA^f^/nm	TRIR^f^/cm^−1^		Timescale^i^/ps		
								“Carbonyl” region	“Acetylide” region	ISC	CSS_form_	CR
1	494^a^	520 (0.2 ps) → 560 (3 ps) → 725	60 ± 4	0.005	−1.12 (0.07), −1.53 (0.08)	+1.14	429, 629	1608, 1644	1982	0.2, 3 (FLUP TA, TRIR)	n/a	—
2	504^a^ (23 120)	520 (∼0.3 ps), 745	111 ± 10	0.016	−1.23 (0.10), −1.64 (0.10)	+0.88, +1.15	442	1608, 1643	1885/1962/1936	∼0.3 (FLUP, TA, TRIR)	n/a	—
3	504^a^ (11 080)	558	d	0.002	−1.23	+0.34^h^	437/472, 589, 625, 706	1612, 1647/1592, 1626	1885/1963/1928/2097	∼0.26 (TRIR), 0.2 (TA)	970 (TA, TRIR)	5900
1^1−^	486, 530(sh), 585, 625, 708^g^	—		—	—			1515, 1592, 1628^g^				
1-NAP^h^	430	518, (640, 696, 775)^h^	0.014	0.001	−1.83	+0.87				≤200	—	
2-NAP^h^	430	522, (640, 696, 775)^h^	0.020	0.002	−1.83	+1.14				≤20	—	
3-NAP^h^	424	498, (635, 693, 775)^h^	0.209	0.026	−1.83	+0.34, 0.94, 1.13				≤10	≤14	1000

a Absorption maximum of the ML-LCT band. Extinction coefficients (dm^3^ mol^−1^ cm^−1^) in brackets.

b Emission lifetimes, from monoexponential tail fit of the emission decay traces, excitation with a 455 nm pulsed diode.

c Relative to a solution of [Ru(bpy)_3_]Cl_2_ in H_2_O (QY_em_ 0.028,^54^ estimated error 15%).

d Instrument-limited, mini-Tau IRF_(FWHM)_ 160 ps.

e In the presence of 0.2 M *t*Bu_4_NPF_6_; anodic/cathodic peak separation is given in brackets. The CVs are given in Fig. S4.

f Major excited state absorption peaks in TA and TRIR following 500–520 nm excitation. “/” separates bands with different dynamics.

g Spectro-electrochemical data.

h Ref. 35.

i Methods from which timescales have been derived stated. CSS_form_: charge-separated state formation; CR: charge recombination.

### Time-resolved infrared spectroscopy (TRIR)

**Fig. 4:**
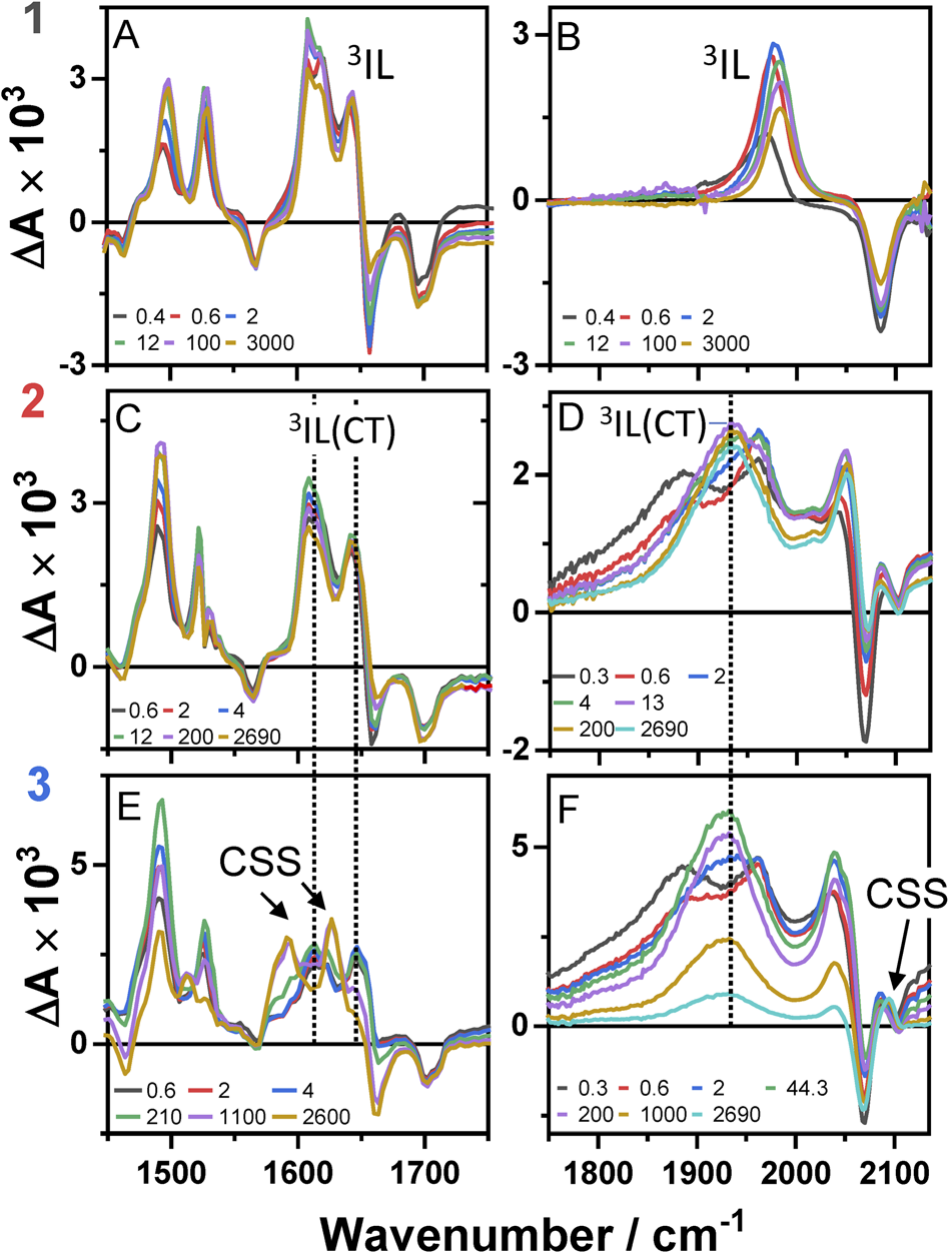
TRIR spectra of 1–3 following excitation of the ML-LCT transition, 500 nm, in DCM, in the range up to 3 ns, at selected time delays as shown. Data for compound 1 (panels A and B), compound 2 (panels C and D), and compound 3 (panels E and F). The data in panels A, C and E correspond to the lower frequency region, 1450–1750 cm^−1^, the data on panels B, D and F – to the higher frequency "acetylide" region, characteristic IR bands attributed to ^3^IL, ^3^IL(CT), and CSS states are labelled.

Time-resolved infrared spectra of 1–3 in DCM, following excitation of the ML-LCT transition are shown in Fig. 4 in the ‘acetylide region’, 1750–2200 cm^−1^ (Fig. 4B, D and F) and in the ‘carbonyl’ region, 1300–1800 cm^−1^ (Fig. 4A, C and E). The complex dynamics were analysed using a combination of single-point kinetic analysis and global lifetime analysis (GLA). The resulting decay associated difference spectra (DAS, parallel model) and evolution associated spectra (EAS, sequential model) are shown in Fig. S6 and S7.†

The TRIR spectra of 1 following excitation into an ML-LCT band (500 nm, Fig. 4A and B) show an instantaneous bleach of the ground state *ν*(ring), *ν*(CO), and *ν*(CC) vibrations at 1567, 1657, 1696, and 2085 cm^−1^, which reach maximum negative absorption values by 400 fs. In the acetylide region, the *ν*(CC) transient initially observed at 1967 cm^−1^ narrows and shifts to 1978 cm^−1^ whilst increasing in intensity, then continues to shift to higher energy (1982 cm^−1^) with a 3.6 ps lifetime – the behaviour typical of vibrational cooling. In the carbonyl region, the two *ν*(CO) transients at 1641 and 1619 cm^−1^ appear after the excitation. The transient at 1641 cm^−1^ shifts to 1644 cm^−1^, with the lifetime 0.4 ps, concomitantly with a small increase in signal strength, likely due to vibrational cooling (usually faster process in organic –CO groups *vs.* a metal-appended –C≡C–).^11^

The transient at 1619 cm^−1^ shifts to 1608 cm^−1^ with the time constant 8.5 ps (the shift to lower energy rules out vibrational cooling as the sole process responsible for the shift). Partial decay of the bands at 1608 and 1982 cm^−1^ is then observed over ≈600 ps, concomitant with the apparent recovery of the *ν*(CO) bleach at 1657 cm^−1^ and the *ν*(CC) bleach at 2085 cm^−1^. However, no such recovery is observed for the bleaches at 1567 and 1695 cm^−1^ over the same timeframe, suggesting that the apparent partial recovery of 1657 and 2085 cm^−1^ is not due to the ground state recovery, but to a new transient due to the population of a new excited state. In both the carbonyl and acetylide regions, the TRIR signals only partially decay on the time-scale of the experiment. The lifetime of the final excited state was determined as 60 ns by flash-photolysis, Fig. S12.†

The TRIR spectra of 2 under excitation at 500 nm (Fig. 4C and D) show instantaneous bleach of the symmetric/asymmetric *ν*(CC) bands at 2069, 2102 cm^−1^, the *ν*(CO) (1658, 1700 cm^−1^) and *ν*(ring) vibrations (1565 cm^−1^). In the acetylide region, formation of the bleach signals is accompanied by the rise of a very broad *ν*(CC) IR-absorption band with a maximum at 1885 cm^−1^, with two very weak signals at 1962, 2038 cm^−1^ pronounced. An offset at the higher energy end of the spectra is due to a tail of the NIR electronic absorption, characteristic of charge-transfer state in Pt *trans*- and *cis*-acetylides.^48–50^ The 1885 cm^−1^ transient almost fully decays by 1 ps (its decay is seen clearer at the lower-energy side, 1800–1850 cm^−1^, where there are no overlapping signals; the single-point decay kinetics in this region yields 0.3 ± 0.1 ps), whilst 1962 and 2038 cm^−1^ bands grow. By 4 ps, whilst the intensity of 2038 cm^−1^ remain the same, a new band starts growing at 1936 cm^−1^ and the electronic offset decreases. By 12 ps, the peak at 1962 cm^−1^ almost disappears. The final *ν*(CC) transients at 1936 and 2038 cm^−1^ persist over the time-scale of the experiment. In the low-frequency region, two *ν*(CO) transients appear at 1642 and 1618 cm^−1^ which over the following 15 ps grow and shift to 1643 and 1608 cm^−1^, resp., and undergo limited evolution thereafter. A combination of single-point kinetic and GLA of the data yields time-constants of 0.3, 3 and 120 ps, and a ‘constant’ >8 ns in the acetylide region; and 0.25, 4, 162 ps, and a ‘constant’ in the carbonyl region. Overall TRIR dynamics of 2 can be described by 0.4 ± 0.2, 3 ± 1, 140 ± 20 ps, and >8 ns (114 ns was obtained by flash photolysis, Fig. S12†).

**Fig. 5:**
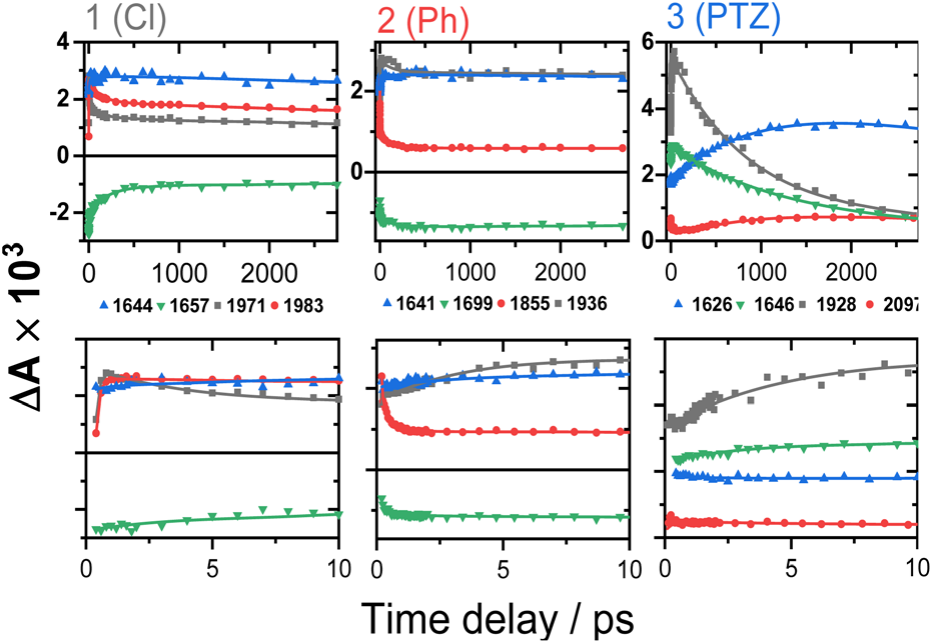
TRIR kinetics of 1–3 up to ∼3 ns (top) and 0–10 ps (bottom), corresponding to the spectral data from Fig. 4. Solid lines: multiexponential fit to the data.

The TRIR spectra of 3 after excitation at 520 nm (Fig. 4E and F) show instant bleaches of the symmetric/asymmetric *ν*(CC) (2069, 2102 cm^−1^), the *ν*(CO) (1663, 1700 cm^−1^) and *ν*(ring) vibrations (1565 cm^−1^). In the acetylide region, an initial, broad *ν*(CC) transient with a maximum at 1885 cm^−1^ decays on sub-picosecond timescales, concomitant with the rise of a new *ν*(CC) band at 1963 cm^−1^. This change is followed by a grow-in of a band at 1928 cm^−1^, similar in spectral shape to the transient at 1936 cm^−1^ observed in 2. These early dynamics occur with the lifetimes 0.26, 4.5 and 126 ps (a minor component, close in shape to the 793 ps component, see Fig. S7F†) similar to those observed in 2. However, unlike 2, for which the *ν*(CC) transient at 1936 cm^−1^ persists beyond 2.7 ns and corresponds to the final excited state, 3 displays significant decay of the main *ν*(CC) transient at 1928 cm^−1^ (Fig. 5) with a 793 ps time-constant, accompanied by the growth of a new transient at 2097 cm^−1^.

**Fig. 6:**
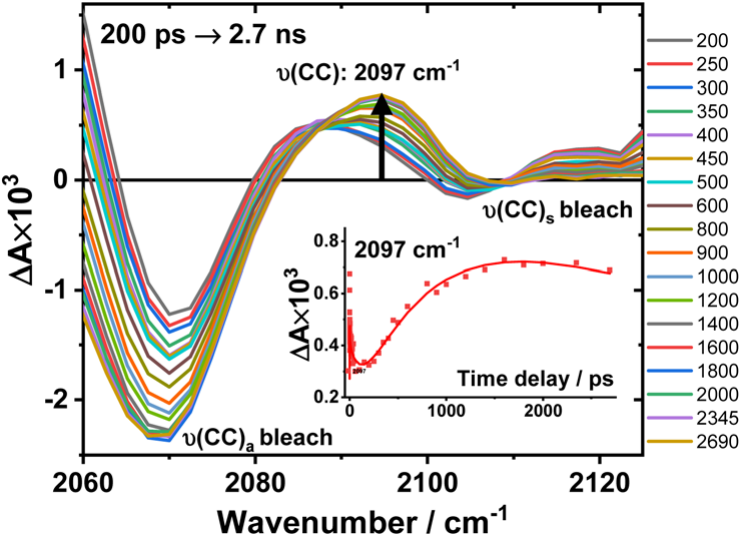
Expanded view of Fig. 4, panel F: the TRIR spectra of 3 in the region 2060–2130 cm^−1^, excitation 520 nm, in DCM. The inset: kinetic trace at 2097 cm^−1^ (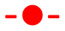), solid line: the best fit obtained from a global analysis of the acetylide region data.

An expanded view of the region 2060–2130 cm^−1^ (Fig. 6) shows the growth of this new 2097 cm^−1^ transient, sandwiched between the bleaches of the asymmetric and symmetric *ν*(CC) bands. This behaviour suggests population of a new excited state with a significant change in electron density on the Pt-bridge. The decay of the 2097 cm^−1^ transient occurs with an estimated lifetime of 5.8 ns (consistent with the TA data below).

In the lower frequency region for 3, immediately following excitation, two *ν*(CO) transients at 1623 (and a 1612 sh) and 1647 cm^−1^ are observed. The spectral evolution involves an increase in signal at 1612 cm^−1^ and on the high-energy side of the 1647 cm^−1^ band, which was best modelled by GLA with time-constants lifetimes 0.33, 3.6, and 61 ps (very minor, and close in shape to 1191 ps component in global analysis, see Fig. S7F†). Significant decay of the *ν*(CO) transients at 1612/1647 cm^−1^ is observed with a lifetime of 1.2 ns (Fig. 4, panel 3), concomitant with the formation of transient bands at 1512, 1592 and 1626 cm^−1^. The final transients in the carbonyl region decay with the same lifetime of 5.8 ns as *ν*(CC) 2097 cm^−1^ band. Thus, 3 populates a new excited state, with characteristic vibrations at and 1512, 1592 and 1626 cm^−1^ (NDI-anion) and 2097 cm^−1^ (Pt/CC) – a charge-separated state.

Overall, TRIR dynamics of 3 (GLA, and lifetime density analysis,^57^ Fig. S10†) can be described by 0.3 ± 0.1 ps, 4 ± 1 ps, 100 ± 40 ps, 1.1 ± 0.2 ns, and 6 ns lifetime of the charge-separated state.

#### Solvent polarity

The dynamics of 3 were highly sensitive to solvent polarity. A change of the solvent from DCM to hexane does not change the TRIR spectra nor dynamics of 2, Fig. S9,† suggesting the lowest excited state is ligand-localised. TRIR spectra of 3 in hexane did not display the *ν*(CC) transients at 1928 and 2097 cm^−1^ which were observed in DCM, indicating no CSS formation in hexane. Emission spectra of 2 and 3 in hexane are identical, and TRIR and TA dynamics of 3 in hexane is similar to that of 2 (Fig. S9†) indicating that the PTZ-donor plays no part in the excited state processes in 3 in non-polar solvents.

### Ultrafast transient absorption spectroscopy (TA)

**Fig. 7:**
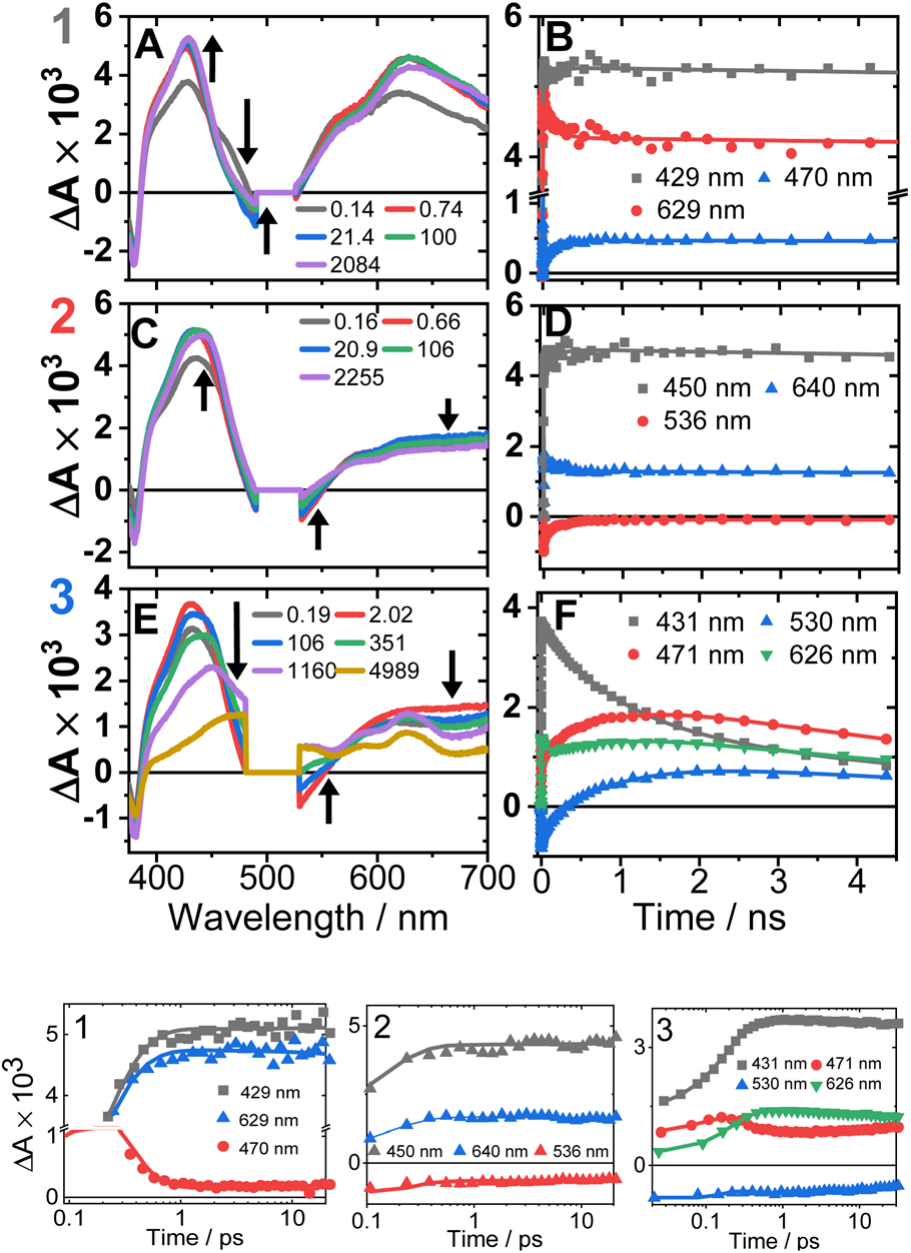
Transient absorption spectra (left panel, time delays stated) and kinetics (right panel, wavelengths stated) following excitation at 500 nm in DCM, for 1 (A and B), 2 (C and D) and 3 (E and F). (Bottom panel) The first 20 ps, the time axis is shown in log scale. The kinetics solid traces are the results of global fit of the data (see Fig. S12† for DAS, EAS).

The transient absorption spectra of 1–3 in DCM in the range 370–700 nm (Fig. 7A, C, E and S11†) at 500 nm excitation show a grow-in of bleach signals (360–380, 480–530 nm), accompanied by the rise of transient signals across the spectral range probed.

TA spectra of 1 display a grow-in of broad transients with peaks at 427 and 629 nm, concomitant with the apparent decay of a shoulder at 465 nm (the downward arrow in Fig. 7A) with *τ* = 0.18 ps. A slight redshift of the band at 427 nm to 429 nm, a small apparent loss in the bleach (475–530 nm), and a decrease in the transient at 629 nm (Fig. 7B) are observed with *τ* = 137 ps. The transient spectrum of 1 then remains unchanged over 5 ns.

Compound 2 displays very similar transient behaviour to that of 1, with a peak at 434 nm and broad featureless absorption in the 570–700 nm region. Similar to 1, there is ultrafast decay of a shoulder to the main transient peak at 434 nm, however the change is too small for GLA to extract a lifetime for this process. The later dynamics of 2 follow a similar pattern to 1: a redshift of the transient to 442 nm, and a decrease in the bleach (483–550 nm) and the transient (570–700 nm), *τ* 142 ps (Fig. 7D).

Compound 3 has initial TA spectrum almost identical to that of 2, with a major peak at 431 nm and featureless transients in the region 570–700 nm. As was the case in 1 and 2, fast decay (0.16 ps) of a small shoulder (470 nm) to the main peak is observed. A shift of the peak from 431 to 437 nm, concomitant with the apparent loss of the bleach (480–550 nm) and decay of transient signal in the 570–700 nm range occur bi-exponentially, with *τ* of 2.1 and 88 ps. The subsequent dynamics involves major decay of the transient signal at 437 nm, along with growth in the region 465–660 nm (Fig. 7F), *τ* 929 ps. The final transient spectrum contains pronounced bands at 589, 625, 706 nm (Fig. S5,† NDI^1−^) and decays with a lifetime of 6.2 ns. The dynamics of the TA data match those of TRIR for 1–3.

## Discussion

1–3 show a broad absorption envelope at around 500 nm, comprising multiple ML-LCT electronic transitions as also evidenced by TDDFT calculations. Excitation populates a manifold of (singlet) excited states with varying degrees of CT character. At early times (<400 fs), neither *ν*(CO) corresponding to NDI^1−^ nor electronic absorption features of NDI^1−^ were observed, suggesting that the first state detected in TRIR and TA has only limited electron density on the acceptor, and/or that the FC-state decays too quickly to be observed: in contrast, in the NAP-analog, NAP^1−^ was detected at early times.^35^

### Ultrafast intersystem crossing

The excited state dynamics of 1–3 at early times are characterised by a growth of *ν*(CO) transients, with very similar frequencies for all complexes (Fig. 4 and Table 1). The growth is accompanied by a continuous shift of the *ν*(CO) transients to higher energy, suggesting that the dynamics reflect a growth in population of an excited state convolved with relaxation within this excited state. This suggests that the observed *ν*(CO) transients belong not to an initial excited state, but rather to one populated by the ultrafast decay of a higher state (likely an initially populated CT state). In similar Pt(ii) NDI systems, *ν*(CO) transients at 1607 and 1647 cm^−1^ were assigned to an excited state localised on the NDI moiety (^3^IL state).^48^ On the other hand, an initial (200 fs) broad transient at 1885 cm^−1^ observed for 2 and 3 is characteristic of the oxidised –CC–Pt–CC moiety, and confirms CT from the Pt-bridge to the acceptor.^34,35^ The *ν*(CC) transient at 1885 cm^−1^ decays on the same timescale as is suggested for the ISC process from the FLUP data, and thus the broad transient at 1885 cm^−1^ is assigned to a singlet CT state, which undergoes ISC (300 fs for 2 and 260 fs for 3) to ^3^MLCT. The 3 ps evolution observed in the carbonyl region of 2 and 3 could be attributed to the decay of ^3^MLCT to a ^3^IL state.

In 1, the 1885 cm^−1^ band (^1^CT state) evolves to a narrow *ν*(CC) transient at 1982 cm^−1^, typical for acceptor-localised excited state and very similar in shape/position of *ν*(CC) in the ^3^NAP.^35^ As noted previously for the NAP analogs, ISC can occur over a range of timescales, and the same is the case in 1. This “continuous” ISC is approximated by a bi-exponential decay of the FLUP signal (0.24 and 3.3 ps), and of TRIR (0.4 and 3.6 ps).

Ultrafast population of a ^3^IL state from a CT-state in 1–3 is also evident in the TA data, by a 200–300 fs decay of a transient at 470 nm and of stimulated emission, accompanied by grow-in at 430, 600–700 nm due to ^3^IL states of the CC-NDI unit.^32,33,51^

### Multiple NDI-based triplet states 2 and 3, and a charge-separated state in 3

Unlike 1, in 2 and 3 the broad *ν*(CC) transient at 1885 cm^−1^ (^1^CT) evolves not into one but into two bands over 20 ps. The *ν*(CC) evolution proceeds as 1885 → 1962 → 1936 cm^−1^, with the lifetimes of 0.3 and 3 ps for 2, and in a very similar manner for 3, 1885 → 1963 → 1928 cm^−1^ (0.26 and 4.5 ps). The formation of two distinct *ν*(CC) reflects a stepwise change in electron density on the bridge over 20 ps, whilst the *ν*(CO) show only limited spectral shift, indicating little change of electron density on NDI. This behaviour suggests the presence of two IL excited states in 2 and 3 with slightly different electron densities on the Pt/CC-bridge. The shift of the *ν*(CC) from 1962/1963 to lower energies, 1936/1928 cm^−1^ indicates an increase in CT-character, hence the state with the *ν*(CC) at 1936/1928 cm^−1^ (2/3) will be termed “^3^IL(CT)”; this state is populated from a ^3^IL state with the time-constant around 140 ps in 2 and 126 ps in 3.

DFT calculations corroborate the existence of an ^3^IL(CT) state in 2 in DCM. The electron density difference plots of 2 (Fig. S15 and 16†), between the lowest energy excited triplet state and the ground state clearly show that whilst the main change in the electron density is on the NDI ligand, some electron density change also occurs on the acetylide units. The calculated IR-spectrum of the lowest triplet state (Fig. S17,† blue trace) is in good agreement with the transient IR spectrum of the final excited state of 2 in DCM (Fig. S17,† black trace), thus the lowest state is assigned as a ^3^IL(CT). The TA spectra of 1 and 2 at 3 ns match those obtained by flash-photolysis (Fig. S12†), with the decay lifetimes of 58 ns (1) and 112 ns (2) that matches the decay of the near-IR emission (60 & 111 ns for 1 & 2, resp.). The lowest excited states in DCM thus is ^3^IL in 1, and ^3^IL(CT) in 2. Importantly, 2 in hexane (Fig. 7, S8 and 9†) shows *ν*(CC) bands at 1885 and 1965 cm^−1^, but not at 1936 cm^−1^ confirming that ^3^IL(CT) state is not formed in non-polar solvents.

In DBA 3, addition of the PTZ-donor results in substantial decay of the transient signals of the ^3^IL(CT) state. The major decay of the *ν*(CC) transient at 1928 cm^−1^ occurs with a lifetime of 793 ps, accompanied by the growth of a *ν*(CC) transient at 2097 cm^−1^. Such large shift in frequency, +169 cm^−1^, suggests a large electron density change on the bridge.^34,35^ In the carbonyl region, the *ν*(CO) transients (1612, 1647 cm^−1^) due to the ^3^IL(CT) state decay with a similar lifetime (1191 ps) to the *ν*(CC) 1928 cm^−1^ transient, along with the formation of new transients at 1592 and 1626 cm^−1^. The new *ν*(CO) bands match the IR spectrum of 1^−^ and are characteristic^48^ of NDI^1−^ (Fig. 3). The new excited state is therefore assigned as the full charge-separated state ^3^CSS, NDI^1−^-Pt-PTZ^1+^. The calculated vibrational spectrum of the ^3^CSS is in excellent agreement with the data (Fig. S17†).

In TA, a decay of TA signal of the ^3^IL(CT) state in 3 with a lifetime of 929 ps is accompanied by the formation of transient bands which match the electronic absorption bands of NDI^1−^. The large change in transient signal is due to formation of the ^3^CSS (Fig. S5,† absorption of PTZ^1+^, 525 nm is obscured by the laser scatter).

The ^3^CSS absorption bands in TA reach their maximum at ∼2 ns, followed by its decay and a concomitant recovery of the bleach at 380 nm with a lifetime of 6.2 ns. The absence of longer-lived states is confirmed by the transient spectrum in 3 decaying fully within the ∼22 ns instrument response of the flash photolysis set-up. Thus, TRIR and TA data, and DFT are consistent in elucidating the lowest excited state as a ^3^CSS in 3 in DCM.

**Fig. 8:**
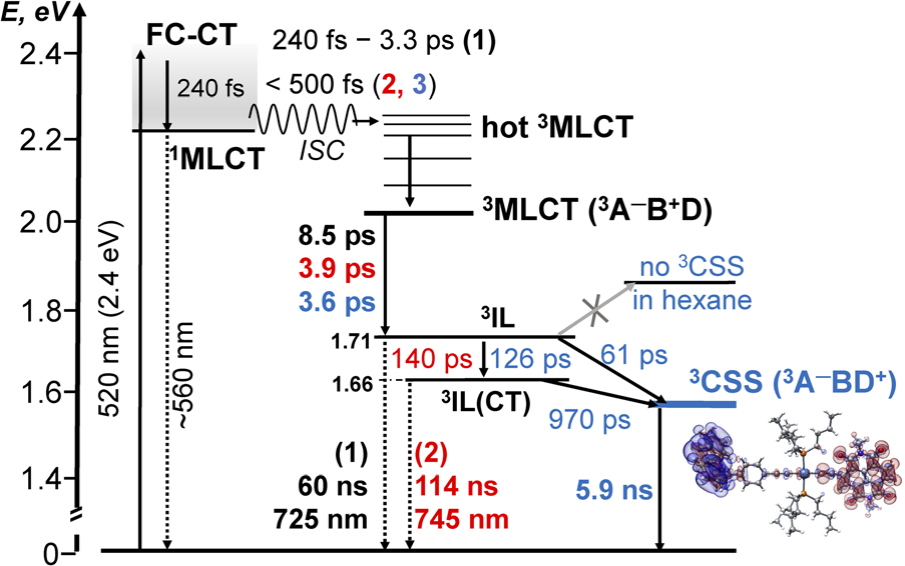
A summary of the excited state dynamics of 1–3, following excitation at 520 nm in DCM. The energy values are estimated from cyclic voltammetry and emission data. Lifetimes for 1 are given in black, for 2 – in 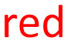 and 3 – in 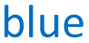. The differential electron density plot for the lowest excited state in 3, ^3^CSS, is also shown. A comparison with the energy level diagram for 3-NAP is given in the ESI (Scheme S1).†

Photophysical processes in 1–3 following ML-LCT excitation are summarised in Fig. 8. The initially populated CT state [evidenced by stimulated emission in TA, the FLUP data, and TRIR transient at 1885 cm^−1^ in 2 &3] decays on ultrafast time-scale, <500 fs into a ^3^MLCT state. In 1, the ^3^MLCT decays into ^3^IL state (1.71 eV, 60 ns), and ^3^IL is the lowest state. In 2 and 3, *two*^3^IL states are populated from the initial CT state, ^3^MLCT → ^3^IL → ^3^IL(CT) as evident from two distinct *ν*(CC) transients. In 2, the lowest excited state is ^3^IL(CT) (1.66 eV) with a lifetime 114 ns. In 3, the ^3^IL(CT) lifetime reduces from 114 ns to *ca.* 970 ps, because of charge-separation *via* electron transfer from the donor, populating ^3^CSS (1.56 eV), ^3^IL(CT) → ^3^CSS (Δ*G*_CS_ = −0.1 eV).

The absence of ground state recovery with (1/970) ps^−1^ rate suggests the ^3^IL(CT) state fully decays into the ^3^CSS, whilst lack of growth of the ^3^CSS with the 0.26 or 4.5 ps lifetimes implies the ^3^CSS is not populated from the higher-lying CT states in 3.

However, this consecutive model cannot explain the difference in the lifetime component attributed to the decay of ^3^IL state in 3: TA (88 ps), acetylide region TRIR (126 ps) and carbonyl region TRIR (61 ps) with large margins; attempts to fit multiple datasets from three different methods with the same (fixed) value of this component did not give satisfactory results. We therefore propose a branching step in the excited state decay in 3: ^3^IL branches into ^3^IL(CT) and ^3^CSS state. The *ν*(CC) are more sensitive to the changes of electron density on the CC-bridge and hence more accurately detect the ^3^IL → ^3^IL(CT) process, approximated with the 126 ps time-constant. The *ν*(CO) transients are more sensitive to the change of electron density on the NDI ligand and also detect ^3^IL → ^3^CSS process (61 ps). To test this suggestion, Lifetime Density Analysis (LDA) was performed for the TRIR data across 1580–2200 cm^−1^ regions. The LDA, which shows a 2D-map of relative amplitudes of decay components at each wavenumber, confirms the presence of both 61 ps, and 126 ps components (Fig. S10,† note the broad distribution of the time constants for the evolution of *ν*(CO), Fig. S10c†) confirming two timescales of population of ^3^CSS. It is not possible to distinguish between the two branching channels in the TA data, the modelling of which yields decay of the ^3^IL state as 88 ps. Comparing the lifetimes extracted from TRIR and TA, we crudely estimate that approximately 40% ^3^IL state decays directly into ^3^CSS, and 60% ^3^IL decays into ^3^IL(CT) first.

Unexpectedly, the lifetime of ^3^CSS in 3 (*E*_CSS_ 1.56 eV) is 6 times higher than that in the NAP–Pt–PTZ (*E*_CSS_ 2.17 eV) despite a ≈0.5 eV larger driving force for charge recombination, Δ*G*_CR_ = −*E*_CSS_.

The Δ*G*_CR_ in both cases is sufficiently negative to place charge recombination in the inverted region (assuming reorganization energy <1.2 eV), yet this would have led to the smaller lifetime of ^3^CSS state in 3 than NAP–Pt–PTZ, contradicting experiments.

**Fig. 9:**
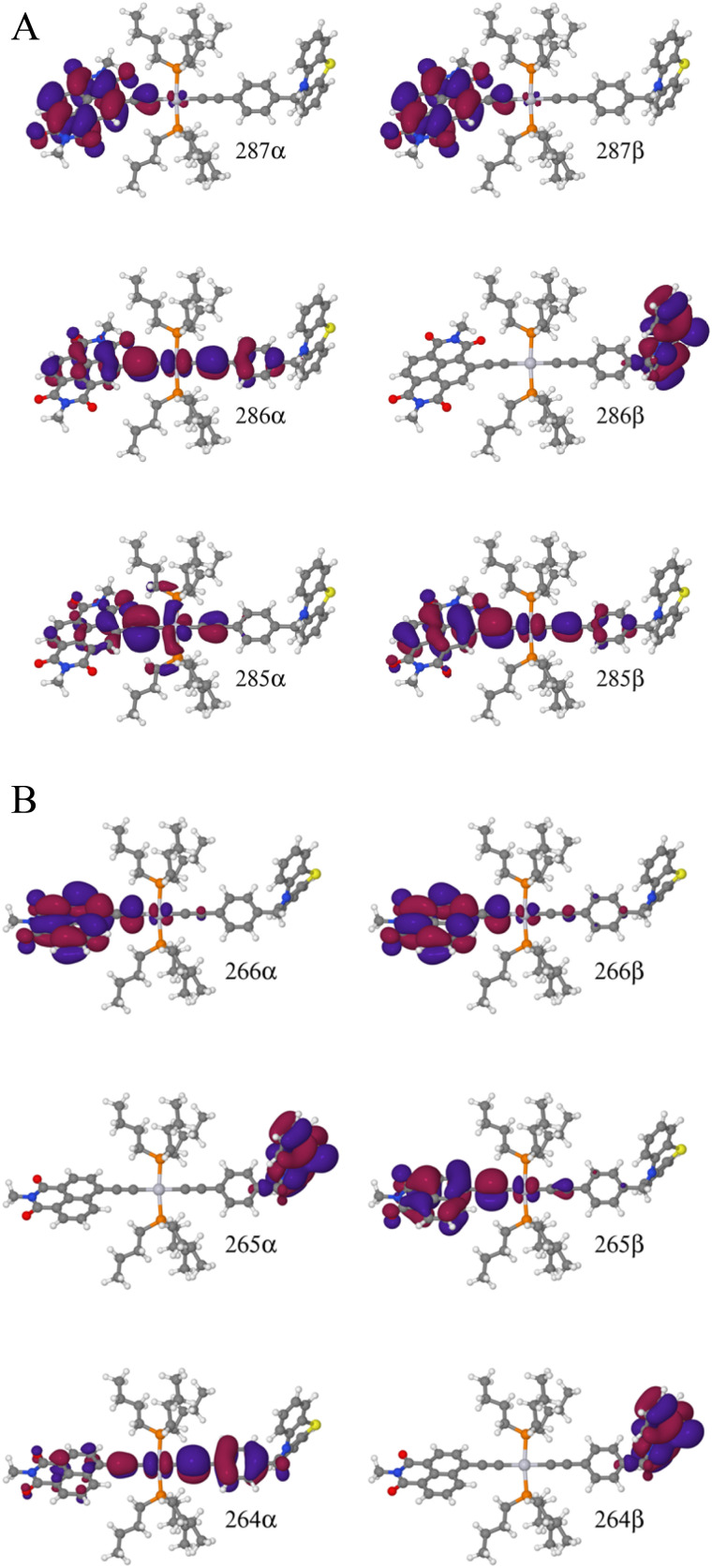
(A) Frontier orbitals for the T_1_ state of 3 for both α and β manifolds. The HOMO for the α manifold is orbital 287α, whereas the HOMO for the β manifold is orbital 285β. (B) Frontier orbitals for the T_1_ state of 3-NAP for both α and β manifolds. The HOMO for the α manifold is orbital 266α, whereas the HOMO for the β manifold is 264β.

To explain this further we considered the electronic structure of the ^3^CSS state in 3 and NAP–Pt–PTZ. The frontier orbitals for these states are given in Fig. 9A and B, respectively.

The T_1_ state for 3 is given as 
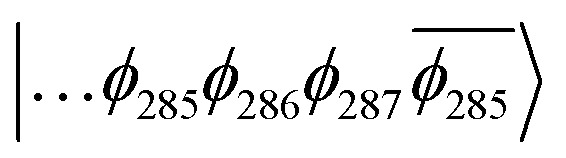
, whereas the T_1_ state for 3-NAP is given as 
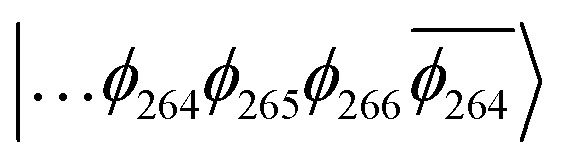
. Correspondingly, the T_2_ state for 3-NAP is achieved by an excitation: 
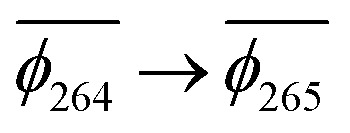
. As a consequence the de-excitation back to the S_0_ ground state is the transition 
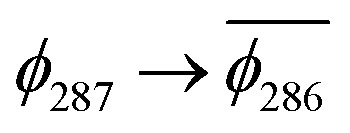
, whereas for 3-NAP it is: 
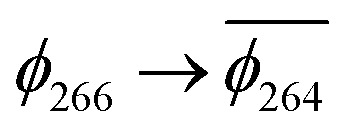
. Inspection of the orbitals involved for both molecules shows that there is little difference between the molecules and very little overlap between the orbitals.

Thus, the likely mechanism of charge recombination in this case is through-space electron tunnelling,^58^ whose rate constant *k*_ET_ decreases exponentially with the donor–acceptor distance *r*:*k*_ET_ = *k*_0_ exp[−*β*(*r* − *r*_0_)]where *k*_0_ is the rate of electron tunnelling at the closest van der Waals donor–acceptor distance *r*_0_, and *β* is a constant. Using previously estimated value *β* = 1.36 Å^−1^ for a *cis*-NDI–Pt–PTZ with *r* = 14.7 Å and *τ* = 36 ns,^50^ and with *r* = 15.5 Å and *τ* = 104 ns, one can predict 6 ns lifetime of the ^3^CSS state in 3, which agrees well with the experiment. This interpretation also agrees with the 130 ns ^3^CSS lifetime in the complex PTZ–Pt–CC–Ph–CH_2_–NDI, where NDI is decoupled from the bridge, Δ*G*_CR_ = −1.44 eV.

An increased lifetime of ^3^CSS in 3 despite its comparatively low energy could be caused by a slightly longer D–A through-space distance of 13.04 Å in 3*vs.* NAP–Pt–PTZ, 12.55 Å and suggests that charge recombination proceeds *via* tunnelling mechanism.

TDDFT calculations identify two triplet manifolds for 3, starting from two distinct and stable T_1_ geometries, which are close in energy (0.43 kJ mol^−1^). The major differences between these two triplet states are the structure of the PTZ moiety, and the relative order of the ^3^IL(CT) and the ^3^CSS states. The small predicted energy difference between these two states explains why their order is readily modulated by solvent polarity, explaining lack of population of ^3^CSS in hexane.

## Conclusions

The intriguing photophysics of new donor–bridge–acceptor Pt(ii) triad bearing a strong NDI acceptor directly conjugated into the –CC–Pt–CC–bridge, 3, and its donor-free precursors 1 and 2, has been resolved by a combination of ultrafast TRIR, transient absorption, fluorescence upconversion spectroscopies, and TDDFT aided by electrochemical studies. We show that such conjugation of the strong acceptor invokes a switch from an intra-ligand to the charge-separated lowest excite state.

Comparison of the photophysical properties of 1–3 with their analogues bearing a weaker acceptor NAP^10,35^ revealed a set of unexpected and counterintuitive observations.

First, the rate of intersystem crossing is orders of magnitude higher in NDI^−^ than in NAP-based DBA, despite a similar energy gap of 0.5 eV between the ^1^CT–^3^IL in 1–3 and the ^1^CT–^3^CT in the NAP-analogues. Whilst a vast variety of ISC rates has been reported for Pt(ii) complexes, the role of many factors (driving force, density of states, vibronic coupling) remains unclear. Here, the increase in the ISC rate could be tentatively ascribed to the singlet manifold being more delocalised across the CC–Pt–CC bridge in the DBA with a stronger acceptor, promoting structural change, and enhancing interstate coupling.

Secondly, the triad DBA 3 and the dyad 2 (–CC–Pt–CC bridge, no donor) possess two intra–CC–NDI states, a “pure” ^3^IL state and a state with a charge-transfer character, ^3^IL(CT). ^3^IL(CT) in 2 has the energy of 1.61 eV and a lifetime of 114 ns, *vs.* 1.9 eV, 190 μs ^3^IL-state in NAP-analogues: the drastic change in the lifetime cannot be explained by the mere 0.3 eV difference in the Δ*G*, pointing to the importance of delocalisation across the bridge.

Further, the rate of charge separation in NAP–Pt–PTZ, with the lowest ^3^IL excited state, and Δ*G*(^3^CT → ^3^CSS) = −0.5–0.3 eV, is ∼70 times higher, 14 ps *vs.* 971 ps ^3^IL(CT) → ^3^CSS charge-separation in 3, probably due to the smaller driving force Δ*G* = −0.1 eV, and a greater through-space D–A distance.

Finally, unexpectedly still, the lifetime of ^3^CSS is 6 times higher in 3 than in NAP–Pt–PTZ despite a ≈0.5 eV smaller driving force for charge recombination, Δ*G*_CR_ = −1.58 *vs.* −2.17 eV, due to distance dependence of electron transfer in the inverted region.

We conclude that in the new DBA *trans*-acetylide triad 3, the introduction of a stronger acceptor directly conjugated into the bridge changes the nature of the lowest excited state to a charge-separated state, ^3^CSS. We observe a surprising slow-down of both charge separation and charge recombination in the DBA systems with a stronger acceptor *vs.* its analogues with the same bridge and donor, but a weaker acceptor. We attribute the first effect to the changes in the driving force for the forward ET, which lies in Marcus kinetic region. The second effect is likely due to distance-dependence of electron tunnelling in the inverted region. The efficiency of charge separation in 3 is strongly modulated by solvent polarity with a full shutdown of charge separation in non-polar solvents. The results provide unexpected insights into the factors governing ultrafast dynamics, whilst the new DBAs offer a versatile basis for investigating the role of bridge vibrations and transient structural change in controlling charge separation.

## Experimental

Synthesis, IR/UV-vis spectroelectrochemistry and cyclic voltammetry are detailed in the ESI.† FTIR spectra were recorded on a PerkinElmer Spectrum One spectrometer, UV-vis spectra on a Cary-50-Bio spectrophotometer, Agilent, and emission spectra on Fluoromax-4 or Duetta fluorimeter, Horiba Scientific.

Transient Absorption (TA) experiments were performed in the Lord Porter Ultrafast Laser Spectroscopy Laboratory, Sheffield, on a Helios spectrometer (HE-VIS-NIR-3200, Ultrafast Systems).

A Ti:Sapp regenerative amplifier (Spitfire ACE PA-40, Spectra-Physics) provided 800 nm pulses (40 fs FWHM, 10 kHz, 1.2 mJ). The amplifier was seeded by 800 nm pulses (25 fs FWHM, 84 MHz) from a Ti:Sapp oscillator (MaiTai, Spectra-Physics). Two amplification stages of the Spitfire ACE were pumped by two Nd:YLF lasers each (Empower). 500/520 nm pump pulses (80 fs FWHM, 2.5 kHz, 0.4 μJ) were generated by a TOPAS prime (Light Conversion), pumped by the 800 nm (40 fs FWHM, 10 kHz, 0.5 mJ) output of the spitfire ACE. The pump pulse passed through a 2.5 kHz mechanical chopper, to allow for the probing of both pumped and un-pumped sample. The pump pulse was depolarised, and focussed onto the sample cell (fused silica, internal pathlength 2 mm), to a spot diameter ≤0.3 mm. A broadband white light probe pulse (340–750 nm) was generated by a portion of the 800 nm output of the spitfire ACE focussed to a 3 mm CaF_2_ crystal. Before generating the white light, the 800 nm pulses were sent to a computer-controlled 8 ns optical delay line (DDS300, Thorlabs, 1.67 fs). The white light was focused on the sample by a protected silver concave mirror (f 50 mm). Kinetic analysis was performed as single points (OriginPro 2018), or Global Lifetime Analysis (GLA).

Ultrafast Broadband Fluorescence Upconversion (FLUP) experiments were performed in the Lord Porter Ultrafast Laser Spectroscopy laboratory, on a set-up developed by Ernsting^59^ and supplied by LIOP-TEC GmbH. 485 nm pump pulses (200 fs FWHM, 10 kHz, 0.5 μJ) were generated as described above for the transient absorption experiments. The pump pulses were sent through a computer-controlled optical delay line (M-IMS400LM, Newport) providing pump-gate delay up to 2.7 ns. Polarization was set to magic angle with respect to vertical axis with a half-wave plate. Pump pulses were focused by a lens (f 200 mm, fused silica) onto the 1 mm quartz sample cell to a spot of *d* < 0.1 mm. The 1320 nm gate pulse (80 fs FWHM, 10 kHz, 60 μJ) were generated by the same laser system as the pump pulse. The polarization of the gate pulse was set to horizontal using a wire-grid polariser and a half-wave plate. Emission from the sample was collected in a forward-scattering geometry, any transmitted pump light blocked by a beam-stopper. The emission was directed onto a 100 μm thick β-barium borate crystal (EKSMA OPTICS) where it was up-converted (sum-frequency) with the 1320 nm gate pulses. The emission and the gate beams met at an angle of ∼21° at the crystal, within the spot *d* = 0.6 mm. Type II phase-matching was used to achieve the broadest spectral window. The upconverted emission signal was spatially filtered and focused by a concave mirror to a fibre bundle (Ceram Optek). A home-built spectrograph dispersed the signal on a CCD (Andor, iDus DU440A-BU2). The 286–500 nm detection range corresponds to the 360–780 nm emission.

Time-Resolved Infrared Spectroscopy (TRIR) experiments were performed on the ULTRA instrument at the STFC Rutherford Appleton Laboratory,^60^ where Ti:Sapp regenerative amplifier (Thales) provided 800 nm pulses (40 fs FWHM, 10 kHz, 1 mJ), used to generate tuneable 400 cm^−1^ broad mid-IR probe pulses. TCSPC luminescence measurements were performed on a mini-τ (Edinburgh Instruments, 445 nm laser diode, 97.5 ps FWHM). Flash Photolysis was done on a home-built Sheffield setup (ESI†).

### Computational details

The majority of calculations were performed with the SMP version of the Gaussian09 package, rev D.01.^61^ DCM was included in all calculations implicitly in the conductor-like polarizable continuum model (C-PCM).^62–65^ Non-standard basis sets were obtained in the basisset exchange.^66–68^ Excited state calculations were performed with linear-response adiabatic time-dependent DFT within the Tamm–Dancoff approximation (LR-TDA-TDDFT).^69^ Benchmark studies^70^ showed that excellent agreement with experimental data was obtained at a reasonable computational cost using the PBE0 functional with the dhf-SVP basis set on Pt^71,72^ and the def2-SVP basis set^73,74^ on all other elements. All geometry optimizations were done using ultrafine integrals, and were followed by frequency calculations. All minima show zero imaginary frequencies.

## Data availability

The data are available in the ESI,† whilst the raw data are available on request from corresponding authors.

## Author contributions

JAW, AJHMM, TK conceived the project; PAS, TC, AS synthesised the compounds; DC, IVS, MT contributed resources & expertise; TK, HC performed calculations; JAW, AJHMM, PIPE supervised others; AJA collected, analysed most data; GW collected, analysed FLUP data; JDS collected, analysed electrochemical data; TR solved X-ray structure; AJA, JAW wrote the paper with input from all authors.

## Conflicts of interest

There are no conflicts to declare.

## Supplementary Material

SC-014-D2SC06409J-s001

SC-014-D2SC06409J-s002
