# Supplementary material for: A stronger acceptor decreases the rates of charge transfer: ultrafast dynamics and on/off switching of charge separation in organometallic donor–bridge–acceptor systems
Source: Chem Sci. 2023 Sep 28;14(41):11417–28. doi: 10.1039/d2sc06409j (PMC10599469; doi:10.1039/d2sc06409j)
Supplement: SC-014-D2SC06409J-s001 [file SC-014-D2SC06409J-s001.pdf]

Electronic Supplementary Material (ESI) for Chemical Science.
This journal is © The Royal Society of Chemistry 2023

A stronger acceptor decreases the rates of charge transfer: Ultrafast dynamics and on/off switching of charge separation in organometallic Donor-Bridge-Acceptor systems

Alexander J. Auty,a Paul A. Scattergood,b Theo Keane,a Tao Cheng,a Guanzhi Wu,a Heather Carson,a James Shipp,a Andrew Sadler,a Thomas Roseveare,a Igor V. Sazanovich,c Anthony J H M Meijer,a* Dimitri Chekulaev,a Paul I. P. Elliot,b Mike Towrie,c Julia A. Weinsteina*

Supporting Information

Synthesis, experimental and theoretical results, additional analysis of spectra, crystallographic data

Contents

1 Synthesis (General methods and synthetic procedures) 2
2 Experimental results 6
2.1 Emission spectroscopy 6
2.2 Time-Resolved Broadband Fluoresence Upconversion (FLUP) of 1 and 2. 7
2.3 Cyclic Voltammetry 9
2.4 UV-vis spectroelectrochemistry and comparison with late-time TA of 3 10
2.5 Time-resolved Infrared Spectroscopy 11
2.6 Transient absorption 18
2.7 Nanosecond Flash Photolysis 19
2.8 Summary of time constants 20
2.9 Scheme S1. Comparison of energy level diagrams and timescales for compound 3, NDI-Pt-PTZ (left side) and its NAP analog, NAP-Pt-PTZ 21
3 Further experimental information 22
4 Theoretical results 23
4.1 Simulated electronic absorption spectra 23
4.2 Simulated vibrational absorption spectra 24
4.3 Density difference plots of the first 4 singlet excited states vs. the ground state in 1-3 25
4.4 Density difference plots on the lowest 4 triplet states relative to the ground state. 26
4.5 Experimental ground state and excited state vibrational spectra overlaid with calculated spectra, and electron density maps 27
5 Additional computational details 28
6 Crystallographic data 96
7 References 107

1 Synthesis (General methods and synthetic procedures)

General methods

Dibromoisocyanuric acid[1], Cl-Pt-PTZ[2], Cl-Pt-Ph[3,4], TMS-Pt-Cl[5] and *cis*-$Pt(PBu_3)_2Cl_2$[2] were prepared following previously reported procedures. All reagents were purchased from Acros Organics, Sigma-Aldrich and Fluorochem and used as received. Synthetic manipulations requiring an inert atmosphere were performed under dry N_2 using standard Schlenk line techniques. Deaeration of solvents was performed through vigorous bubbling with N_2 for a period of at least 15 mins. Dry tetrahydrofuran (THF), triethylamine (Et_3N) and diisopropylamine ($^iPr_2NH$) were obtained from distillation over CaH_2 and stored under an N_2 atmosphere over 4Å molecular sieves. NMR spectra were recorded on Bruker Ascend 400 and 600 MHz spectrometers, with all chemical shifts being reported relative to the residual solvent signal ($CHCl_3$, ^1H δ 7.26, ^{13}C δ 77.16; d_6-acetone, ^1H δ 2.05, ^{13}C δ 29.84, 206.26). High resolution mass spectrometry was performed on an Agilent 6210 time-of-flight instrument with a dual electrospray ionisation source. Matrix Assisted Laser Desorption Ionisation (MALDI) mass spectra were obtained from a Bruker Reflex III instrument using a DCTB matrix. Solid state mass spectra were recorded on a Waters LCT Premier Solids Analysis Probe (ASAP) instrument.

Synthesis of *N,N'-bis*-(*n*-octyl)-2-bromo-1,4,5,8-naphthalene diimide (2-Br-NDI)

1,4,5,8-Naphthalene tetracarboxylic dianhydride (8.66 g, 32.23 mmol) was added to concentrated H_2SO_4 (170 ml) and heated to 85 °C. To the red-brown coloured solution was then added, portionwise over 20 minutes, dibromoisocyanuric acid (9.22 g, 32.1 mmol) and the reaction mixture then heated to 130 °C for a further 21 h. After cooling to r.t., the reaction solution was poured into ice-cold H_2O (300 ml), upon which a bright yellow precipitate was observed. The solids were collected by filtration, washed thoroughly with water followed by a small portion of MeOH and dried *in vacuo*, giving crude 2-bromo-1,4,5,8-naphthalene tetracarboxylic dianhydride as a yellow powder (9.75 g) which was used immediately in the following step without further purification or characterisation.

The previously prepared 2-bromo-1,4,5,8-naphthalene tetracarboxylic dianhydride (9.75 g) deaerated glacial acetic acid (185 ml) and *n*-octylamine (19.0 ml, ρ = 0.78 g/ml, 114.7 mmol) were combined under an N_2 atmosphere and heated to 135 °C for 2 hours. The reaction mixture was then cooled to r.t. and poured into ice-cold H_2O (300 ml). The resultant red-coloured precipitate was collected by filtration, washed thoroughly with H_2O followed by a small portion of MeOH and dried *in vacuo*. The crude solids were re-suspended in 1:1 (v/v) CH_2Cl_2 / petroleum ether (200 ml), stirred for 10 minutes and then collected by filtration. The solids were washed with hexane, dried *in vacuo* and then further purified by column chromatography (SiO_2, 3:1 CH_2Cl_2 / hexane). After removal of pink and yellow coloured bands, the product was collected from the third fraction, which after evaporation of the solvent yielded the title compound as off-white coloured solids. Yield (over two steps) = 2.24 g, 12 %. ^1H NMR ($CDCl_3$, 400 MHz): 0.87 (t, J = 6.3 Hz, 6H), 1.20-1.48 (m, 20 H), 1.67-1.79 (m, 4H), 4.18 (q, J = 7.6 Hz, 4H), 8.76 (d, J = 7.6 Hz, 1H), 8.81 (d, J = 7.6 Hz, 1H), 8.92 (s, 1H). ^{13}C NMR ($CDCl_3$, 101 MHz): 14.23, 22.77, 27.18, 27.28, 28.11, 28.15, 29.31, 29.35, 29.40, 29.42, 31.93, 41.26, 41.64, 124.05, 125.83, 126.12, 126.18, 126.97, 128.74, 128.79, 130.85, 131.80, 138.57, 161.19, 161.86, 161.99, 162.59. HRMS (ES); m/z calc. for $C_{30}H_{38}N_2O_4Br$: 569.2009, found: 569.2005 (MH^+).

The bis-brominated species N,N'-bis-(n-octyl)-2,6-dibromo-1,4,5,8-naphthalene diimide (2,6-Br-NDI) may be obtained by column chromatography from the fraction immediately before the desired product as a yellow powder, discernible by ^1H NMR spectroscopy by a singlet resonance at 8.99 ppm ($CDCl_3$) corresponding to the two equivalent naphthalene protons. It is noted that the four equivalent naphthalene protons in un-substituted N,N'-bis-(n-octyl)-1,4,5,8-naphthalene diimide give a singlet resonance in ^1H NMR spectra at 8.75 ppm ($CDCl_3$).

TMS-Pt-Cl

Following an adapted literature procedure[5], *cis*-Pt(PBu_3)$_2$$Cl_2$ (438 mg, 0.65 mmol) and CuI (11 mg, 0.057 mmol, 8 mol%) were added to deaerated Et_2NH (30 ml) under an N_2 atmosphere. Trimethylsilylacetylene (83 µl, ρ = 0.7 g/ml, 0.59 mmol) was added and the solution stirred at 45 °C for 8 h. The solvent was removed by rotary evaporation and the crude residue purified *via* column chromatography (SiO_2, 7:3 hexane / CH_2Cl_2), giving the product as a colourless oil. Yield = 312 mg, 72%. ^{1}H NMR ($CDCl_3$, 400 MHz): 0.05 (s, 9H), 0.92 (t, J = 7.1 Hz, 18H), 1.37-1.58 (m, 24H), 1.89-2.04 (m, 12H). ^{31}P NMR ($CDCl_3$, 162 MHz): 6.14 (J_{Pt-P} = 2394 Hz). ^{13}C NMR ($CDCl_3$, 101 MHz): 1.22, 13.93, 21.91 (t, J = 17.0 Hz), 24.38 (t, J = 6.7 Hz), 26.14, 102.46 (t, J = 14.2 Hz), 104.72. MALDI-TOF-MS: 696.4 (M-Cl)$^+$.

Synthesis of NDI-Pt-Cl (1)

TMS-Pt-Cl (229 mg, 0.31 mmol), 2-Br-NDI (178 mg, 0.31 mmol), Pd(PPh_3)$_2$$Cl_2$ (33 mg, 0.047 mmol, 15mol%) and CuI (14 mg, 0.073 mmol, 24 mol%) were added to a two-neck flask and placed under a N_2 atmosphere. Thoroughly deaerated and dry 20:1 (v/v) THF / Et_3N (20 ml) was added and the reaction solution stirred under N_2 at r.t for 10 mins. Tetrabutyl ammonium fluoride solution (TBAF, 1M in THF) (0.31 ml, 0.31 mmol) was added dropwise over 2-3 mins and the reaction solution stirred at r.t. for 24 hours, before evaporation of the solvent under reduced pressure. The crude residue was purified by column chromatography (SiO_2 3:1 CH_2Cl_2 / hexane), discarding weak pink-, orange- and yellow-coloured bands with collection of the dark-red fraction. Evaporation of the solvent yielded a dark-red residue (209 mg) which was found to contain both 2-NDI-Pt-Cl and 2-NDI-Pt-Br. These complexes could be separated by further column chromatography (SiO_2, toluene), with the desired chloride-containing complex eluting from the second of two closely spaced red-coloured bands. Yield = 40 mg, 11%. ^{1}H NMR ($CDCl_3$, 400 MHz): 0.84-0.95 (m, 24), 1.22-1.50 (m, 32H), 1.57-1.66 (m, 12H), 1.66-1.76 (m, 4H), 2.03-2.18 (m, 12H), 4.11-4.20 (m, 4H), 8.55 (s, 1H), 8.59 (d, J = 7.6 Hz, 1H), 8.70 (d, J = 7.6 Hz, 1H). ^{31}P NMR ($CDCl_3$, 162 MHz): 7.37 (J_{Pt-P} = 2316 Hz). ASAP-TOF MS: 1147.8.

Whilst the ^{1}H NMR spectra of 2-NDI-Pt-Cl and 2-NDI-Pt-Br are near identical, the latter may be identified by ^{31}P NMR ($CDCl_3$, 162 MHz): 4.66 (J_{Pt-P} = 2295 Hz), ASAP-TOF MS: 1191.7.

TMS-Pt-Ph

Cl-Pt-Ph (136 mg, 0.18 mmol) and CuI (4 mg, 0.021 mmol, 11mol%) were added to deaerated, dry 2:1 (v/v) ^{i}Pr$_2$NH / THF (20 ml) under an N_2 atmosphere. Trimethylsilylacetylene (0.07 ml, ρ = 0.7 g/ml, 0.49 mmol) was added and the solution stirred for 4 h at r.t. The solvent was removed by rotary evaporation and the crude residue purified by column chromatography (SiO_2, 2% EtOAc / hexane), giving a very pale-yellow coloured oil. Yield = 121 mg, 82 %. ^{1}H NMR ($CDCl_3$, 400 MHz): 0.07 (s, 9H), 0.92 (t, J = 7.3 Hz, 18H), 1.37-1.49 (m, 12H), 1.51-1.63 (m, 12H), 2.00-2.17 (m, 12H), 7.08 (t, J = 7.2 Hz, 1H), 7.17 (t, J = 7.5 Hz, 2H), 7.24 (d, J = 7.5 Hz, 2H). ^{13}C NMR ($CDCl_3$, 101 MHz): 1.34, 13.97, 23.86 (t, J = 17.0 Hz), 24.50 (t, J = 6.7 Hz), 26.43, 108.68 (t, J = 14.8 Hz), 109.13, 112.55, 124.85, 127.92, 129.19, 130.92. ^{31}P NMR ($CDCl_3$, 162 MHz): 2.24 (J_{Pt-P} = 2373 Hz). MALDI-TOF-MS: 797.4 (M^+).

Synthesis of NDI-Pt-Ph (2)

TMS-Pt-Ph (120 mg, 0.15 mmol), 2-Br-NDI (85 mg, 0.15 mmol), Pd(PPh_3)$_2$$Cl_2$ (15 mg, 0.021 mmol, 14 mol%) and CuI (7 mg, 0.036 mmol, 25 mol%) were added to a two-neck flask and placed under a N_2 atmosphere. Thoroughly deaerated and dry 10:1 (v/v) THF / Et_3N (20 ml) was added and the mixture stirred under N_2 at r.t. for 30 mins. TBAF (1M in THF) (0.165 ml, 0.165 mmol) was added dropwise over 2-3 mins and the resultant dark red-brown coloured solution stirred at r.t. for 20 hours. The solution was passed through a short (1 cm) SiO_2 pad and the filtrate evaporated to dryness. The crude residue was purified *via* column chromatography (SiO_2, 1:1 CH_2Cl_2 / hexane), with the product eluting from

the deep-red coloured band. Yield = 96 mg, 53 %. ^{1}H NMR (d_6-acetone, 400 MHz): 0.84-0.90 (m, 6H), 0.94 (t, J = 7.3 Hz, 18H), 1.25-1.57 (m, 32H), 1.67-1.80 (m, 16H), 2.25-2.40 (m, 12H), 4.08-4.18 (m, 4H), 7.12 (tt, J = 1.4, 7.2 Hz, 1H), 7.20-7.31 (m, 4H), 8.49 (s, 1H), 8.51 (d, J = 7.6 Hz, 1H), 8.62 (d, J = 7.6 Hz, 1H). ^{31}P NMR (d_6-Acetone, 162 MHz): 4.17 (J_{Pt-P} = 2329 Hz). ^{13}C NMR (d_6-acetone, 151 MHz): 14.19, 14.33, 14.36, 23.29, 23.31, 24.73 (t, J = 17.7 Hz), 25.04 (t, J = 6.8 Hz), 27.28, 27.74, 27.97, 28.64, 28.81, 29.92, 30.02, 30.18, 32.54, 32.60, 41.10, 107.99 (t, J = 15.1 Hz), 110.95, 114.46, 123.78, 125.03, 125.67, 125.83, 126.55, 127.45, 128.80, 128.83, 129.33, 130.06, 131.16, 131.45, 133.74, 138.54, 139.22, 139.32, 139.41, 162.49, 163.31, 163.57, 163.78. MALDI-TOF-MS: 1214.6 $(M+H)^+$.

Synthesis of TMS-Pt-PTZ

Cl-Pt-PTZ (375 mg, 0.39 mmol) and CuI (6 mg, 0.03 mmol) were combined in deaerated, dry 3:1 (v/v) ^{i}Pr$_2$NH / THF (20 ml) under an N_2 atmosphere. Trimethylsilylacetylene (0.09 ml, ρ = 0.7 g/ml, 0.64 mmol) was added and the mixture stirred at r.t. for a further 5 h. Removal of the solvent by rotary evaporation afforded a pale-yellow coloured residue which was purified by column chromatography (SiO_2, 3% EtOAc / hexane), giving the pure product as a very pale yellow solid. Yield = 346 mg, 87 %. ^{1}H NMR ($CDCl_3$, 400 MHz): 0.07 (s, 9H), 0.91 (t, J = 7.0 Hz, 18Hz), 1.37-1.48 (m, 12H), 1.50-1.62 (m, 12H), 2.00-2.17 (m, 12H), 5.01 (s, 2H), 6.63 (d, J = 7.8 Hz, 2H), 6.84 (t, J = 7.5 Hz, 2H), 6.95 (t, J = 7.5 Hz, 2H), 7.06 (d, J = 7.5 Hz, 2H), 7.10 (d, J = 7.8 Hz, 2H), 7.20 (d, J = 7.8 Hz, 2H). ^{13}C NMR ($CDCl_3$, 151 MHz): 1.33, 13.97, 23.83 (t, J = 17.0 Hz), 24.49 (t, J = 6.9 Hz), 26.41, 52.87, 108.71, 109.07 (t, J = 14.5 Hz), 112.54, 115.66, 122.52, 123.09, 126.28, 126.84, 127.32, 131.23, 132.99, 144.63. ^{31}P NMR ($CDCl_3$, 162 MHz): 2.21 (J_{Pt-P} = 2371 Hz). HRMS (ES); m/z calc. for $C_{50}H_{77}NP_2SSiPt$: 1008.4669, found: 1008.4709 (M^+).

Synthesis of NDI-Pt-PTZ (3)

TMS-Pt-PTZ (202 mg, 0.20 mmol), 2-Br-NDI (119 mg, 0.21 mmol), $Pd(PPh_3)_2Cl_2$ (20 mg, 0.028 mmol, 14 mol%) and CuI (12 mg, 0.063 mmol, 31 mol%) were added to a 2-neck flask and placed under an N_2 atmosphere. Thoroughly deaerated and dry 10:1 (v/v) THF / Et_3N (20 ml) was added and the resultant mixture stirred for 35 mins at r.t. TBAF (1M in THF) (0.22 ml, 0.22 mmol) was added dropwise over a period of 5 mins and the red-brown coloured solution stirred for a further 22 h at r.t. The reaction solution was passed through a short (1cm) SiO_2 pad and the filtrate evaporated to leave a dark-red coloured residue which was purified *via* column chromatography (SiO_2, 1:1 CH_2Cl_2 / hexane). After removal of pale yellow, orange and pink coloured bands, the product was obtained from the deep-red coloured fraction. Yield = 172 mg, 60 %. ^{1}H NMR ($CDCl_3$, 400 MHz): 0.84-0.93 (m, 24H), 1.21-1.50 (m, 32H), 1.58-1.77 (m, 16H), 2.13-2.29 (m, 12H), 4.11-4.20 (m, 4H), 5.03 (s, 2H), 6.64 (dd, J = 0.8, 8.0 Hz, 2H), 6.85 (td, J = 1.0, 7.5 Hz, 2H), 6.96 (td, J = 1.6, 7.6 Hz, 2H), 7.07 (dd, J = 1.5 Hz, 2H), 7.15 (d, J = 8.2 Hz, 2H), 7.24 (d, J = 8.2 Hz, 2H), 8.57 (d, J = 7.7 Hz, 1H), 8.58 (s, 1H), 8.69 (d, J = 7.7 Hz, 1H). ^{1}H NMR (d_6-Acetone, 400 MHz): 0.83-0.96 (m, 24H), 1.24-1.56 (m, 32H), 1.66-1.79 (m, 16H), 2.23-2.38 (m, 12H), 4.08-4.19 (m, 4H), 5.13 (s, 2H), 6.79 (dd, J = 0.9, 8.2 Hz, 2H), 6.89 (td, J = 1.0, 7.5 Hz, 2H), 7.05 (td, J = 1.5, 7.7 Hz, 2H), 7.10 (dd, J = 1.5, 7.5 Hz, 2H), 7.23 (d, J = 9.5 Hz, 2H), 7.26 (d, J = 9.0 Hz, 2H), 8.50 (s, 1H), 8.53 (d, J = 7.7 Hz, 1H), 8.64 (d, J = 7.7 Hz, 1H). ^{13}C NMR (d_6-Acetone, 101 MHz): 14.25, 14.36, 14.39, 23.31, 23.33, 24.71 (t, J = 17.5 Hz), 25.06 (t, J = 6.7 Hz), 27.29, 27.79, 28.01, 28.66, 28.81, 32.56, 32.63, 41.12, 52.58, 108.11, 108.23, 110.75, 114.49, 116.69, 123.40, 123.70, 124.10, 124.84, 125.44, 126.39, 127.22, 127.42, 127.53, 128.17, 128.68, 128.76, 129.16, 131.03, 131.69, 133.75, 134.60, 138.57, 145.56, 162.37, 163.16, 163.41, 163.58. ^{31}P NMR ($CDCl_3$, 162 MHz): 3.39 (J_{Pt-P} = 2317 Hz). ^{31}P NMR (d_6-Acetone, 162 MHz): 4.10 (J_{Pt-P} = 2333 Hz). MALDI-TOF-MS: 1424.7 (M^+), 1225.7 $(M-PBu_3)^+$.

Synthetic scheme

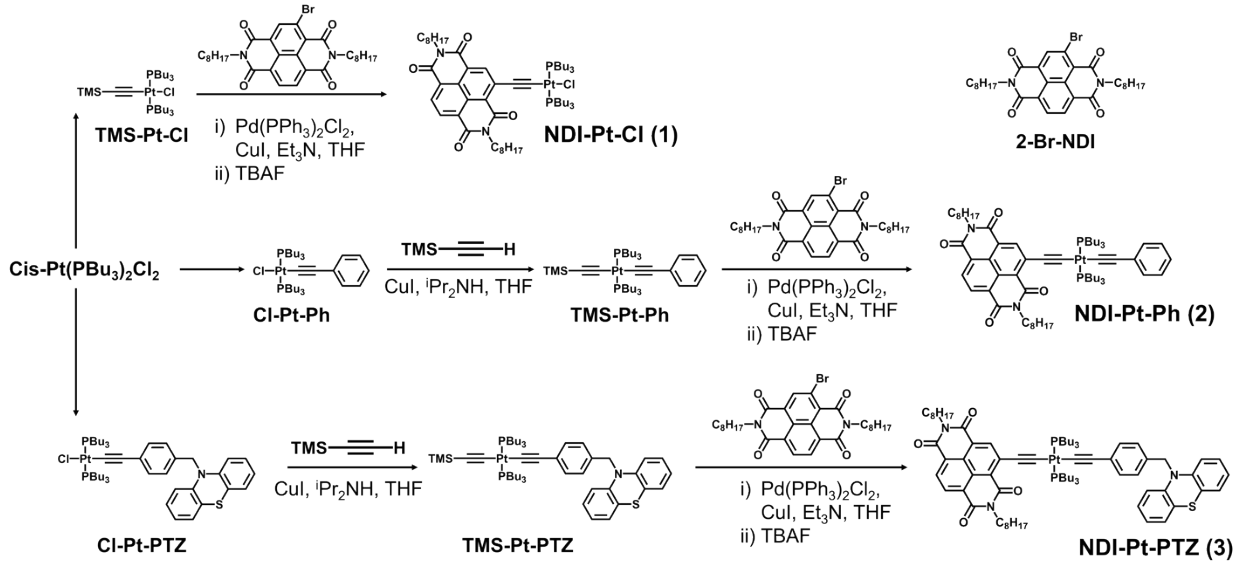

2 Experimental results

2.1 Emission spectroscopy

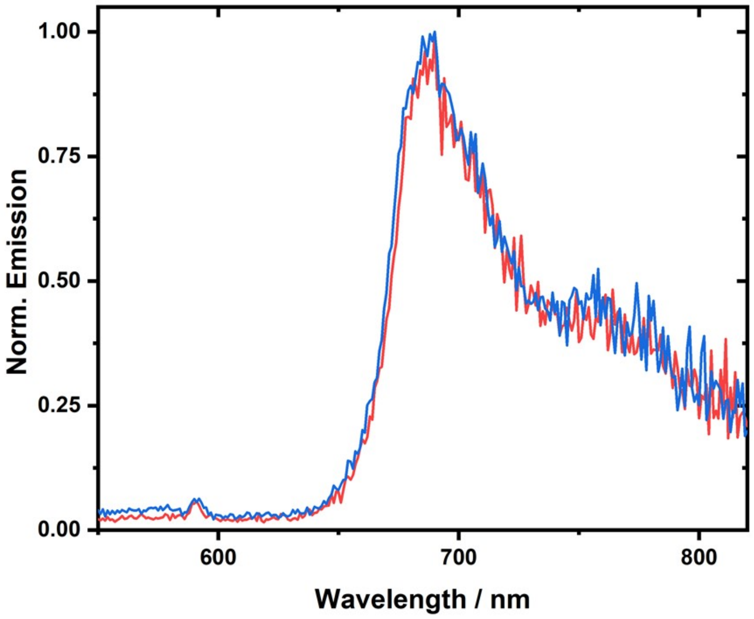

Fig.S1. Room temperature emission spectra of **2** and **3**, in aerated hexane, following excitation at 504 nm.

2.2 Time-Resolved Broadband Fluoresence Upconversion (FLUP) of 1 and 2.

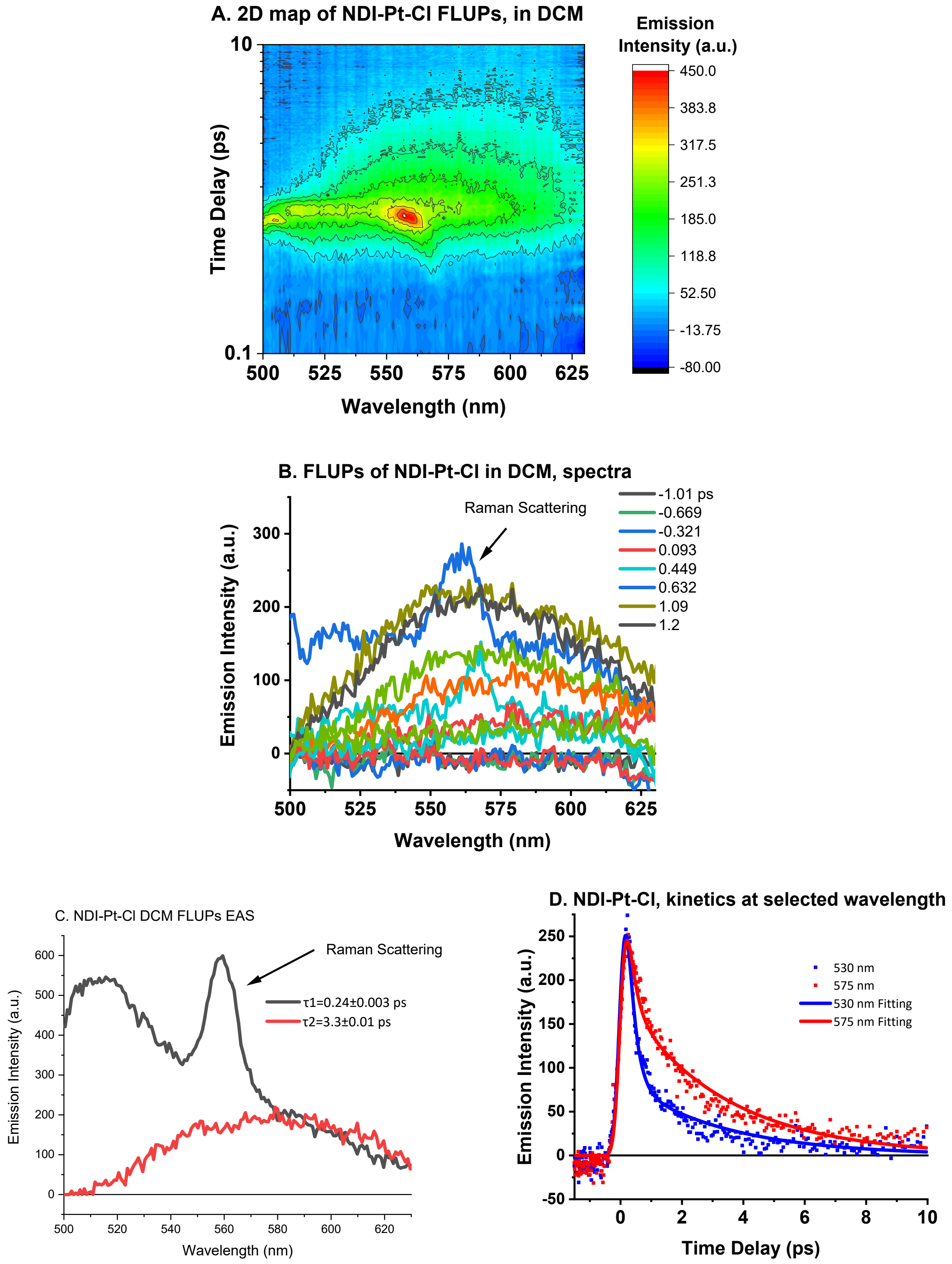

Fig.S2: Fluorescence Upconversion Spectroscopy (FLUPs) of **1** (NDI-Pt-Cl) in DCM, excitation 485 nm. A). 2D map. B). Spectra at stated time delays. C). Global analysis results: EAS spectra, and corresponding lifetimes. D). Kinetic traces at selected wavelength (dots) and fitted function (solid line) by Glotaran, where the Gaussian function for the IRF is used along with the two lifetimes given (0.2 and 3.3 ps). [Journal of Statistical Software, June 2012, Volume 49, Issue 3; 10.18637/jss.v049.i03; I. H. M. van Stokkum, D. S. Larsen and R. van Grondelle, Biochim Biophys Acta Bioenerg, 2004, 1657, 82–104; http://www.nat.vu.nl/~jsnel/pub/2011/20110223-MScThesis_SnellenburgJ.pdf]

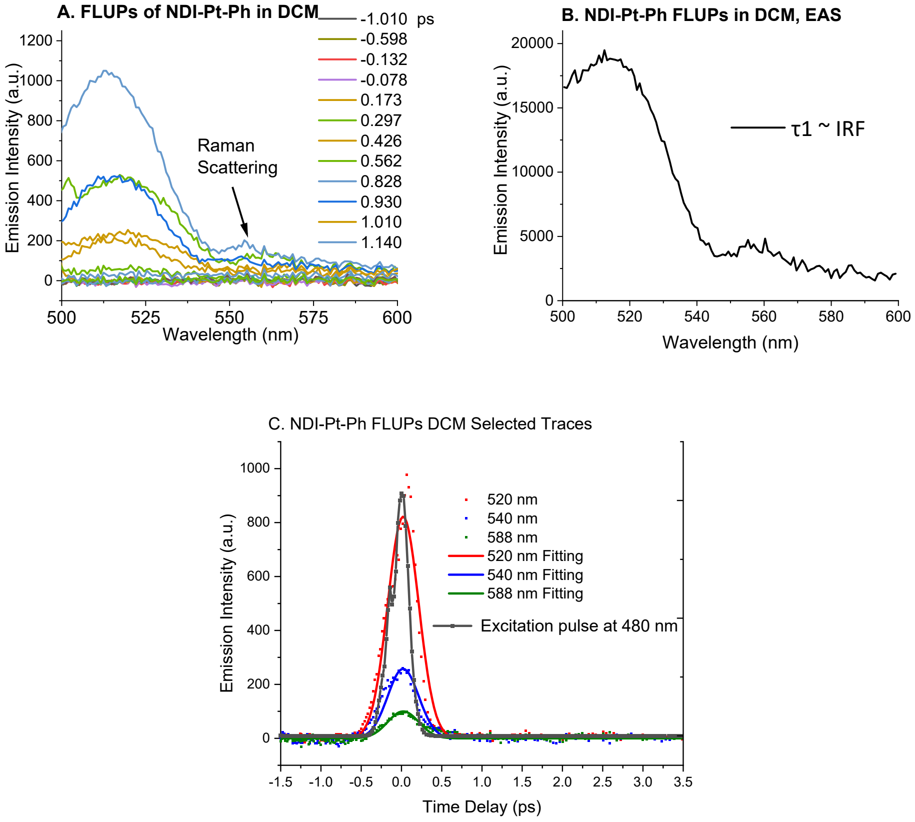

Fig.S3: A). FLUPs spectra of NDI-Pt-Ph, **2** in DCM, excitation 480 nm; B). EAS extracted by GLA gives a kinetic trace with lifetime shorter than FLUPs setup IRF (200 fs FWHM) and C). Kinetic traces (dots) and fitting trace (solid line) of FLUPs of **2** which shows a Gaussian distribution similar to the excitation pulse signal at 480 nm (dots with solid line).

2.3 Cyclic Voltammetry

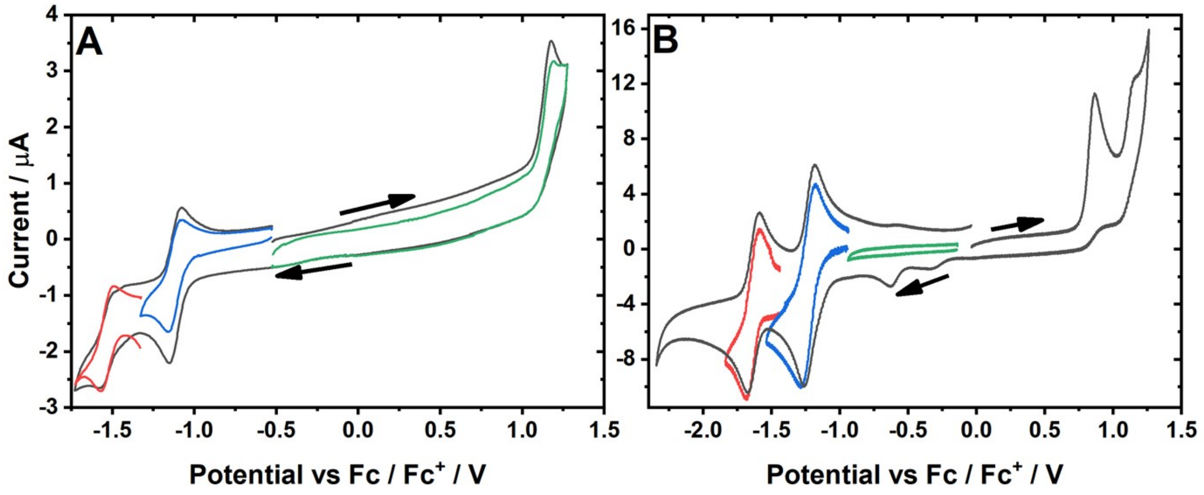

Fig. S4. Cyclic voltammograms of complex 1 (panel A) and 2 (panel B), in a dry DCM solution containing 0.2 mol dm^{-3}[NH4][PF6], recorded at room temperature at a scan rate of 100 mV s^{-1}. The blue and red traces are isolated scans of the first and second reduction processes, respectively.

2.4 UV-vis spectroelectrochemistry and comparison with late-time TA of 3

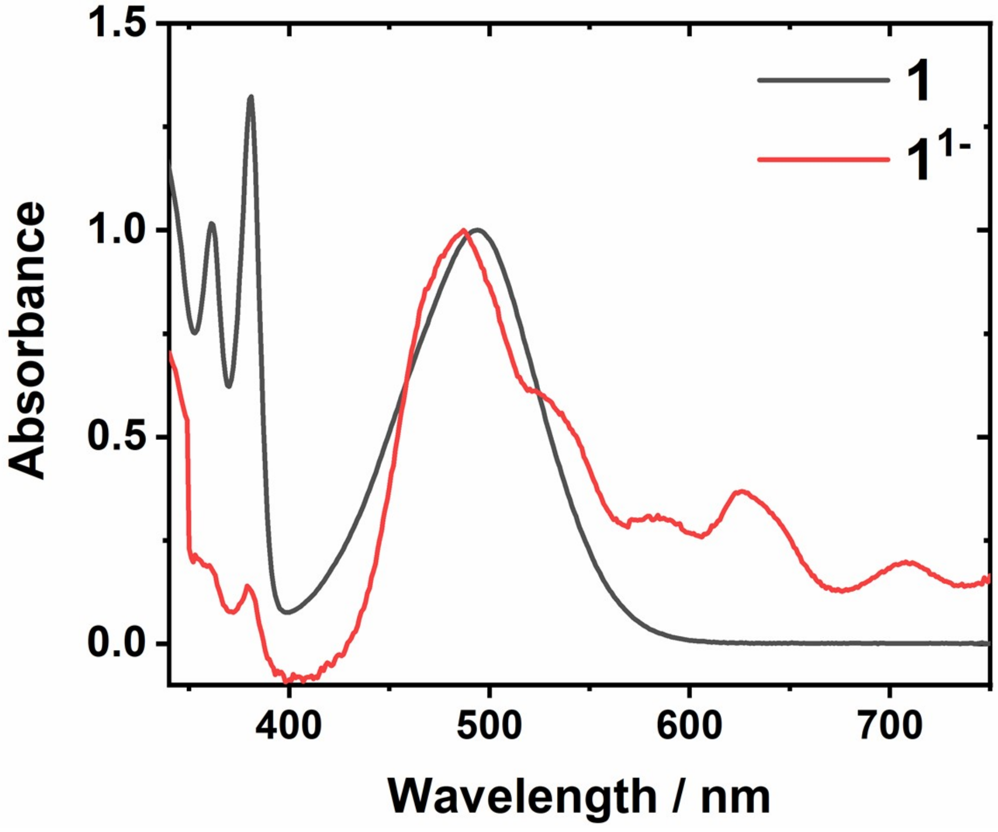

Fig.S5a. UV-vis absorption spectra of 1 and the 1e$^-$ reduced species of 1, in DCM, in the region 340 – 750 nm.

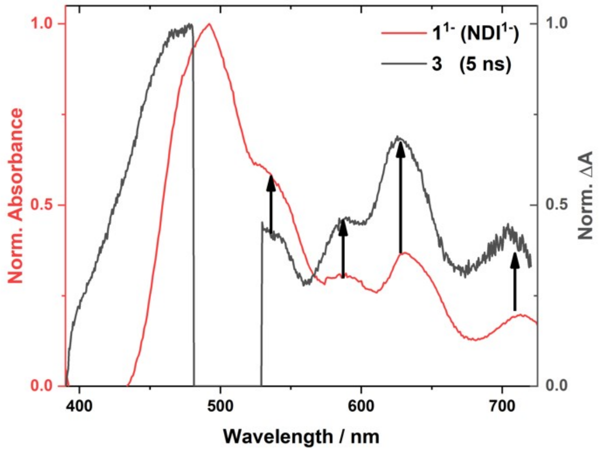

Fig. S5b. A plot of the late time (5 ns) TA spectrum of **3**, following excitation at 500 nm in DCM, along with the UV-Vis absorption spectrum of 1^{1-}.

2.5 Time-resolved Infrared Spectroscopy

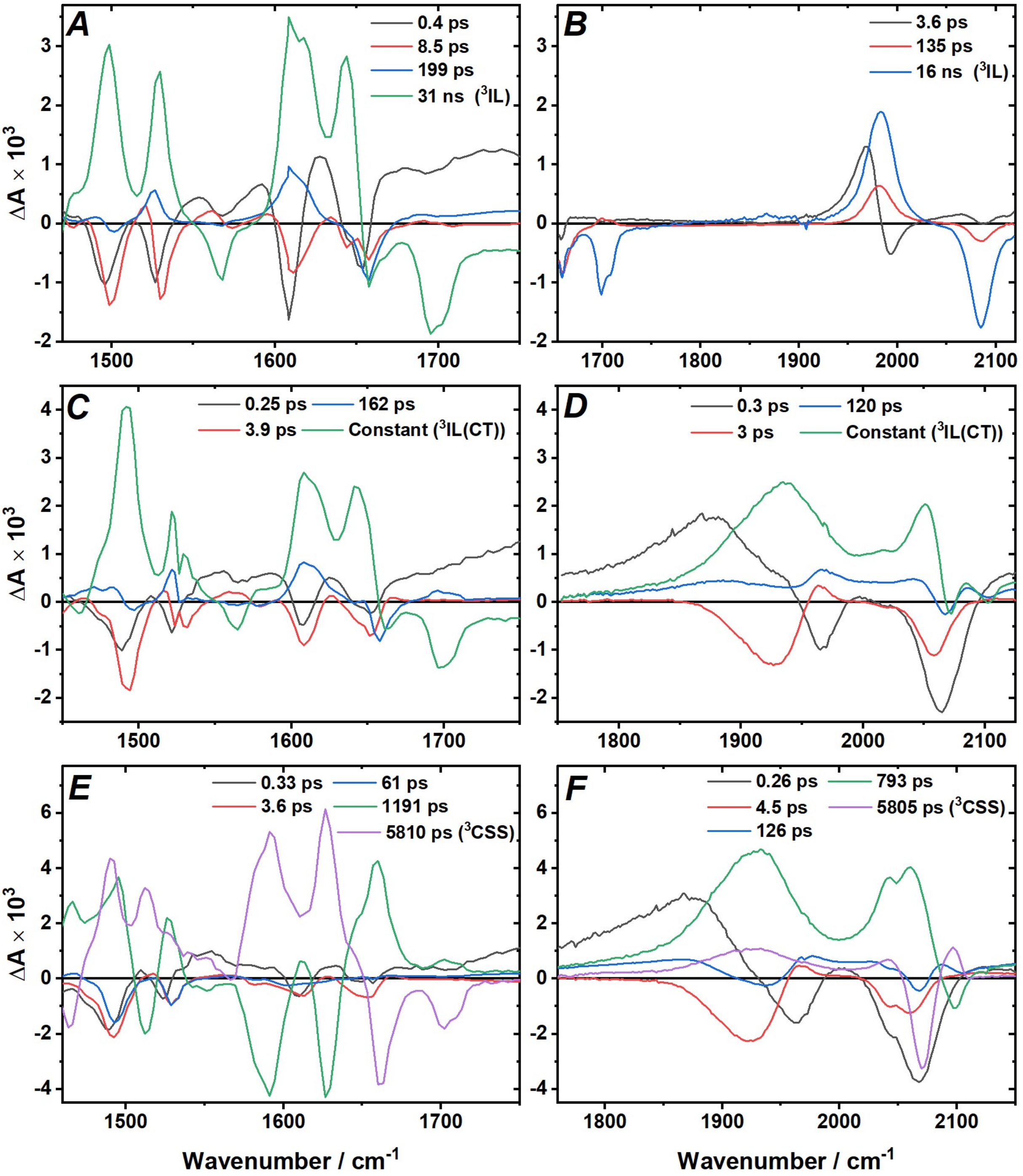

Fig.S6. DAS of the carbonyl and acetylide region TRIR spectra (in DCM) of complex 1, 2 and 3. The associated decay lifetimes are given as insets.

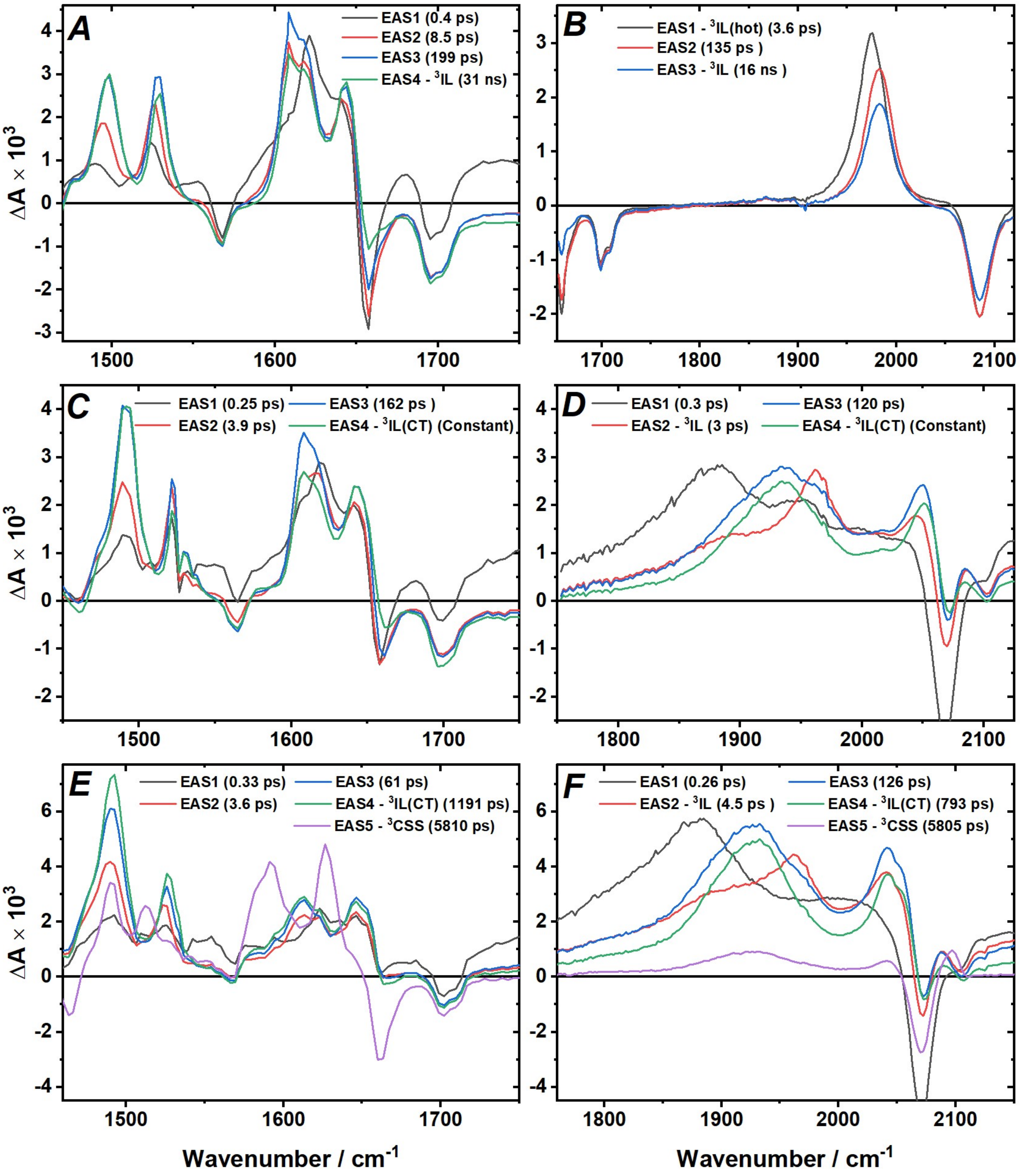

Fig.S7. EAS of the carbonyl and acetylide region TRIR spectra (in DCM) of complex 1, 2 and 3. The associated decay lifetimes are given as insets.

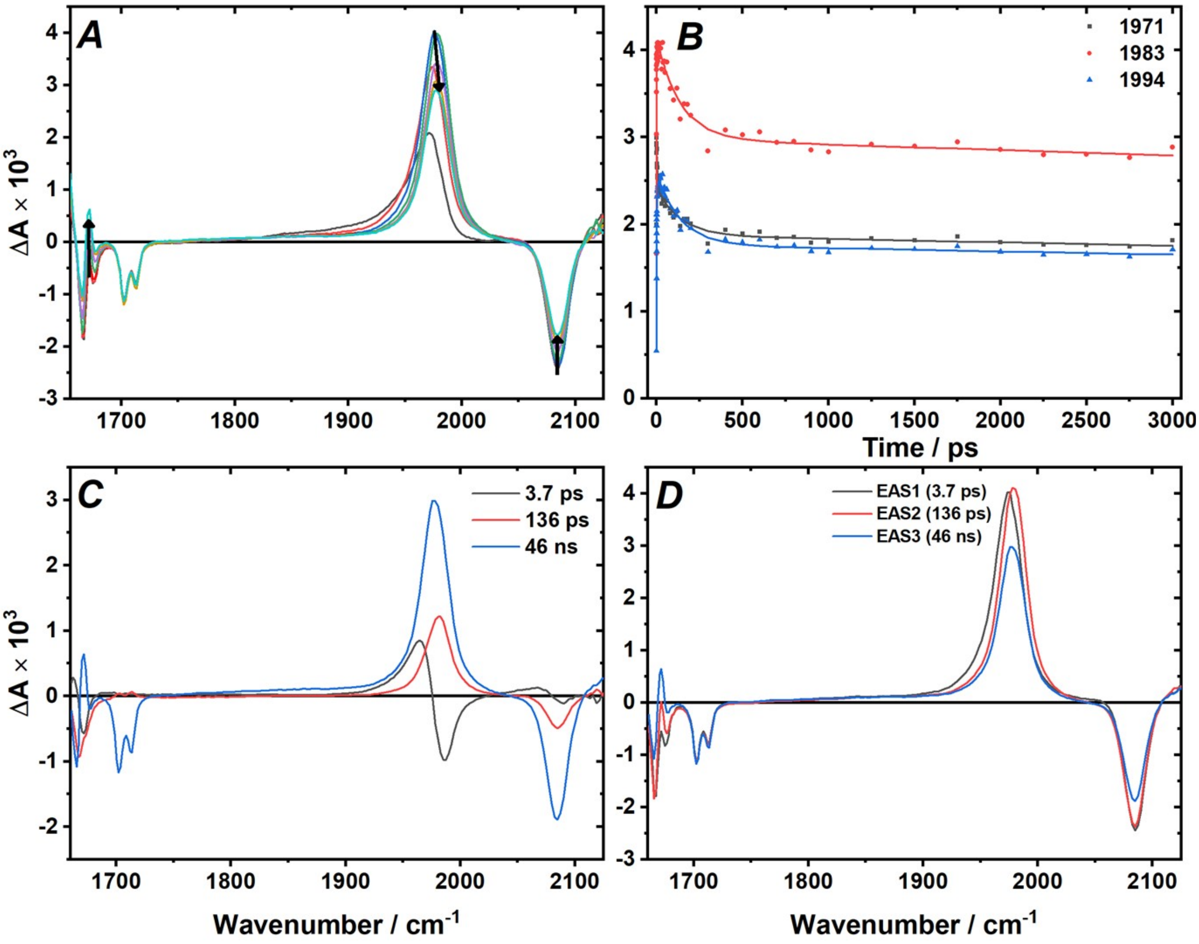

Fig.S8. Panel A: TRIR spectra of complex 1 in the acetylide region, following excitation at 520 nm in hexane. The time delays shown are: 0.4, 0.6,2, 12, 100, 600 and 3000 ps. Panel B: Single point kinetics traces for select wavelengths for complex 1. The wavelengths are given as insets. The overlaid solid traces are the global fittings obtained from an analysis of the TRIR dataset. Panel C: DAS of the TRIR dataset of complex 1 (in hexane). The associated lifetimes are given as insets. Panel D: EAS of the TRIR dataset of complex 1 (in hexane).

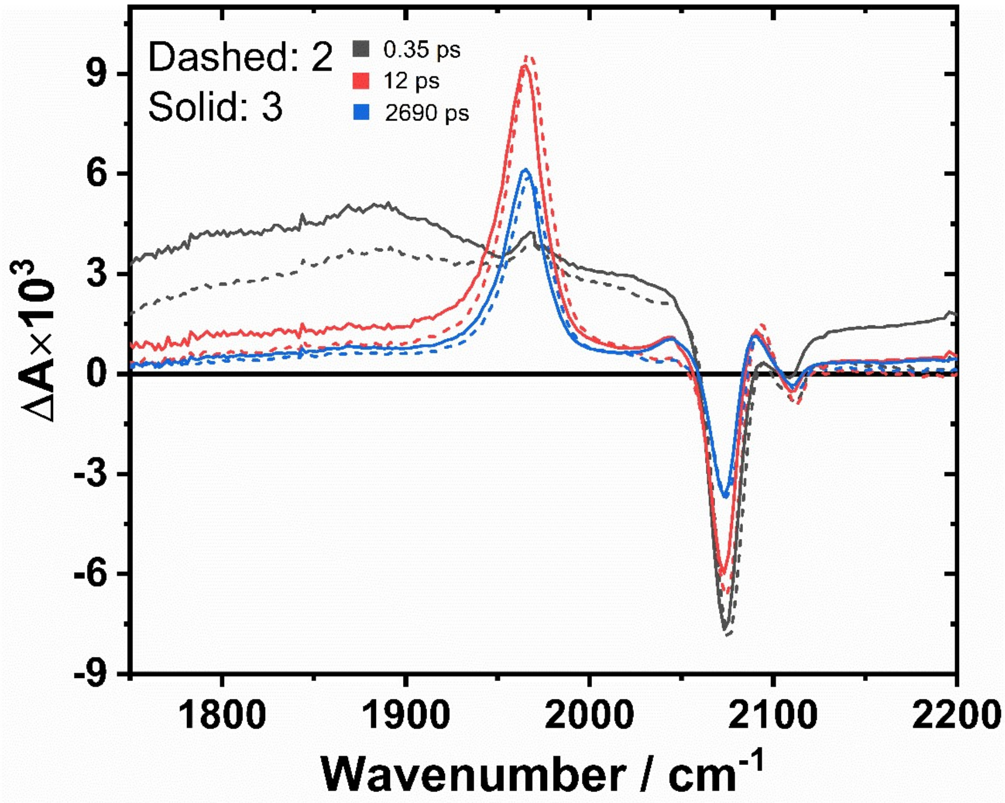

Fig.S9. The TRIR spectra of **2** and **3**, in hexane, at pump-probe delays of 0.35, 12 and 2690 ps.

Lifetime density analysis of TRIR data for 3

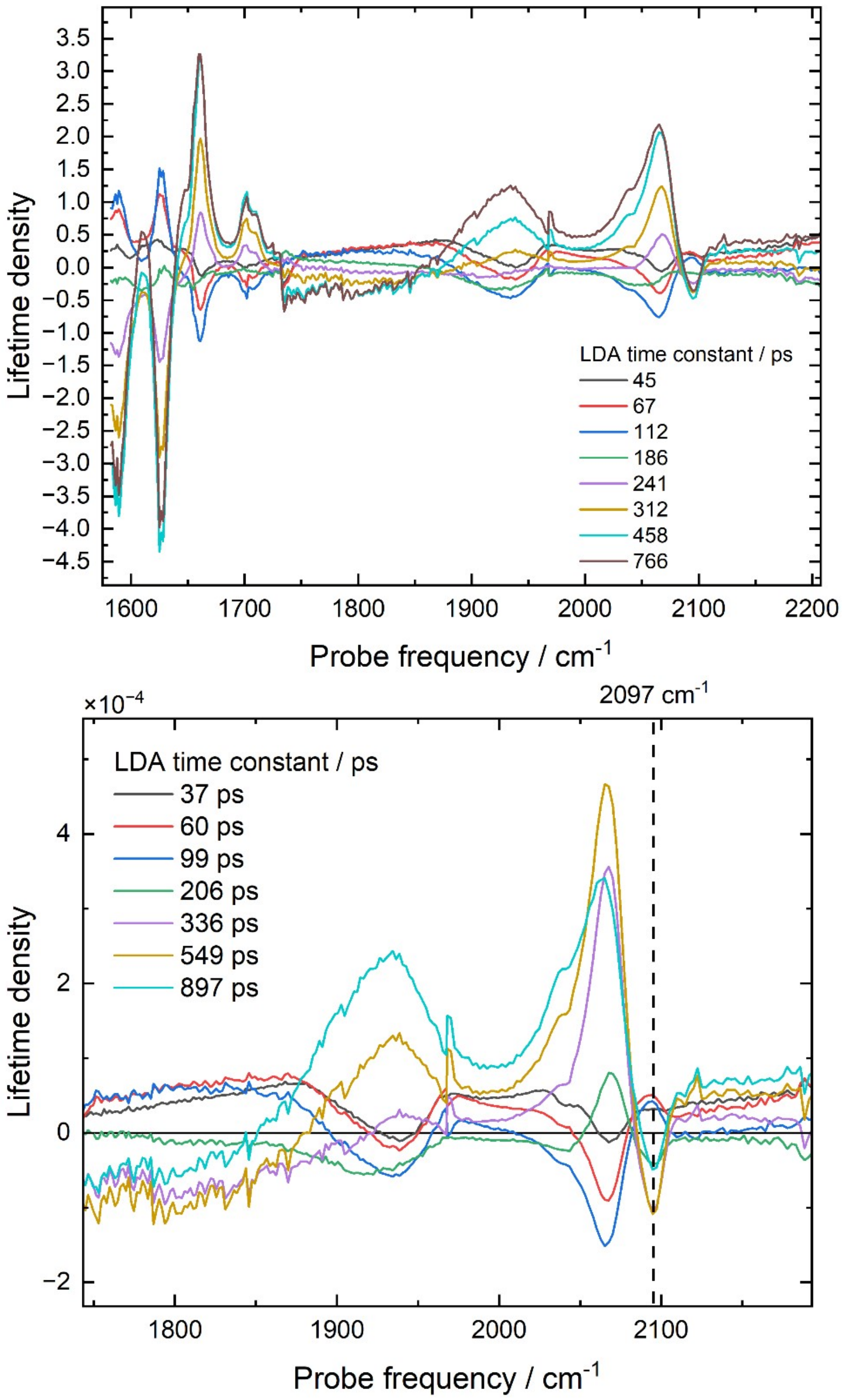

Fig. S10a. Lifetime density analysis of the TRIR data for 3. Selected traces for the full frerquency region (top), and expanded high-frequency (ν(CC)) region, bottom.

The procedure is described in: Slavov, C.; Hartmann, H.; Wachtveitl, J. Implementation and Evaluation of Data Analysis Strategies for Time-Resolved Optical Spectroscopy. Anal. Chem. 2015, 87 (4), 2328–2336. https://doi.org/10.1021/ac504348h.

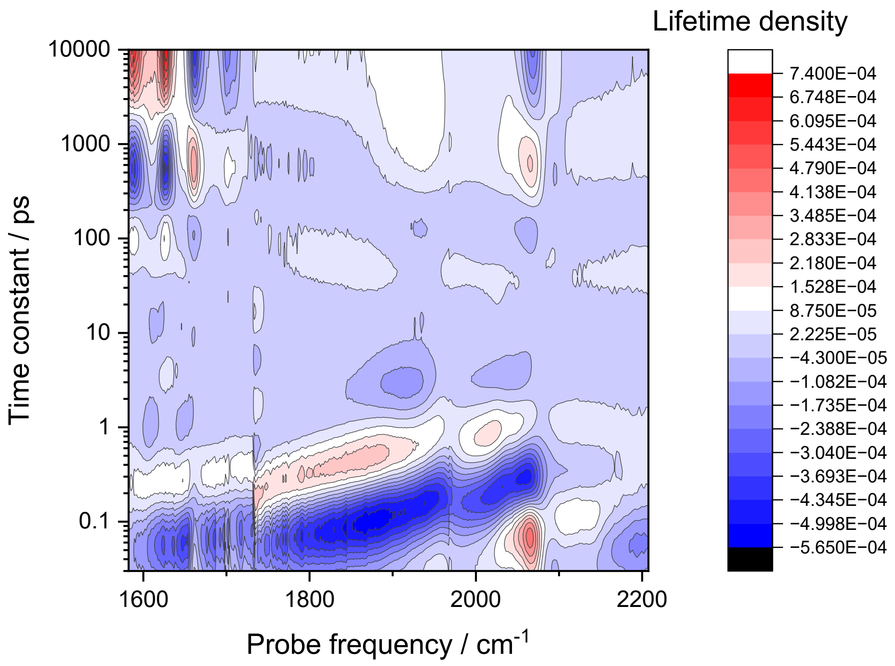

Fig. S10b. Lifetime density analysis of the TRIR data for 3. 2D map of the density of the lifetimes in the full spectral range, 1580 – 2200 cm^{-1}, and full scale of the time constants, up to 10 ns. (note, time scale is shown in log scale). Middle: 2D map of the density of the lifetimes (probability of a specific time constant to occur at a specific wavenumber) for the lower-frequency region 1580 – 1700 cm^{-1}, and up to 250 ps.

Slavov, C.; Hartmann, H.; Wachtveitl, J. Implementation and Evaluation of Data Analysis Strategies for Time-Resolved Optical Spectroscopy. Anal. Chem. 2015, 87 (4), 2328–2336. https://doi.org/10.1021/ac504348h.

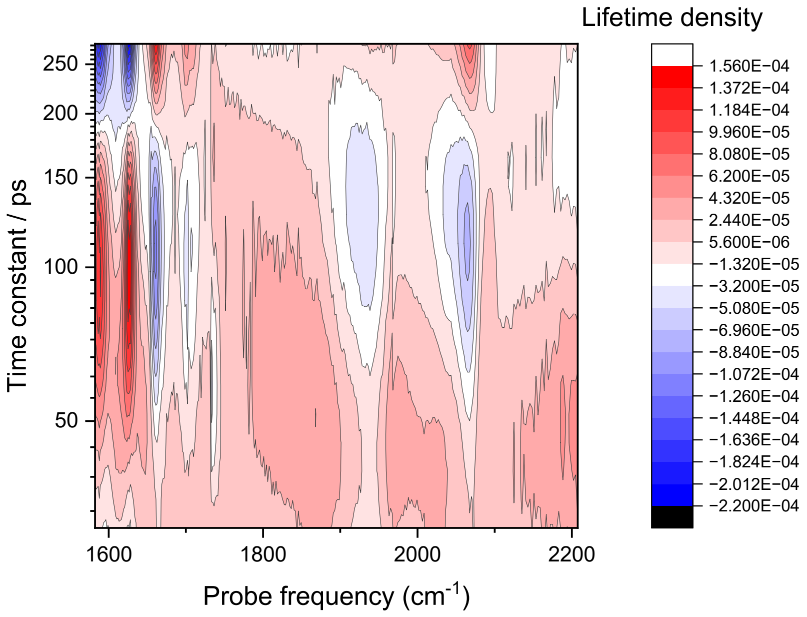

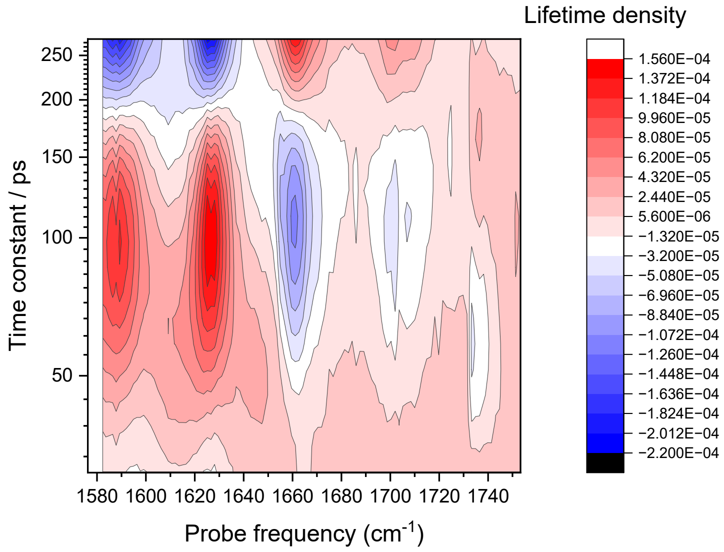

Fig. S10c. Lifetime density analysis of the TRIR data for 3. 2D map of the density of the lifetimes in the range 0 – 250 ps (probability of a specific time constant to occur at a specific wavenumber).

Bottom: expanded the lower-frequency region 1580 – 1700 cm^{-1}, and up to 250 ps.

Top: full spectra range, including low- and high frequency regions, 1580 – 2200 cm^{-1}.

Slavov, C.; Hartmann, H.; Wachtveitl, J. Implementation and Evaluation of Data Analysis Strategies for Time-Resolved Optical Spectroscopy. Anal. Chem. 2015, 87 (4), 2328–2336. https://doi.org/10.1021/ac504348h.

2.6 Transient absorption

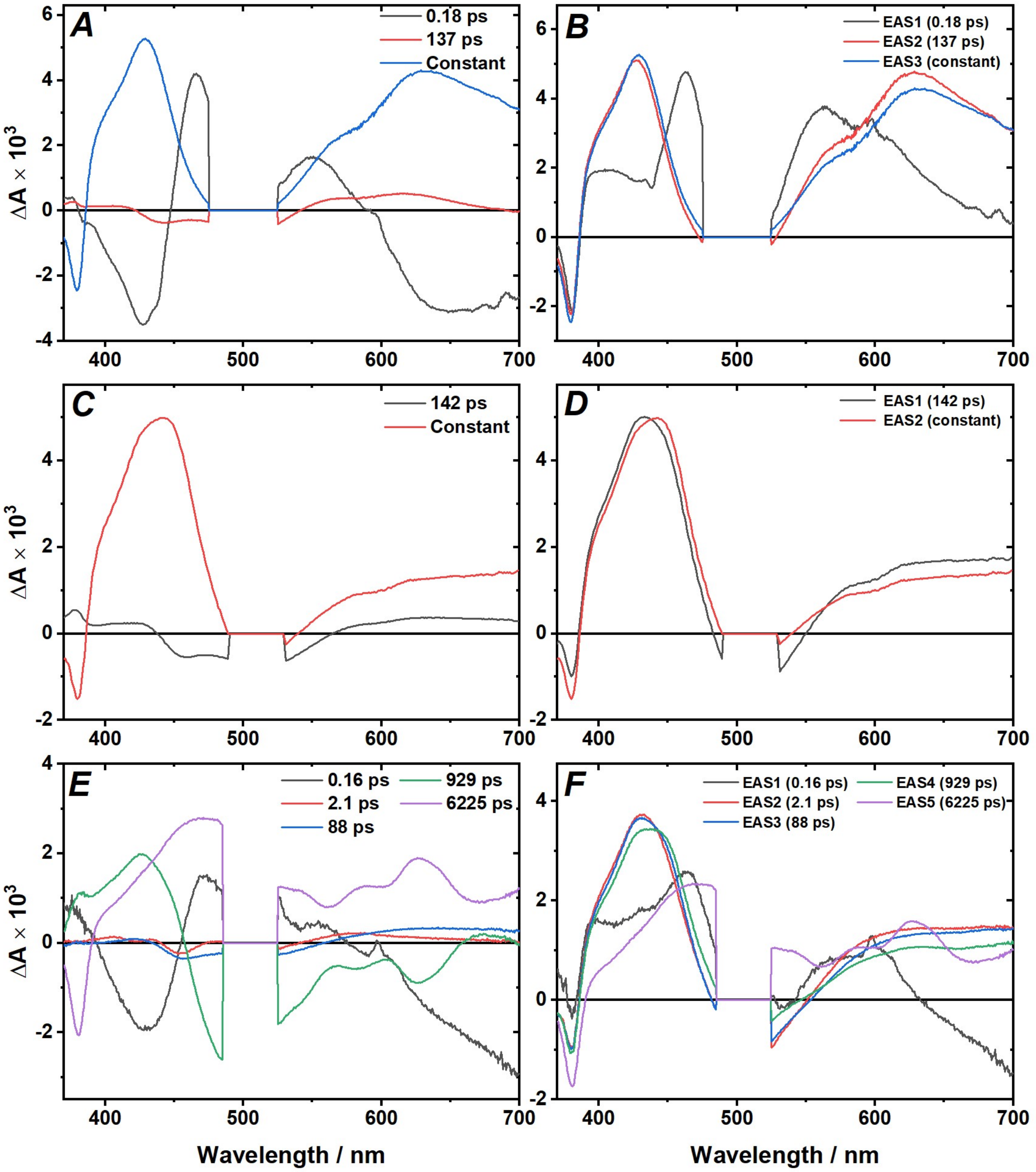

Fig.S11. DAS of the TA spectra (in DCM) of complex 1, 2 and 3 (panel A, C and E, respectively). The associated lifetimes are given as insets. The EAS are also shown for complex 1, 2 and 3 (panel B, D and F, respectively).

2.7 Nanosecond Flash Photolysis

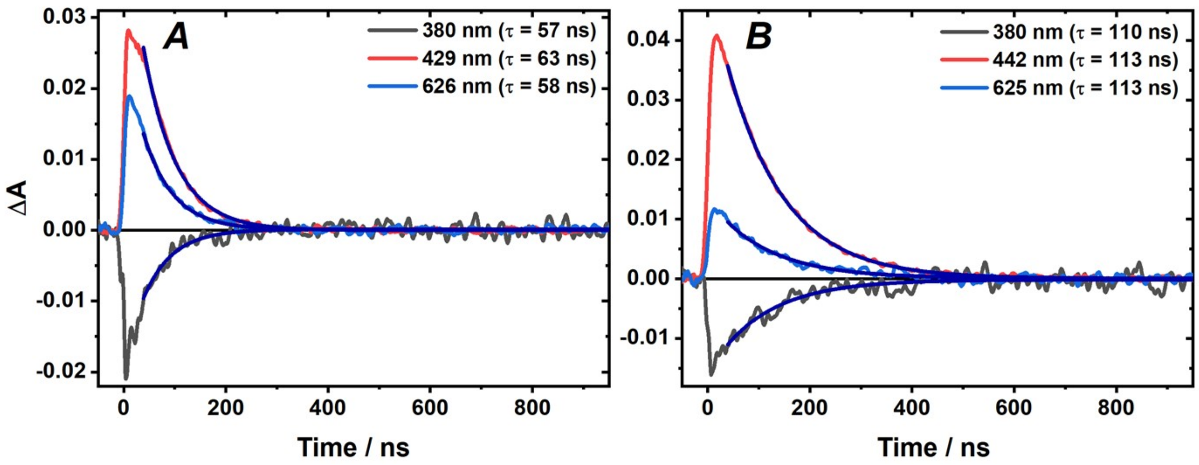

Fig.S12a. Single point kinetic traces at various wavelengths, for complex 1 and 2 (panel A and B, respectively), following excitation at 500 nm in DCM. The solid blue traces overlaid are monoexponential tail fits. The wavelengths and corresponding lifetimes are given in the inset. (the lifetime is estimated as 112 ns ± 5 ns from these data).

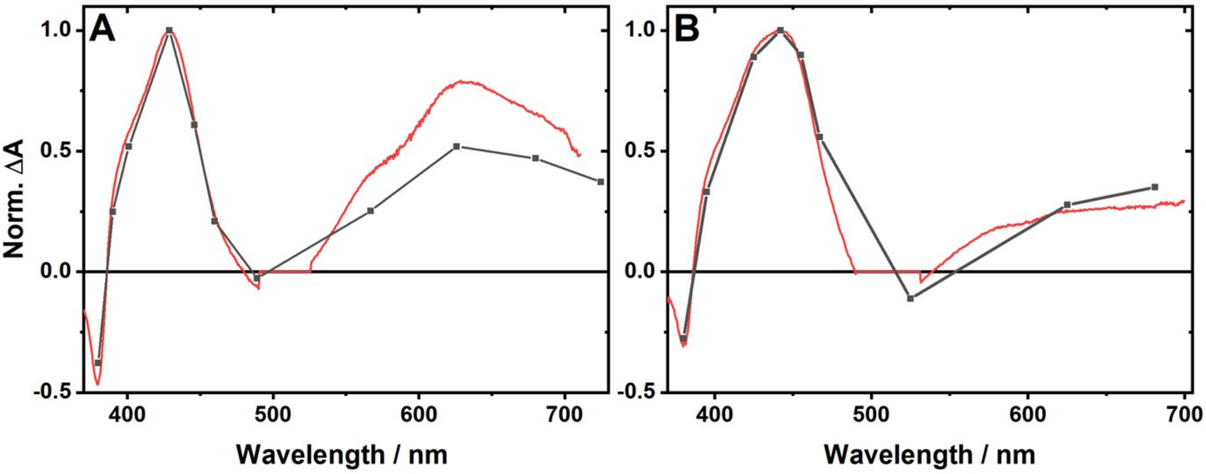

Fig.S12b. Late- and early-time transient UV-vis spectra of complex 1 and 2 (panel A and B, respectively), following excitation at 500 nm in DCM. The red traces are the spectra at 3 ns (TA) and the black traces are the spectra at 50 ns (Flash Photolysis).

2.8 Summary of time constants

Complex	*τ/ps (TA)*	*τ/ps (LF-TRIR)*	*τ/ps (HF-TRIR)*	*τ/ps (FLUPS)*
1	0.18	0.4	-	0.24
NDI-Pt-Cl	-	8.5	3.6	3.3
	137	199	135	
	60 ns[a]	31 ns	16 ns	
2	-	0.25	0.3	<IRF[c]
NDI-Pt-Ph	-	3.9	3	
	142	162	120	
	114 ns[a]	fixed[b]	fixed[b]	
3	0.16	0.33	0.26	
NDI-Pt-PTZ	2.1	3.6	4.5	
	88	61	126	
	929	1191	793	
	6225	5810	5805	

Table S1. A summary of the lifetimes obtained from GLA fittings of the TA and TRIR data of complexes **1, 2** and **3**.

[a]Lifetimes obtained from a monoexponential tail fitting of transient absorption decay traces obtained from flash photolysis experiments. [b]Using a fixed lifetime obtained from flash photolysis. [c]GLA extracted a 0.02 ps component which indicates this trace lifetime is shorter than IRF of FLUPs setup.

2.9 Scheme S1. Comparison of energy level diagrams and timescales for compound 3, NDI-Pt-PTZ (left side) and its NAP analog, NAP-Pt-PTZ.

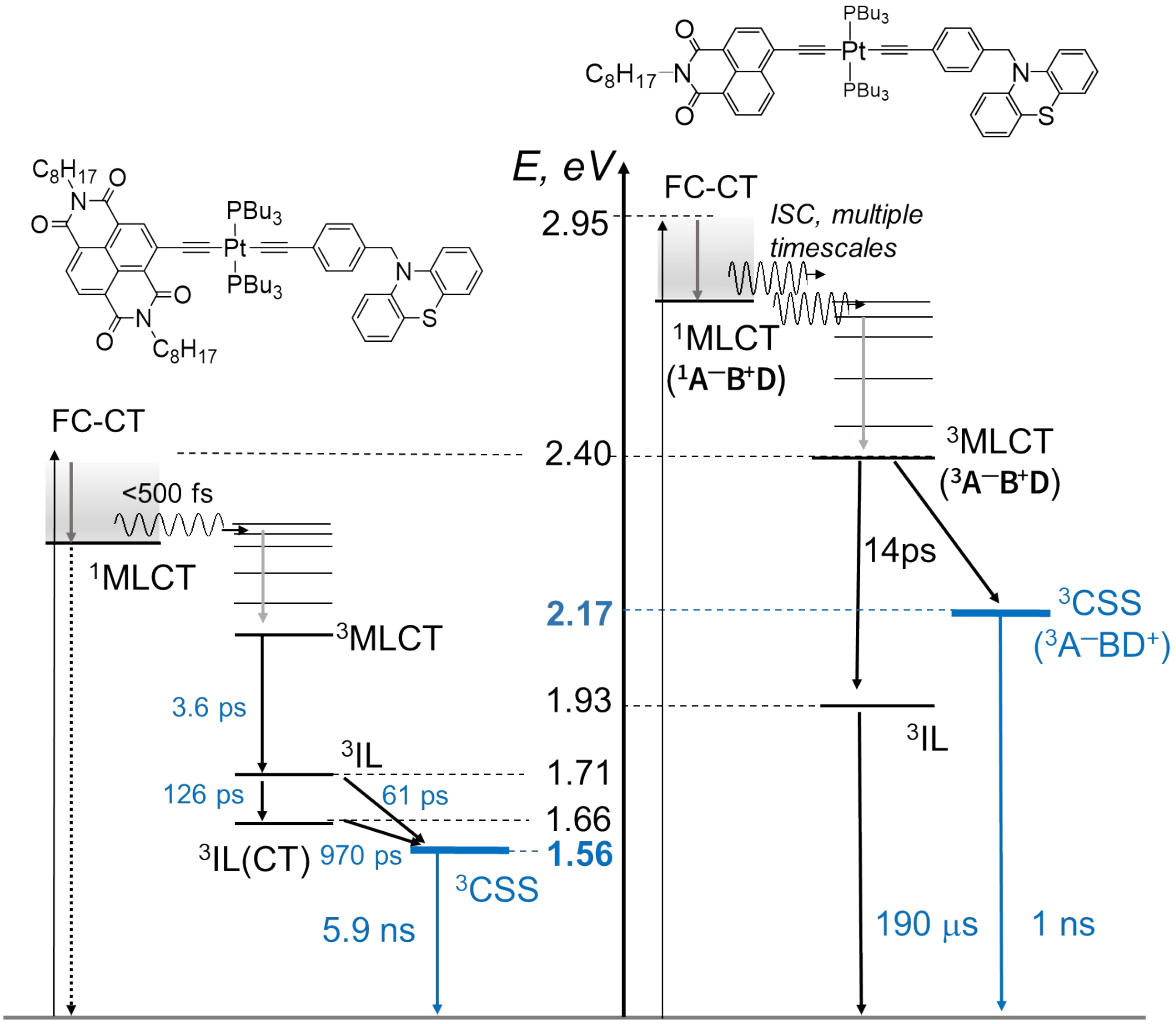

Scheme S1.

Comparison of energy level diagrams and timescales for compound 3, NDI-Pt-PTZ (left side) and its NAP analog, NAP-Pt-PTZ.

Data for NAP-Pt-PTZ taken from ref. 2, P. A. Scattergood, M. Delor, I. V. Sazanovich, O. V. Bouganov, S. A. Tikhomirov, A. S. Stasheuski, A. W. Parker, G. M. Greetham, M. Towrie, E. S. Davies, A. J. H. M. Meijer and J. A. Weinstein, *Dalt. Trans.*, 2014, **43**, 17677–17693.

3 Further experimental information

Global Lifetime Analysis (GLA)

Global lifetime analysis (GLA)[15] of the data was performed using Glotaran 1.5.1. A free software package that serves as a GUI for the R-package TIMP. Details of the software are described elsewhere.[16,17] Such software packages fit the kinetic traces with a sum of decaying exponential functions, like those given below, convoluted with a gaussian function to model the instrument response function.

$$\Delta A(t,\lambda_i) = \sum_{n=1}^{n_{comp}} B_n(\lambda_i) e^{-t/\tau_n} = B_1(\lambda_i) e^{-t/\tau_1} + B_2(\lambda_i) e^{-t/\tau_2} \ldots$$

The total number of decaying exponential functions summed is given by n_{comp}, i.e., the number of components. Such a function is then convoluted with an IRF function.

Time-Resolved Infrared Spectroscopy

A fraction of the amplifier output was used to pump a fs-NOPA TOPAS which was used to provide 520 nm excitation pulses. The probe and pump beams were focussed onto a Harrick cell (CaF_2 windows, 630 μm internal path) to yield spot sizes of 70 μm and 120 μm at the sample, respectively. The sample solution was flowed through the cell using a peristaltic pump, while the cell was simultaneously raster-scanned to avoid decomposition. The relative polarisation between the pump and probe was set to magic angle. The IR light transmitted through the sample was recorded using two, linear, 128 array HgCdTe detectors (Infrared Associates). The data was analysed using the same software packages described above.

Nanosecond flash photolysis

500 nm excitation pulses (7 ns FWHM, 10 Hz, 2.5 mW) were provided by a Beta-Barium Borate optical parametric oscillator (LT-2214OPO), which was pumped by the 355 nm (7 ns FWHM, 10 Hz, 56 mJ) output of a Q-switched Nd:YAG laser (LS-2137U). Sample probing was performed with a steady state 150 W Hamamatsu arc Xe lamp. The probe light transmitted through the sample was passed through a monochromator and detected with a home-built unit based on a FEU 118 PMT, coupled to a Tektronix TDS 3032B digital oscilloscope. Sample solutions were prepared in 1 cm quartz cells.

Cyclic Voltammetry

Experiments were performed using an Autolab Potentiostat 100. The measurements were taken in a glass sample tube using a glassy carbon working electrode, a platinum wire counter electrode and an Ag/AgCl (0.1 mol dm^{-3}) reference electrode. Solutions of the analyte were prepared with 0.2 mol dm^{-3} $[NBu_4][PF_6]$ supporting electrolyte in dry DCM and saturated with N_2. All potentials were quoted relative to the ferrocene/ferrocenium couple (Fc/Fc^+).

IR and UV-Vis Spectroelectrochemistry

Measurements were carried out using an Autolab Potentiostat. Solutions of the analyte were prepared with 0.2 mol dm^{-3} $[NBu_4][PF_6]$ supporting electrolyte in dry DCM. Measurements were performed in an optically transparent thin-layer electrochemical (OTTLE) cell, with platinum mesh working and counter electrodes, a silver wire pseudoreference electrode and CaF_2 windows. IR and UV-vis spectra were recorded using a PerkinElmer Spectrum One FTIR spectrometer and an Agilent Cary 5000 UV-vis-NIR spectrometer, respectively.

4 Theoretical results

4.1 Simulated electronic absorption spectra

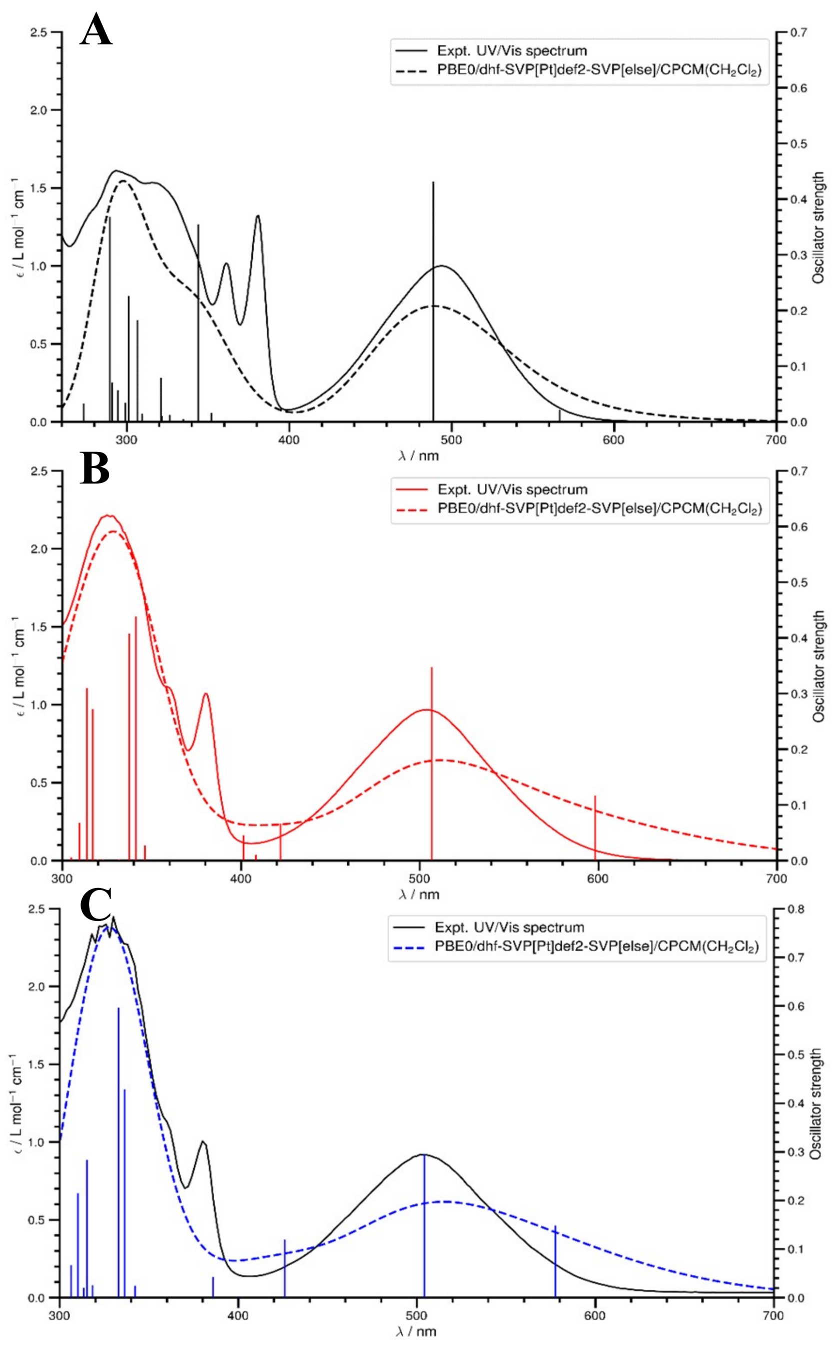

Fig.S13. Experimental electronic absorption spectra (solid traces) of complex 1, 2 and 3 (panel A, B and C, respectively) and the calculated electronic spectra of the ground state at the PBE0//dhf-SVP[Pt]-SVP[else]CPCM(CH_2Cl_2) (dashed traces).

4.2 Simulated vibrational absorption spectra

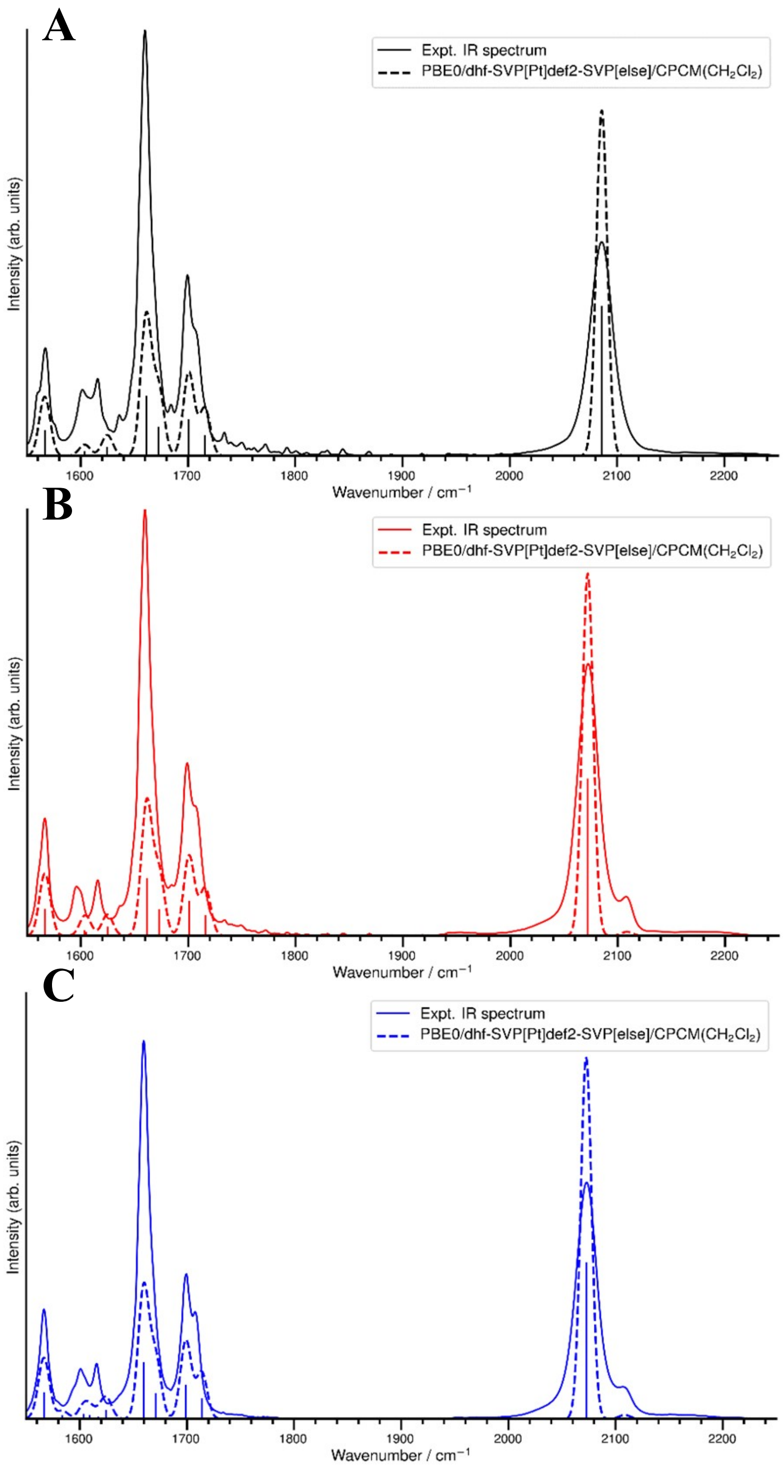

Fig.S14. Experimental vibrational absorption spectra (solid traces) of complex 1, 2 and 3 (panel A, B and C, respectively) and the calculated vibrational spectra of the ground state at the PBE0//dhf-SVP[Pt]-SVP[else]CPCM(CH_2Cl_2) (dashed traces). Scaling factors are as follows: 0.955 (1450 – 1729 cm^{-1}) for A, B and C / 0.94 (1730 – 1850 cm^{-1}) for A and B, 0.939 (1730 – 1850 cm^{-1}) for C / 0.955 (1851 – 2300 cm^{-1}) for A, B and C.

4.3 Density difference plots of the first 4 singlet excited states vs. the ground state in 1-3

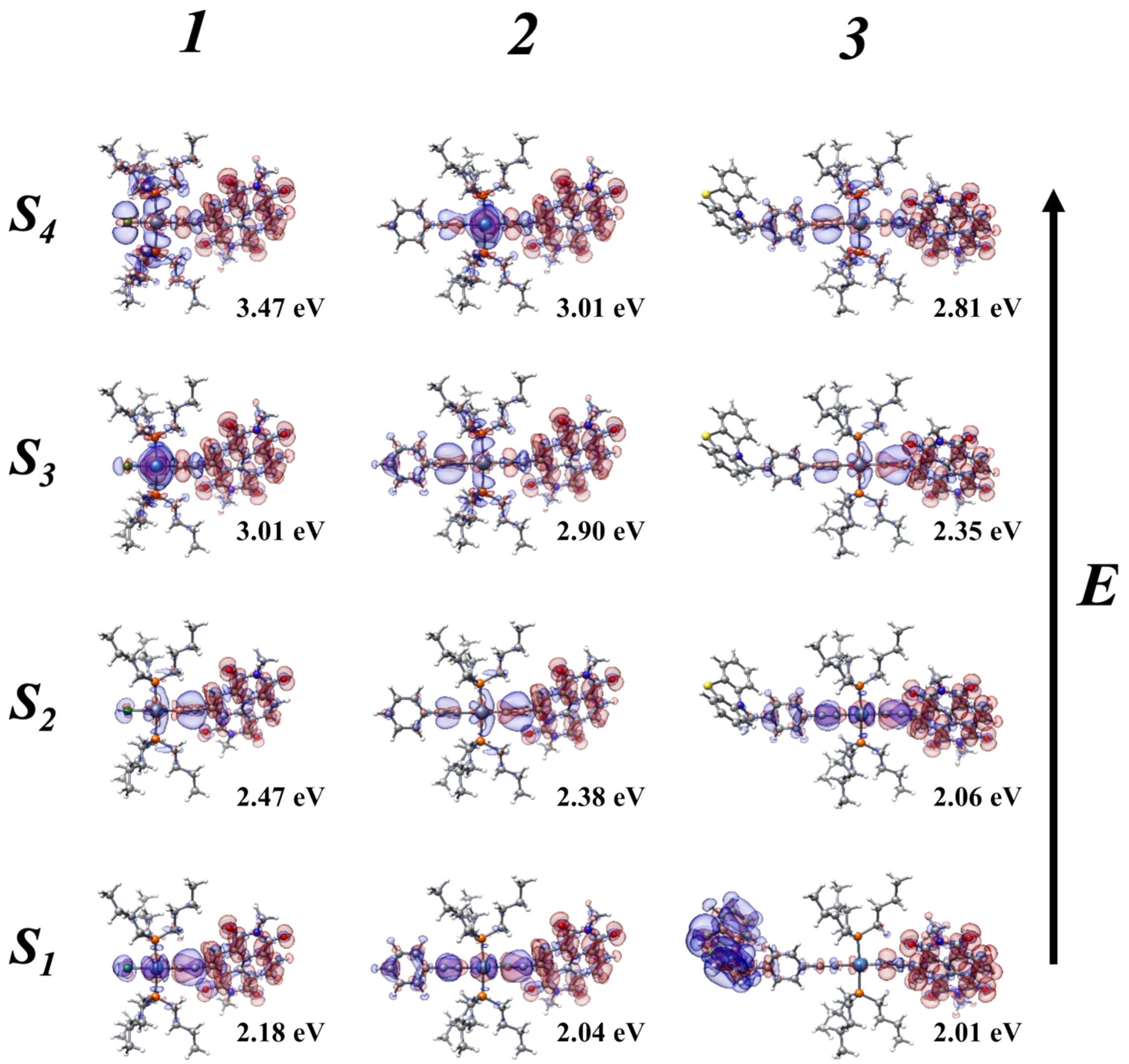

Fig.S15. Electron density difference plots of the first four singlet excited states of complex 1, 2 and 3. The electron density difference is relative to the ground state electron density distribution. Blue shaded regions represent a loss in electron density (relative of the ground state), while red shade regions represent an increase in electron density. For 1, 2 and 3, the geometries are the ground state optimised geometries.

4.4 Density difference plots on the lowest 4 triplet states relative to the ground state.

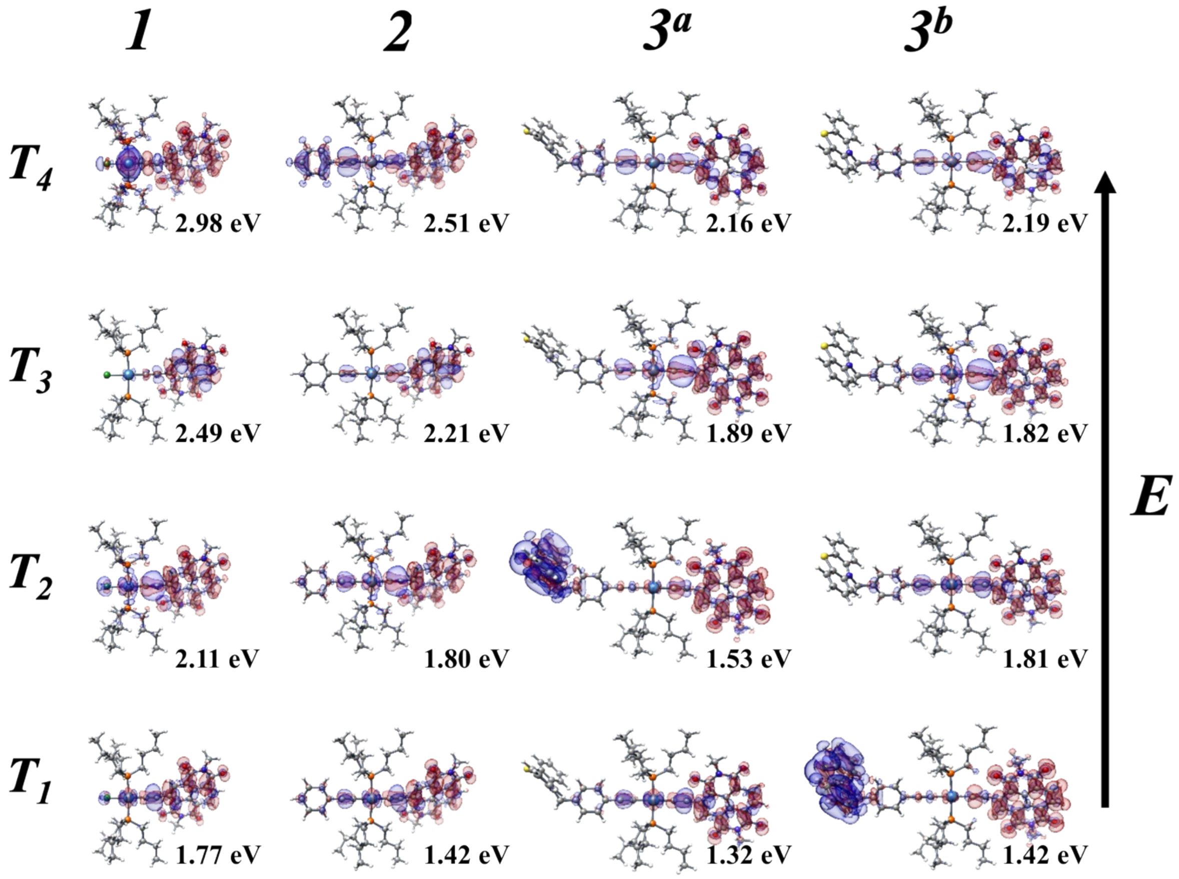

Fig.S16. Electron density difference plots of the first four triplet excited states of complex **1 - 3**. The electron density difference is relative to the ground state electron density distribution. Blue-shaded regions represent a loss in electron density (relative of the ground state), while red-shaded regions represent an increase in electron density. For 1, 2 and 3[a], the geometries are the ground state optimised geometries. For 3[b] the PTZ donor group is planar, unlike in the ground state optimised geometry where it is puckered.

4.5 Experimental ground state and excited state vibrational spectra overlaid with calculated spectra, and electron density maps

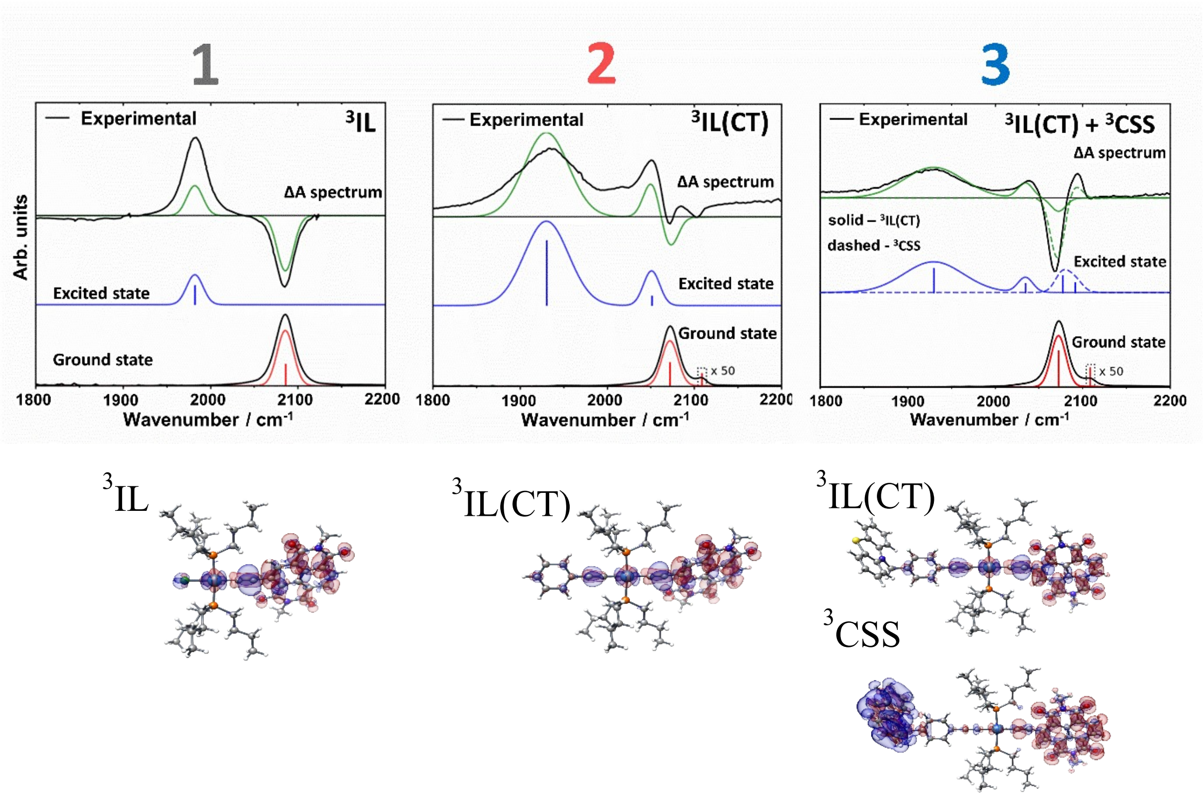

Fig.S17. Experimental ground state and excited state vibrational spectra, in the region 1800 – 2200 cm^{-1}, overlaid with the simulated spectra, for **1–3**. The experimental TRIR difference spectra are at a pump-probe delay of 2.7 ns. The density difference plots associated with the excited states responsible for the difference spectra signal are also given below.

5 Additional computational details

1: Cl-Pt-CC-NDI in CH_2Cl_2 SINGLET

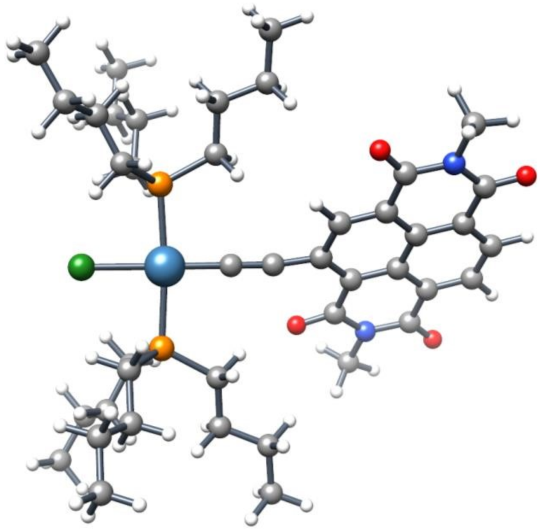

Optimised singlet ground state geometry of Cl-Pt-CC-NDI.

Route	:	#p opt freq pbe1pbe/genecp
	:	scrf=(cpcm,solvent=dichloromethane) geom=connectivity
	:	int=(acc2e=13,ultrafine)
SMILES	:	CCCC[P](CCCC)(CCCC)[Pt]
	:	(C#Cc1cc2c3c(ccc4c3c1C(=O)N(C4=O)C)
	:	C(=O)N(C2=O)C)([P](CCCC)(CCCC)CCCC)Cl
Formula	:	$C_{42}H_{63}ClN_2O_4P_2Pt$
Charge	:	0
Multiplicity	:	1
Dipole	:	7.9749 Debye
Energy	:	-3306.83205822 a.u.
Gibbs Energy	:	-3305.95602200 a.u.
Number of imaginary frequencies	:	0

Cartesian Co-ordinates (XYZ format)

115

C	2.22091389	3.12823892	1.95260203
H	1.95422304	2.28128290	2.60740089
H	3.31605506	3.06996202	1.83123505
C	1.28287005	-3.06246591	-0.56084698

H	0.85339999	-2.87986398	-1.56183600
H	0.51067001	-2.73095608	0.15225200
C	3.62706304	-1.87297702	-1.95727301
H	3.07018709	-1.20449495	-2.63584089
H	4.55769587	-1.33255196	-1.71464503
C	3.73509693	-2.39351702	0.99393398
H	4.25764322	-1.47197998	1.29707801
H	3.04629993	-2.64829397	1.81864500
C	3.90610600	-3.19645596	-2.66140103
H	2.95303702	-3.68402195	-2.92843390
H	4.43106079	-3.89416003	-1.98935497
C	4.73544502	-3.00564599	-3.92804599
H	5.68982887	-2.51741290	-3.66314292
H	4.21333122	-2.30014610	-4.59830809
C	5.01110888	-4.30665207	-4.66558409
H	5.61129522	-4.13657618	-5.57240915
H	5.56290102	-5.01785707	-4.02981091
H	4.07422781	-4.79834795	-4.97381592
C	1.57511699	-4.54585123	-0.35468900
H	2.36299610	-4.89634991	-1.04108405
H	1.96212006	-4.70759487	0.66597700
C	0.32792601	-5.40384817	-0.54654199
H	-0.06538000	-5.24580097	-1.56636596
H	-0.45888099	-5.04490900	0.13922200
C	0.57909501	-6.88604689	-0.31741101
H	-0.33715600	-7.47825098	-0.46395901
H	1.34132802	-7.27542305	-1.01177704
H	0.93851399	-7.07735777	0.70676500
C	4.75490284	-3.50566602	0.76734602
H	4.26468801	-4.42552519	0.41021299
H	5.45902920	-3.20233703	-0.02608400
C	5.54581594	-3.82157207	2.03344393
H	6.02881813	-2.89744401	2.39681005
H	4.84447098	-4.12707996	2.82994008
C	6.59426117	-4.90414286	1.82978499
H	7.33020210	-4.60942793	1.06424797
H	7.14621019	-5.11087418	2.75936198
H	6.13452291	-5.84932899	1.49841797
C	1.79189897	4.43614006	2.60910010
H	0.69912797	4.43451691	2.76165509
H	2.00984097	5.29376316	1.95267797
C	2.47776294	4.65212584	3.95503902
H	2.26705003	3.78851104	4.61023188
H	3.57175112	4.65526390	3.80491590
C	2.04975009	5.93768787	4.64541721
H	2.27986908	6.81999588	4.02649307
H	0.96506900	5.94544077	4.84009790
H	2.56184793	6.06632090	5.61115122
C	-0.63362098	4.57259178	0.01659100
H	-0.18284599	5.23896313	0.76964903
H	-0.24126200	4.90210199	-0.96053398

C	-2.14832997	4.76047993	0.02842000
H	-2.60544705	4.09419107	-0.72431397
H	-2.54450989	4.42775106	1.00415397
C	-2.57397699	6.19669294	-0.23447400
H	-3.66957593	6.30104780	-0.21889900
H	-2.16325307	6.88202620	0.52462202
H	-2.22086000	6.54484987	-1.21866298
C	2.77916288	5.09706020	-0.72585100
H	3.45839095	5.14766693	0.14212300
H	1.88951302	5.68873501	-0.45676899
C	3.46326995	5.74459410	-1.92626798
H	4.34978008	5.14683819	-2.20208311
H	2.78549790	5.69603300	-2.79684091
C	3.87315702	7.18737221	-1.67513299
H	3.00062299	7.81446123	-1.43022394
H	4.57959414	7.26183891	-0.83259898
H	4.36125183	7.62722778	-2.55817103
C	0.12265100	-0.06847700	-0.06195900
C	-1.07681501	-0.36197200	-0.02418100
C	-2.48225689	-0.50028098	-0.07099600
C	-3.24210501	-1.48486495	0.59449297
C	-3.18443108	0.46932501	-0.85545099
C	-4.65801001	-1.48711395	0.48324999
C	-2.59545898	-2.52593803	1.41852498
C	-4.55167913	0.47638801	-0.96289802
H	-2.61316800	1.23343694	-1.38395202
C	-5.44656992	-2.45749998	1.14549696
C	-5.32240009	-0.50684500	-0.29741800
O	-1.39111197	-2.62080193	1.56279099
N	-3.42353296	-3.45166397	2.06291103
C	-5.22045708	1.52290905	-1.77384496
C	-4.80933619	-3.49836898	1.98130095
C	-6.82927084	-2.44771194	1.02620900
C	-6.72630405	-0.51171798	-0.40711999
C	-2.74743295	-4.45448017	2.86956191
O	-4.60753012	2.40143204	-2.34780788
N	-6.60757303	1.46720302	-1.85409796
O	-5.45567608	-4.34772205	2.56546092
C	-7.47465277	-1.47758496	0.24961799
H	-7.39949894	-3.21432900	1.55377996
C	-7.41550398	0.51666898	-1.22655702
H	-2.13798809	-5.11147881	2.23258805
H	-2.08394098	-3.96646690	3.59498405
H	-3.51193500	-5.04270601	3.38478899
C	-7.24461412	2.49954009	-2.65622807
H	-8.56122398	-1.46452796	0.15096000
O	-8.62333488	0.53826499	-1.34772301
H	-6.88409090	2.44809103	-3.69257808
H	-8.32497025	2.33357692	-2.62420988
H	-7.00116396	3.49259496	-2.25467396
Cl	4.33674717	0.93443400	0.08161000

Frequencies

Mode	IR frequency	IR intensity
1	9.31920000	0.00340000
2	9.97400000	0.01580000
3	11.64280000	0.11050000
4	15.25810000	0.32840000
5	17.17350000	0.32630000
6	25.93930000	0.57750000
7	35.75830000	0.05750000
8	37.77220000	0.01610000
9	39.01810000	0.04490000
10	40.31970000	0.02060000
11	40.60910000	0.03420000
12	44.33220000	0.20020000
13	50.83040000	0.03800000
14	52.78900000	0.16140000
15	54.05190000	0.19620000
16	57.93270000	0.10530000
17	60.07910000	0.41410000
18	65.84370000	0.28350000
19	65.99170000	0.66260000
20	70.20500000	0.61570000
21	73.51760000	0.32760000
22	76.10840000	0.79640000
23	76.89920000	0.64550000
24	79.42300000	1.77280000
25	81.10390000	0.04610000
26	82.63510000	1.80640000
27	89.72310000	3.51870000
28	94.70540000	4.93400000
29	101.48650000	4.30760000
30	107.62610000	2.63130000
31	113.53940000	1.42720000
32	114.94570000	1.40390000
33	118.06720000	0.95530000
34	130.87380000	0.95840000
35	133.59340000	3.61200000
36	135.52060000	5.49020000
37	136.48750000	10.09430000
38	139.37530000	2.36300000
39	142.97470000	2.25160000
40	148.15210000	1.07820000
41	153.05840000	2.00730000
42	159.56030000	1.57800000
43	164.15410000	1.94940000
44	166.90190000	2.30100000
45	169.35130000	1.34390000
46	171.16770000	0.69160000
47	176.63980000	3.96760000
48	179.43680000	2.16530000

49	185.83840000	0.24970000
50	197.43130000	0.25430000
51	211.99710000	15.38920000
52	216.41920000	0.34650000
53	221.68270000	1.29400000
54	222.84260000	1.32810000
55	224.16110000	0.89240000
56	233.75290000	0.01530000
57	234.80230000	0.09090000
58	256.41110000	0.00540000
59	257.24400000	4.44140000
60	257.73240000	0.12620000
61	257.92390000	0.00670000
62	258.67100000	0.46950000
63	258.75750000	0.08700000
64	259.11260000	0.42500000
65	273.64600000	0.30150000
66	294.87390000	48.13680000
67	299.15740000	0.52830000
68	302.03300000	1.74220000
69	322.75560000	0.11650000
70	334.78440000	3.78780000
71	335.53440000	0.43580000
72	339.41000000	0.88580000
73	340.87670000	9.95060000
74	343.28070000	21.05740000
75	362.66330000	7.38010000
76	371.88590000	41.55070000
77	376.71350000	7.57400000
78	383.93240000	4.00640000
79	389.37840000	28.24910000
80	404.03990000	0.18970000
81	409.26920000	0.09430000
82	409.79200000	94.70250000
83	411.15640000	23.13070000
84	414.41580000	10.82120000
85	420.48500000	4.12150000
86	421.64310000	0.56520000
87	457.37850000	17.31340000
88	461.65870000	0.16550000
89	476.34410000	6.88430000
90	490.82490000	20.69710000
91	505.77590000	93.43780000
92	521.58430000	33.99030000
93	525.39030000	33.22490000
94	561.37170000	20.49590000
95	576.60710000	3.37740000
96	588.98460000	8.64180000
97	597.31480000	16.33210000
98	613.36520000	4.36740000
99	635.38190000	0.76860000

100	685.23270000	1.24690000
101	690.56400000	30.18090000
102	691.91190000	2.88500000
103	701.05660000	2.79630000
104	704.53650000	4.22630000
105	722.18490000	23.07550000
106	723.53670000	30.26250000
107	723.97880000	11.04560000
108	726.72360000	17.47310000
109	744.18560000	16.26610000
110	749.67280000	0.01690000
111	756.37220000	0.08410000
112	756.66450000	0.13840000
113	762.13390000	7.50350000
114	762.97500000	2.33470000
115	764.27790000	7.84590000
116	764.94410000	3.81530000
117	768.41380000	6.59690000
118	769.90760000	20.70270000
119	787.85580000	1.54980000
120	789.20200000	3.87970000
121	791.09660000	14.81440000
122	792.46850000	42.72750000
123	793.83080000	21.64100000
124	796.01780000	76.16310000
125	813.92640000	43.20510000
126	858.70500000	0.03440000
127	869.01030000	4.26320000
128	884.51380000	4.89540000
129	884.60200000	0.74370000
130	903.99470000	8.34310000
131	904.59060000	10.13230000
132	904.81740000	25.81640000
133	905.93820000	25.26420000
134	906.16660000	10.90020000
135	914.94300000	7.86590000
136	915.23040000	8.76400000
137	915.45400000	10.36950000
138	915.64090000	16.99070000
139	915.99900000	9.00870000
140	916.12900000	2.41230000
141	931.63600000	1.70340000
142	971.86540000	14.76370000
143	976.24980000	0.29520000
144	1027.04690000	0.01430000
145	1031.44020000	88.41500000
146	1035.87890000	2.76580000
147	1036.29410000	0.88520000
148	1048.79660000	22.73770000
149	1051.42280000	2.74660000
150	1051.70730000	2.84080000

151	1052.01810000	3.74080000
152	1052.79730000	6.66820000
153	1053.44010000	8.39270000
154	1054.24840000	0.26530000
155	1066.73740000	102.46670000
156	1074.69650000	3.60150000
157	1075.37160000	4.31420000
158	1077.09820000	7.69940000
159	1078.59760000	10.33820000
160	1089.35240000	0.74910000
161	1089.56730000	0.72960000
162	1089.93530000	0.93160000
163	1090.26140000	4.13250000
164	1092.56660000	6.03990000
165	1092.99350000	0.97490000
166	1095.78720000	26.17000000
167	1116.48860000	15.43890000
168	1116.64460000	0.93530000
169	1117.35240000	26.56390000
170	1117.46770000	44.81230000
171	1120.92380000	128.28700000
172	1124.23830000	16.77310000
173	1140.67120000	0.96990000
174	1141.70350000	0.18300000
175	1163.09010000	97.30180000
176	1189.37010000	9.64090000
177	1205.22290000	172.91290000
178	1212.92010000	3.26020000
179	1214.47830000	1.60250000
180	1216.30520000	2.46560000
181	1216.56000000	2.26040000
182	1216.81580000	2.00250000
183	1217.80080000	4.07650000
184	1229.29410000	5.26650000
185	1230.02970000	16.42340000
186	1230.21260000	20.03940000
187	1230.80780000	13.55050000
188	1233.52770000	1.18760000
189	1248.79220000	38.55550000
190	1250.68050000	12.77990000
191	1253.08960000	43.74600000
192	1289.93430000	1.95000000
193	1290.86250000	2.12920000
194	1293.68290000	3.69640000
195	1295.83020000	2.05200000
196	1297.34860000	5.58350000
197	1298.07350000	4.97710000
198	1308.30900000	248.05970000
199	1317.93720000	6.61660000
200	1318.10010000	4.18710000
201	1318.36700000	2.62470000

202	1318.75340000	3.05860000
203	1319.37230000	1.26980000
204	1320.30200000	3.57920000
205	1320.80570000	1.33330000
206	1320.97680000	0.20230000
207	1322.13030000	6.96350000
208	1322.75470000	3.04040000
209	1326.29660000	23.72790000
210	1329.18210000	10.38240000
211	1334.79020000	318.46900000
212	1347.06410000	667.76650000
213	1370.92200000	128.35790000
214	1380.79240000	0.23380000
215	1381.01200000	0.21750000
216	1381.31050000	0.29990000
217	1382.08000000	0.15530000
218	1384.00600000	0.44110000
219	1384.56360000	0.20600000
220	1388.83070000	353.71350000
221	1398.32190000	0.28570000
222	1398.52290000	0.73090000
223	1398.79720000	0.66690000
224	1398.91150000	0.97250000
225	1401.08220000	16.34450000
226	1401.36930000	3.55800000
227	1403.95640000	32.39140000
228	1414.70850000	120.73550000
229	1415.62580000	72.69060000
230	1422.00820000	28.03020000
231	1422.50780000	3.39770000
232	1424.41860000	19.94490000
233	1426.55130000	17.14600000
234	1429.94220000	30.04970000
235	1442.74580000	145.59800000
236	1447.78900000	5.27010000
237	1447.89270000	0.79950000
238	1449.43710000	1.57280000
239	1449.57540000	1.82110000
240	1450.76270000	2.33200000
241	1450.79870000	3.01160000
242	1453.92810000	29.24450000
243	1456.11150000	18.35320000
244	1458.43410000	9.47970000
245	1458.48130000	8.66430000
246	1458.55730000	9.69380000
247	1458.61400000	9.64220000
248	1458.71840000	10.46130000
249	1458.89010000	10.10820000
250	1460.35880000	0.39190000
251	1460.48100000	0.51480000
252	1462.06490000	20.08700000

253	1465.18870000	0.77590000
254	1465.43400000	21.83640000
255	1465.50580000	29.36150000
256	1465.64170000	0.26900000
257	1465.80350000	22.82350000
258	1473.72160000	5.75060000
259	1473.75630000	5.12160000
260	1476.36730000	168.19720000
261	1476.83870000	16.06010000
262	1476.93920000	16.72760000
263	1477.61480000	22.39460000
264	1477.66760000	14.83300000
265	1491.40530000	300.08600000
266	1501.92750000	331.59780000
267	1521.03630000	275.68760000
268	1570.66000000	32.31280000
269	1638.58390000	463.08590000
270	1677.46340000	87.46170000
271	1699.57770000	162.22680000
272	1767.05770000	1091.00970000
273	1779.56250000	517.10010000
274	1809.35600000	655.16180000
275	1825.27470000	371.72790000
276	2184.02460000	2709.44290000
277	3044.37620000	28.24440000
278	3044.64560000	35.08830000
279	3044.73880000	41.08420000
280	3044.82600000	26.96220000
281	3045.00230000	32.80240000
282	3045.84520000	34.73860000
283	3047.77850000	21.74970000
284	3047.80770000	20.94200000
285	3048.12670000	18.65610000
286	3048.23080000	27.26820000
287	3048.24660000	46.30950000
288	3048.34580000	33.31070000
289	3056.66270000	38.58110000
290	3057.45210000	31.23020000
291	3057.67940000	0.74190000
292	3058.96590000	34.85860000
293	3060.01070000	42.64220000
294	3060.41490000	19.15140000
295	3064.23400000	44.03660000
296	3065.24230000	38.23030000
297	3065.54200000	39.86100000
298	3066.77170000	30.96690000
299	3067.41440000	36.42170000
300	3070.65650000	21.09410000
301	3085.06360000	28.46520000
302	3086.90320000	27.84140000
303	3089.58700000	10.54290000

304	3089.64160000	7.33070000
305	3089.66520000	11.96500000
306	3089.94460000	15.67780000
307	3090.04350000	7.71730000
308	3092.80410000	3.73090000
309	3114.94670000	7.05500000
310	3114.97960000	48.35870000
311	3115.57400000	7.75410000
312	3116.45950000	17.03190000
313	3116.83120000	23.97280000
314	3117.48920000	18.24300000
315	3130.38390000	15.83490000
316	3131.03120000	11.51840000
317	3132.12260000	16.67650000
318	3133.50180000	1.86530000
319	3133.91360000	6.38830000
320	3136.34320000	0.60640000
321	3137.21090000	55.93270000
322	3138.16450000	56.79070000
323	3138.24920000	50.33330000
324	3138.36770000	66.00790000
325	3138.41610000	59.01500000
326	3138.50110000	74.21820000
327	3145.61470000	34.00140000
328	3146.93980000	36.05630000
329	3146.97020000	34.27030000
330	3147.09460000	29.94310000
331	3147.20200000	36.27280000
332	3147.37340000	30.38730000
333	3177.61560000	8.56460000
334	3178.85210000	7.39570000
335	3233.88180000	2.17300000
336	3234.94960000	2.64220000
337	3237.45590000	1.33320000
338	3245.40330000	0.72680000
339	3248.04790000	0.79630000

2: Cl-Pt-CC-NDI in CH_2Cl_2 TRIPLET

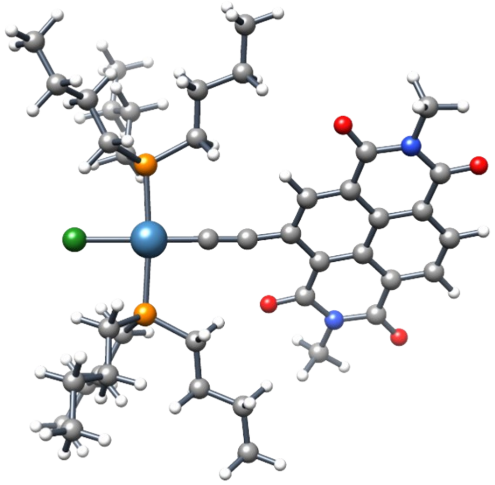

Optimised triplet ground state geometry of Cl-Pt-CC-NDI.

Route	:	#p opt freq genecp
	:	scrf=(cpcm,solvent=dichloromethane) geom=connectivity
	:	int=(acc2e=13,ultrafine) pbe1pbe
SMILES	:	CCCC[P](CCCC)(CCCC)[Pt]
	:	(C#Cc1cc2c3c(ccc4c3c1C(=O)N(C4=O)C)
	:	C(=O)N(C2=O)C)([P](CCCC)(CCCC)CCCC)Cl
Formula	:	$C_{42}H_{63}ClN_2O_4P_2Pt^3$
Charge	:	0
Multiplicity	:	3
Dipole	:	35.6730 Debye
Energy	:	-3306.77342362 a.u.
Gibbs Energy	:	-3305.90077700 a.u.
Number of imaginary frequencies	:	0

Cartesian Co-ordinates (XYZ format)

115

Pt	1.99737000	0.33224899	-0.06121000
P	1.67289603	2.65889096	0.24825400
P	2.53574896	-1.95172000	-0.38566601
C	2.61632609	3.56862712	-1.04215395
H	3.50081897	2.93824911	-1.22652805
H	1.99427795	3.50784898	-1.95247805
C	-0.08004000	3.20106697	0.16167700
H	-0.54788297	2.84203410	1.09501803
H	-0.52202600	2.58995104	-0.64081299

C	2.33521891	3.08483601	1.91077197
H	2.00555706	2.25652790	2.56062889
H	3.42799592	2.96657801	1.81535995
C	1.09311295	-3.04561210	-0.67500401
H	0.75403202	-2.81178594	-1.69987905
H	0.30203399	-2.67529607	-0.00196800
C	3.63385201	-2.03962398	-1.86053002
H	3.18597388	-1.33386898	-2.58075905
H	4.57808399	-1.56946099	-1.53844202
C	3.45116401	-2.54954696	1.09325504
H	4.01825094	-1.66810799	1.43341100
H	2.67727995	-2.73636508	1.85836399
C	3.87507296	-3.38198805	-2.54315996
H	2.91691995	-3.79650307	-2.89958811
H	4.28272486	-4.11575699	-1.82945502
C	4.82979918	-3.25441504	-3.72669792
H	5.78924894	-2.83861589	-3.37196803
H	4.42552280	-2.51292300	-4.43816614
C	5.07441711	-4.57366896	-4.44234514
H	5.76667690	-4.44992590	-5.28897810
H	5.51085281	-5.32291985	-3.76222491
H	4.13602114	-4.99445295	-4.83825779
C	1.26036501	-4.55058479	-0.48223600
H	2.09890199	-4.94169903	-1.08039200
H	1.50961006	-4.75859118	0.57238102
C	-0.00931100	-5.31336498	-0.84861702
H	-0.25262499	-5.11978483	-1.90821099
H	-0.85185498	-4.90300608	-0.26597199
C	0.10248500	-6.81124306	-0.61204100
H	-0.82490999	-7.33368921	-0.89213401
H	0.92305499	-7.25025988	-1.20242798
H	0.30305901	-7.03477812	0.44836599
C	4.39425707	-3.74221301	0.96162897
H	3.86114907	-4.62590313	0.57606798
H	5.18168688	-3.50884295	0.22501500
C	5.05186987	-4.09675407	2.29208994
H	5.57683897	-3.20747900	2.68309307
H	4.26592779	-4.33131599	3.03147292
C	6.02264595	-5.26252890	2.18706989
H	6.83954716	-5.04073715	1.48146296
H	6.47871304	-5.49540901	3.16129303
H	5.51652193	-6.17398405	1.83001602
C	1.96507895	4.41800785	2.55272698
H	0.87103999	4.47761822	2.68135095
H	2.24543810	5.25861120	1.89806199
C	2.63319612	4.60057116	3.91241503
H	2.35963511	3.75336695	4.56569719
H	3.72854400	4.54151583	3.78601289
C	2.26322103	5.91161489	4.58816910
H	2.55653691	6.77642488	3.97135997
H	1.17676103	5.98135281	4.75874901

H	2.76056600	6.01548195	5.56445408
C	-0.39616099	4.67364788	-0.08268300
H	0.09051500	5.31168795	0.67228699
H	0.01532000	4.98179483	-1.05888295
C	-1.89814496	4.94535589	-0.06934000
H	-2.39881706	4.29728699	-0.80976099
H	-2.30726409	4.64841795	0.91256100
C	-2.24236798	6.40017986	-0.34842500
H	-3.33025789	6.56568193	-0.32871899
H	-1.78934002	7.07108688	0.39942300
H	-1.87632895	6.71498299	-1.33909404
C	3.05868602	5.00452185	-0.77423197
H	3.71751904	5.02735281	0.11036600
H	2.19553995	5.64593506	-0.53448498
C	3.80729795	5.59963989	-1.96326697
H	4.66733408	4.95211077	-2.20873499
H	3.15056491	5.57685804	-2.85073590
C	4.28718996	7.02130222	-1.71618903
H	3.44343209	7.69694805	-1.50179303
H	4.97429609	7.06803513	-0.85592002
H	4.82115984	7.42332697	-2.59058690
C	0.13743900	-0.00015700	-0.08941600
C	-1.09258294	-0.22776200	-0.02380600
Cl	4.34363890	0.75368297	0.09998800
C	-2.48462796	-0.30687901	-0.04314700
C	-3.25201797	-1.39157796	0.56723398
C	-3.18254209	0.72403502	-0.70057303
C	-4.65829897	-1.39034200	0.44988200
C	-2.59945607	-2.46082497	1.32544005
C	-4.56426191	0.73108101	-0.81077898
H	-2.62297392	1.54101503	-1.15716600
C	-5.43952179	-2.42932701	1.01627803
C	-5.32897282	-0.33101201	-0.24623699
O	-1.38988400	-2.52214909	1.51170397
N	-3.41381097	-3.45812988	1.86122203
C	-5.23346710	1.82671595	-1.50894797
C	-4.80042410	-3.52447200	1.74988401
C	-6.83947706	-2.42299104	0.88623399
C	-6.72265720	-0.34324700	-0.35827801
C	-2.73263788	-4.50258303	2.60641098
O	-4.64055014	2.77545595	-2.00746703
N	-6.62390423	1.75638604	-1.59257698
O	-5.43412685	-4.44403791	2.24771690
C	-7.47359324	-1.39932501	0.21024300
H	-7.40210009	-3.24316311	1.33445597
C	-7.42103100	0.73806602	-1.06324995
H	-2.05876303	-5.06789684	1.94701803
H	-2.13121700	-4.06017208	3.41166091
H	-3.49492598	-5.16900587	3.01985693
C	-7.27552319	2.84977603	-2.29124594
H	-8.55919933	-1.37702203	0.10102900

O	-8.63705730	0.76060802	-1.18791401
H	-6.91419983	2.90703797	-3.32746792
H	-8.35330868	2.66439390	-2.27592993
H	-7.04640913	3.80481195	-1.79831398

Frequencies

Mode	IR frequency	IR intensity
1	5.45370000	0.19910000
2	9.45600000	0.05920000
3	10.29900000	0.12990000
4	15.49910000	0.34360000
5	18.08050000	0.27500000
6	24.56420000	0.28100000
7	34.96010000	0.60680000
8	38.63270000	0.03210000
9	39.32740000	0.16710000
10	39.92070000	0.01030000
11	40.72690000	0.03420000
12	43.15380000	0.85170000
13	50.51900000	0.05630000
14	52.92330000	0.05950000
15	55.65670000	0.10000000
16	60.01040000	0.11260000
17	61.22670000	0.55240000
18	65.37330000	0.32330000
19	66.37030000	0.38210000
20	69.18800000	0.24970000
21	72.68770000	0.22930000
22	76.01970000	0.22370000
23	78.58040000	0.48980000
24	79.45880000	0.31810000
25	89.22410000	3.32900000
26	91.28580000	0.17340000
27	92.70200000	4.95980000
28	97.87240000	3.39180000
29	103.25490000	2.10310000
30	111.52970000	3.82130000
31	112.87410000	1.39310000
32	116.20480000	3.13730000
33	120.50110000	1.23040000
34	130.11800000	0.15160000
35	134.50780000	2.22700000
36	135.38550000	3.15860000
37	138.83330000	5.48210000
38	139.23460000	17.61050000
39	142.53220000	2.96840000
40	148.65830000	1.66820000
41	153.65160000	1.90280000
42	159.83400000	1.86010000
43	162.84230000	1.69250000
44	164.98670000	4.29990000

45	167.76150000	1.35890000
46	171.53570000	4.19970000
47	176.04520000	2.84510000
48	177.00570000	3.28700000
49	186.31720000	6.11700000
50	196.78750000	4.71620000
51	210.92830000	1.14440000
52	218.13730000	0.00300000
53	219.16820000	2.19140000
54	223.30110000	1.97170000
55	223.93570000	0.99610000
56	232.89250000	0.67550000
57	233.95070000	0.78040000
58	246.05790000	3.35590000
59	256.69830000	0.11150000
60	257.05040000	0.14570000
61	257.44760000	0.02500000
62	257.98860000	0.16070000
63	258.28780000	0.14890000
64	258.44980000	0.17430000
65	281.57500000	1.48570000
66	296.98680000	35.57960000
67	301.34750000	3.58270000
68	307.01980000	206.36670000
69	311.49770000	7.60760000
70	335.05580000	32.06750000
71	337.03700000	0.18650000
72	338.66720000	11.62120000
73	342.32310000	12.39210000
74	343.03580000	22.70510000
75	353.58060000	24.93290000
76	371.61890000	6.86810000
77	377.22270000	4.53930000
78	382.56610000	37.01880000
79	386.75820000	38.28020000
80	403.76410000	0.09290000
81	408.86730000	0.47830000
82	409.84990000	58.38020000
83	410.91890000	49.02560000
84	413.31660000	1.85840000
85	421.08980000	14.91390000
86	422.51040000	55.46910000
87	433.63720000	1.41770000
88	464.90190000	92.42170000
89	480.84510000	10.37410000
90	495.67480000	33.70140000
91	508.81420000	84.49110000
92	520.81390000	218.08640000
93	523.76930000	61.62090000
94	557.85280000	2.23810000
95	564.58570000	4.43380000

96	585.48550000	49.15810000
97	591.02330000	11.57300000
98	609.95120000	14.08030000
99	629.77500000	59.86210000
100	655.01990000	5.96330000
101	690.86950000	29.04560000
102	692.25170000	14.56440000
103	703.20380000	4.43410000
104	706.11740000	10.50830000
105	722.52690000	24.40400000
106	723.70250000	31.30500000
107	724.02200000	6.83370000
108	728.74320000	18.31300000
109	741.49650000	4.72270000
110	743.09300000	6.30680000
111	755.97750000	0.36160000
112	755.99870000	2.34760000
113	756.84880000	20.81310000
114	762.57830000	5.59820000
115	763.30020000	0.91980000
116	763.90670000	4.36050000
117	765.01200000	4.03990000
118	767.98240000	0.13330000
119	778.20550000	2.39930000
120	786.61970000	68.68910000
121	790.28840000	1.56750000
122	793.55140000	6.70110000
123	794.25580000	3.14220000
124	794.85170000	56.90600000
125	798.03750000	53.36640000
126	849.95930000	3.65330000
127	863.68670000	72.49090000
128	872.35080000	6.22330000
129	884.93500000	5.03860000
130	885.08490000	3.41030000
131	904.48810000	1.78320000
132	905.00850000	19.10470000
133	905.18670000	14.61790000
134	906.30790000	20.69770000
135	915.31830000	10.97790000
136	915.75760000	19.05180000
137	915.80500000	9.64630000
138	916.10240000	8.22510000
139	916.50860000	5.35760000
140	917.29370000	12.03470000
141	922.01810000	118.43140000
142	948.47750000	14.26980000
143	968.76480000	34.21920000
144	1015.10930000	0.10270000
145	1029.20730000	39.80150000
146	1037.79010000	3.68990000

147	1038.63050000	5.00880000
148	1044.57260000	58.37990000
149	1051.71640000	0.96630000
150	1052.07250000	7.74920000
151	1052.46580000	6.25720000
152	1053.16260000	7.20990000
153	1053.74450000	4.52030000
154	1054.32900000	1.43090000
155	1062.18370000	67.26280000
156	1075.74830000	4.34310000
157	1077.02560000	3.10360000
158	1078.56040000	5.89060000
159	1080.43910000	2.59930000
160	1087.81010000	17.87050000
161	1089.33040000	0.60720000
162	1089.73390000	1.20050000
163	1089.96570000	0.50070000
164	1090.19100000	1.31960000
165	1092.45900000	7.67000000
166	1092.84220000	0.73030000
167	1116.12190000	11.69710000
168	1116.63480000	5.75320000
169	1117.23930000	21.05310000
170	1117.40870000	47.78660000
171	1120.69730000	132.60770000
172	1123.41460000	130.89280000
173	1140.47800000	0.34350000
174	1141.66680000	0.22960000
175	1157.70880000	6.73950000
176	1180.34440000	444.82120000
177	1212.22450000	23.77780000
178	1213.55650000	0.89080000
179	1215.04160000	0.92270000
180	1217.04870000	0.58890000
181	1217.13000000	5.62620000
182	1217.65020000	1.89660000
183	1218.36750000	0.34430000
184	1227.07010000	34.94620000
185	1230.18020000	1.21730000
186	1230.35540000	1.93950000
187	1232.00690000	2.03230000
188	1235.20690000	3.00060000
189	1250.08660000	77.61770000
190	1251.86330000	77.21630000
191	1256.02820000	57.38050000
192	1273.15810000	1235.19580000
193	1290.87700000	0.92680000
194	1291.49860000	0.91960000
195	1294.89180000	2.78820000
196	1296.91330000	2.11280000
197	1297.78990000	3.85630000

198	1299.71010000	1.96650000
199	1316.11700000	299.01770000
200	1317.92270000	3.11830000
201	1318.95510000	4.56100000
202	1319.35960000	4.79960000
203	1319.51590000	3.70490000
204	1319.66160000	0.05890000
205	1320.02870000	3.49710000
206	1320.78660000	1.42490000
207	1321.74140000	0.67370000
208	1322.85920000	15.98100000
209	1323.37580000	33.31870000
210	1326.56580000	252.39580000
211	1327.89200000	187.15040000
212	1330.60150000	13.40310000
213	1366.52030000	302.16500000
214	1377.45270000	1383.53590000
215	1381.19490000	0.23900000
216	1381.31090000	0.11580000
217	1381.81610000	0.38880000
218	1381.97280000	0.18840000
219	1384.53170000	0.25710000
220	1384.79340000	1.46250000
221	1398.68250000	0.15840000
222	1398.72700000	0.11480000
223	1398.81930000	1.73640000
224	1399.28520000	0.79460000
225	1400.93130000	295.87320000
226	1401.45200000	27.55520000
227	1401.84220000	21.47290000
228	1403.26780000	292.44400000
229	1417.39350000	68.38950000
230	1420.68540000	46.56340000
231	1420.99510000	190.02190000
232	1421.60590000	384.30920000
233	1423.06160000	13.19170000
234	1425.65080000	24.27840000
235	1431.89040000	16.17160000
236	1447.65210000	5.13170000
237	1447.76470000	7.89090000
238	1449.39950000	1.64900000
239	1449.57390000	0.98570000
240	1449.78630000	10.94250000
241	1451.02290000	2.15990000
242	1452.14860000	226.77360000
243	1454.04330000	21.99070000
244	1456.04580000	14.84840000
245	1458.19200000	10.32830000
246	1458.67520000	8.21510000
247	1458.69990000	12.67180000
248	1458.71820000	7.07390000

249	1458.85020000	11.93030000
250	1458.96360000	9.51980000
251	1459.27290000	31.64900000
252	1460.21980000	1.16980000
253	1460.38330000	0.26030000
254	1465.14890000	0.16300000
255	1465.31900000	0.09950000
256	1465.51030000	0.03600000
257	1465.53330000	0.13990000
258	1466.16050000	4.03260000
259	1473.37990000	3.93530000
260	1473.70550000	7.73760000
261	1476.78530000	15.38460000
262	1476.88470000	15.71170000
263	1477.31380000	14.98470000
264	1477.59950000	18.23320000
265	1482.87980000	24.59150000
266	1493.65890000	62.32340000
267	1513.68250000	278.33230000
268	1542.03250000	366.45020000
269	1571.45750000	2242.23630000
270	1601.55270000	1242.10480000
271	1657.20330000	378.05670000
272	1714.55720000	1169.46540000
273	1734.58470000	1507.90290000
274	1760.78530000	1137.87450000
275	1778.39910000	979.83170000
276	2104.27420000	75.47070000
277	3045.46390000	33.61890000
278	3045.53820000	43.06610000
279	3045.65610000	26.42200000
280	3045.71020000	33.11180000
281	3046.19550000	25.91320000
282	3046.80840000	34.82690000
283	3048.06890000	24.42890000
284	3048.73470000	13.73880000
285	3048.80440000	45.69890000
286	3048.91580000	12.38060000
287	3048.95810000	47.77060000
288	3049.00290000	19.16440000
289	3055.25750000	45.76670000
290	3059.22970000	26.90510000
291	3059.55050000	14.25090000
292	3060.57080000	23.96350000
293	3061.43080000	50.92780000
294	3061.51100000	12.42040000
295	3063.72550000	47.03930000
296	3066.79230000	38.61520000
297	3067.21100000	26.52210000
298	3067.72770000	28.83370000
299	3068.92270000	26.69550000

300	3070.59490000	26.48760000
301	3081.82370000	48.81360000
302	3083.12870000	27.48930000
303	3090.67930000	11.87390000
304	3090.73210000	9.35850000
305	3090.77800000	12.65390000
306	3090.92410000	9.59360000
307	3092.11130000	5.09680000
308	3094.61230000	4.23700000
309	3115.64530000	55.14940000
310	3116.18400000	7.20420000
311	3117.47520000	10.10840000
312	3118.28800000	16.69440000
313	3118.47130000	14.39610000
314	3118.71580000	16.48920000
315	3130.73020000	7.15250000
316	3131.57440000	14.59920000
317	3132.98460000	11.43620000
318	3134.66380000	4.00300000
319	3136.14160000	3.91150000
320	3136.79920000	1.51310000
321	3138.01630000	56.17760000
322	3138.59060000	59.79970000
323	3139.07640000	49.83250000
324	3139.16040000	66.12620000
325	3139.25530000	56.96400000
326	3139.41620000	66.35600000
327	3147.02150000	30.46600000
328	3147.99360000	36.88610000
329	3148.01080000	31.54070000
330	3148.06460000	27.38040000
331	3148.14480000	34.06680000
332	3148.36480000	30.67560000
333	3170.13720000	11.23630000
334	3173.27260000	10.40830000
335	3230.76820000	2.69350000
336	3231.73670000	2.21750000
337	3235.46680000	4.94300000
338	3243.69790000	3.50230000
339	3247.92750000	6.53390000

3: Ph-CC-Pt-CC-NDI in CH_2Cl_2, SINGLET

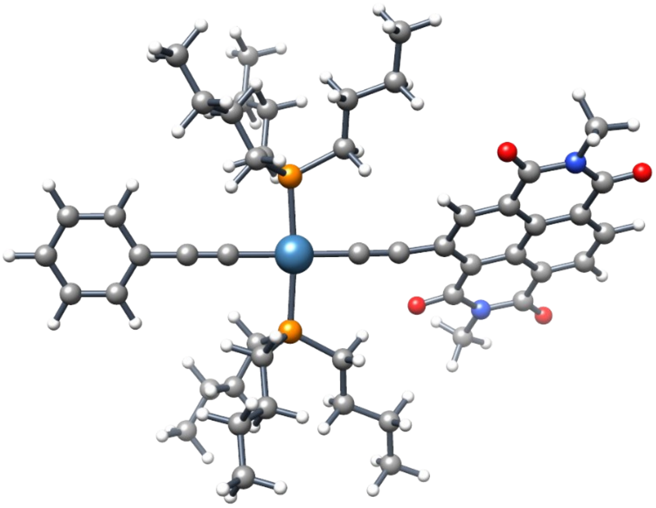

Optimised singlet ground state geometry of Ph-CC-Pt-CC-NDI.

Route	:	#p opt freq PBE1PBE/genecp
	:	scrf=(cpcm,solvent=dichloromethane) geom=connectivity
	:	int=(acc2e=13,ultrafine)
SMILES	:	CCCC[P](CCCC)(CCCC)[Pt]
	:	(C#Cc1ccccc1)(C#Cc2cc3c4c(ccc5c4c2C(=O)N(C5=O)C)
	:	C(=O)N(C3=O)C)[P](CCCC)(CCCC)CCCC
Formula	:	$C_{50}H_{68}N_2O_4P_2Pt$
Charge	:	0
Multiplicity	:	1
Dipole	:	12.5326 Debye
Energy	:	-3154.10664104 a.u.
Gibbs Energy	:	-3153.13992300 a.u.
Number of imaginary frequencies	:	0

Cartesian Co-ordinates (XYZ format)

127

Pt	1.44295299	0.07700500	-0.08976700
C	3.43394804	0.31483901	-0.03172800
P	1.27538502	2.39403701	0.25383800
P	1.85283196	-2.20793009	-0.42944100
C	4.65732002	0.45934501	0.01820400
C	6.07598877	0.62532300	0.07376200
C	6.93309116	-0.49048200	0.16729701
C	6.65789223	1.90920401	0.03634800
C	8.31428719	-0.32573599	0.22070500

H	6.49825716	-1.49245405	0.19768500
C	8.03989124	2.06709099	0.08986900
H	6.00784492	2.78452992	-0.03585100
C	8.87519455	0.95221299	0.18236400
H	8.96026230	-1.20453894	0.29322800
H	8.47000122	3.07153201	0.05922900
C	2.26625395	3.30123806	-1.00735402
H	3.10158205	2.62142992	-1.23797405
H	1.63295496	3.32903695	-1.91163898
C	-0.43975499	3.05615211	0.18134899
H	-0.92667103	2.71877289	1.11279297
H	-0.92347300	2.47575307	-0.62016302
C	1.95827103	2.76474595	1.92487597
H	1.60934305	1.93031597	2.55633497
H	3.04769897	2.61992407	1.82472801
C	0.34943399	-3.25355911	-0.57702303
H	-0.05676000	-3.04216194	-1.58205295
H	-0.37721401	-2.81698799	0.12724800
C	2.81830406	-2.38145089	-1.99021494
H	2.37021089	-1.64192903	-2.67523789
H	3.82072902	-1.98729897	-1.75105095
C	2.86156607	-2.86941791	0.96409303
H	3.47886395	-2.01471400	1.28292799
H	2.14384389	-3.05338097	1.78290606
C	2.89968395	-3.73615694	-2.68540812
H	1.88506496	-4.08247805	-2.94610596
H	3.32068801	-4.49774408	-2.00946307
C	3.74264693	-3.67733788	-3.95607305
H	4.75918484	-3.33121610	-3.69856811
H	3.32575798	-2.90844297	-4.63029003
C	3.82290697	-5.01008320	-4.68410683
H	4.43762112	-4.93602991	-5.59412003
H	4.26785517	-5.78936815	-4.04437304
H	2.82309008	-5.36233521	-4.98546410
C	0.46252799	-4.75619984	-0.33787000
H	1.19207001	-5.21551704	-1.02453995
H	0.84207797	-4.94014502	0.68191302
C	-0.88243997	-5.46022606	-0.49310499
H	-1.26956904	-5.27853394	-1.51137996
H	-1.60881603	-4.99254704	0.19378200
C	-0.80978101	-6.95650196	-0.23146400
H	-1.79362297	-7.43554211	-0.35117701
H	-0.11213000	-7.45174789	-0.92630303
H	-0.45972699	-7.16721582	0.79217201
C	3.75517201	-4.08489418	0.73663402
H	3.16584396	-4.94873095	0.38938701
H	4.48072290	-3.86337399	-0.06480400
C	4.51947117	-4.47668982	1.99785101
H	5.10759783	-3.60932803	2.34640503
H	3.79741001	-4.69403982	2.80465794
C	5.43751383	-5.67199707	1.79486597

H	6.19269323	-5.46915007	1.01826096
H	5.97381592	-5.93033314	2.72068095
H	4.87085676	-6.56280422	1.47883296
C	1.63056302	4.09026098	2.60371304
H	0.53807998	4.18560791	2.72489095
H	1.94639003	4.93907309	1.97621405
C	2.29138708	4.21011496	3.97391391
H	1.98179996	3.35453200	4.59972000
H	3.38526607	4.11545706	3.85600209
C	1.96243298	5.51308918	4.68573523
H	2.29241204	6.38476992	4.09769392
H	0.87753099	5.61714888	4.84899712
H	2.45363903	5.57023716	5.66904116
C	-0.66665798	4.54622221	-0.05100000
H	-0.16766900	5.14711285	0.72635001
H	-0.21184801	4.84555006	-1.01068699
C	-2.15090394	4.90180683	-0.07731700
H	-2.65835404	4.29801178	-0.85030103
H	-2.60879207	4.60517216	0.88292801
C	-2.40708089	6.37908316	-0.33230501
H	-3.48431301	6.60471392	-0.34431401
H	-1.94395804	7.00665712	0.44635001
H	-1.99022806	6.69575977	-1.30209899
C	2.81265903	4.68752813	-0.67999798
H	3.46395111	4.62601423	0.20871601
H	1.99789298	5.38170004	-0.41820800
C	3.61500692	5.27387094	-1.83793402
H	4.42998314	4.57634878	-2.10062408
H	2.96918011	5.33202982	-2.73183393
C	4.19244814	6.64696217	-1.53151095
H	3.39673305	7.37286282	-1.29822898
H	4.87073278	6.61121416	-0.66363698
H	4.76438904	7.04325914	-2.38432789
C	-0.52675301	-0.15387399	-0.09782800
C	-1.75374305	-0.30068299	-0.04617800
C	-3.16634798	-0.26947001	-0.07635300
C	-4.03215313	-1.15006304	0.60494000
C	-3.75395107	0.77284098	-0.86181003
C	-5.43906689	-0.98018003	0.50889403
C	-3.50734401	-2.25861597	1.42826998
C	-5.11131716	0.94411200	-0.95722502
H	-3.09877110	1.45888805	-1.40001297
C	-6.33231401	-1.84176803	1.18850899
C	-5.98813295	0.06729100	-0.27426201
O	-2.32237291	-2.50136709	1.55817902
N	-4.43526888	-3.07182002	2.08896589
C	-5.65711880	2.05643511	-1.77253902
C	-5.81699896	-2.94709110	2.02564406
C	-7.70483923	-1.66413796	1.08412504
C	-7.38335800	0.23249300	-0.36936501
C	-3.87753391	-4.14725399	2.89255810

O	-4.94884014	2.84886503	-2.36183691
N	-7.04168177	2.16798401	-1.83946502
O	-6.55545521	-3.70588589	2.62524390
C	-8.23616219	-0.62907600	0.30502999
H	-8.35805798	-2.35126090	1.62484598
C	-7.95177984	1.32936895	-1.19270504
H	-3.36023998	-4.87538099	2.25148201
H	-3.15095401	-3.74209499	3.60852504
H	-4.70228195	-4.63612413	3.41867900
C	-7.55746508	3.26164794	-2.64740801
H	-9.31409836	-0.48428699	0.21751601
O	-9.14941788	1.49673796	-1.30143404
H	-7.20952797	3.16080403	-3.68436909
H	-8.64966488	3.22352695	-2.61035991
H	-7.19830608	4.22267389	-2.25468206
H	9.95973015	1.07887304	0.22444300

Frequencies

Mode	IR frequency	IR intensity
1	7.39180000	0.00900000
2	8.72290000	0.09350000
3	10.30850000	0.08480000
4	12.10600000	0.04860000
5	13.03070000	0.01090000
6	16.13530000	0.07110000
7	18.80660000	0.06760000
8	20.20490000	0.34660000
9	25.39480000	0.67010000
10	34.46910000	0.17260000
11	39.15800000	0.02090000
12	39.46160000	0.00900000
13	40.19130000	0.02570000
14	40.84020000	0.14470000
15	46.11170000	0.23550000
16	50.85550000	0.05510000
17	53.61730000	0.14580000
18	55.51250000	0.10070000
19	61.49750000	0.14830000
20	63.76990000	0.16760000
21	67.07480000	0.35460000
22	68.74240000	0.21240000
23	70.70910000	0.05660000
24	72.51990000	0.77950000
25	78.35220000	0.24050000
26	78.66630000	0.32870000
27	81.88240000	0.04040000
28	85.61150000	3.90070000
29	88.60960000	1.71730000
30	89.94120000	4.01310000
31	96.91800000	2.42360000
32	102.47540000	8.33360000

33	106.96800000	1.62460000
34	109.25940000	2.46010000
35	114.21950000	0.15580000
36	115.54850000	0.21610000
37	119.51930000	3.81170000
38	122.72510000	4.19810000
39	136.21870000	0.12860000
40	137.50290000	12.41340000
41	139.81880000	1.90330000
42	143.02240000	0.31550000
43	147.93830000	1.46310000
44	151.53010000	1.69080000
45	161.42400000	1.13460000
46	163.45850000	0.47760000
47	165.48170000	0.73920000
48	169.09470000	0.56590000
49	170.74830000	0.84600000
50	172.44280000	0.45950000
51	176.70680000	0.48160000
52	178.53150000	1.07370000
53	197.82760000	0.29670000
54	203.29360000	2.83600000
55	217.72430000	1.31110000
56	219.04150000	0.30140000
57	221.39960000	2.95350000
58	223.73520000	2.70450000
59	225.98640000	3.06150000
60	227.17260000	1.12720000
61	233.73140000	0.17540000
62	234.81250000	0.12120000
63	257.16320000	0.06660000
64	257.66050000	0.04750000
65	258.18890000	0.04560000
66	258.34370000	0.05910000
67	258.50110000	0.17380000
68	259.33400000	0.15450000
69	272.63530000	3.11240000
70	274.62570000	2.01740000
71	298.51310000	1.83360000
72	300.13750000	1.76810000
73	323.28930000	0.23960000
74	330.12450000	3.61850000
75	337.52560000	0.63110000
76	339.13000000	0.45990000
77	342.15840000	22.93430000
78	343.23110000	13.10430000
79	357.51670000	1.64200000
80	370.32080000	36.04430000
81	376.44440000	22.90560000
82	378.55030000	12.63610000
83	384.62370000	1.54440000

84	390.65910000	15.23140000
85	404.60480000	0.32000000
86	408.13070000	32.00440000
87	410.69560000	10.60120000
88	411.87860000	67.37150000
89	414.00020000	16.41870000
90	414.78370000	9.58950000
91	417.64840000	0.00590000
92	421.25370000	2.81300000
93	443.43070000	5.02550000
94	453.14300000	15.62290000
95	462.26840000	0.07380000
96	484.75790000	7.31750000
97	493.16360000	21.06700000
98	505.72240000	99.91000000
99	521.32070000	24.07380000
100	525.74320000	11.01230000
101	542.83700000	23.92820000
102	550.30830000	8.91260000
103	568.00410000	1.10130000
104	570.13810000	8.80660000
105	579.11150000	3.11550000
106	588.11880000	4.20970000
107	597.90010000	16.38060000
108	613.11000000	1.25020000
109	632.49620000	0.04950000
110	635.34690000	1.17310000
111	686.77150000	1.16810000
112	692.16180000	21.33540000
113	693.34400000	6.04030000
114	701.76720000	3.32960000
115	704.00700000	3.45300000
116	717.80150000	48.78210000
117	721.27100000	21.01350000
118	721.94660000	28.01830000
119	722.93870000	16.42300000
120	726.77990000	18.80990000
121	742.28110000	8.19050000
122	750.22050000	0.01230000
123	756.41660000	0.20230000
124	756.59870000	0.23180000
125	761.97120000	6.36510000
126	762.58040000	3.88710000
127	763.13790000	5.66350000
128	764.39810000	7.08610000
129	766.29500000	15.17260000
130	770.40470000	22.40770000
131	787.09190000	5.94350000
132	787.37920000	3.37660000
133	789.97090000	13.19530000
134	791.36170000	22.61620000

135	792.04240000	67.62140000
136	794.19700000	81.21420000
137	794.35250000	32.79000000
138	814.62600000	42.89820000
139	818.31420000	7.51920000
140	859.26950000	0.02570000
141	864.74360000	0.00400000
142	869.12800000	4.44190000
143	884.64910000	4.08870000
144	884.84710000	1.45240000
145	904.22500000	8.33310000
146	904.64670000	7.73570000
147	904.96040000	29.82770000
148	905.79240000	28.65930000
149	906.21440000	10.68010000
150	914.78000000	1.30810000
151	914.89820000	19.22340000
152	915.12640000	9.57900000
153	915.34150000	7.74240000
154	915.78610000	12.21220000
155	916.16860000	2.69150000
156	929.22500000	5.47530000
157	946.44410000	6.85460000
158	971.33000000	14.03510000
159	976.08070000	0.25140000
160	1007.84120000	0.00040000
161	1010.86410000	0.00810000
162	1026.89330000	0.01110000
163	1028.72200000	0.19870000
164	1030.45410000	96.35790000
165	1035.99210000	3.92430000
166	1036.17970000	0.66920000
167	1047.69440000	28.43400000
168	1050.92480000	5.92530000
169	1051.41250000	1.54890000
170	1051.59870000	4.42310000
171	1052.37010000	4.17200000
172	1053.02670000	9.41270000
173	1053.99200000	1.22860000
174	1056.42660000	12.80620000
175	1066.49870000	105.57200000
176	1074.06590000	5.71630000
177	1074.24840000	4.45700000
178	1076.42550000	8.46140000
179	1076.93620000	13.70350000
180	1088.76050000	0.69720000
181	1089.44320000	1.45000000
182	1089.65390000	2.45380000
183	1090.30870000	3.42440000
184	1092.05180000	5.74040000
185	1092.65530000	1.30250000

186	1094.98920000	24.98460000
187	1098.78000000	12.61730000
188	1116.20360000	11.77290000
189	1116.36500000	5.28180000
190	1117.01490000	42.20730000
191	1117.10990000	22.90300000
192	1120.69700000	118.79130000
193	1123.76390000	5.51660000
194	1141.43310000	0.79130000
195	1142.21710000	0.19230000
196	1162.08720000	88.82560000
197	1162.85000000	0.00290000
198	1181.79030000	0.92550000
199	1189.60160000	9.49910000
200	1205.63430000	204.39570000
201	1213.52520000	2.07680000
202	1214.29870000	3.47410000
203	1216.46890000	2.01640000
204	1216.59650000	2.52360000
205	1217.03840000	1.77370000
206	1217.26470000	6.44390000
207	1228.47910000	12.71480000
208	1228.60870000	2.43490000
209	1229.03290000	22.38420000
210	1230.17490000	4.77500000
211	1232.57130000	0.52600000
212	1247.86520000	28.44580000
213	1249.43020000	11.89010000
214	1253.06770000	38.11720000
215	1256.89100000	47.75620000
216	1289.91160000	3.60200000
217	1290.78640000	2.16370000
218	1292.96420000	1.76650000
219	1295.49040000	1.61210000
220	1296.98030000	5.69120000
221	1297.49090000	9.01620000
222	1306.22490000	290.14230000
223	1315.46020000	0.11800000
224	1317.89300000	2.78630000
225	1318.14180000	2.03210000
226	1318.58390000	3.51550000
227	1318.72670000	2.65710000
228	1319.14570000	0.68930000
229	1319.54540000	0.98910000
230	1321.15410000	2.09940000
231	1321.27100000	1.29310000
232	1322.02390000	0.71870000
233	1322.11870000	5.09150000
234	1325.66680000	19.35130000
235	1329.13010000	12.14780000
236	1334.54750000	330.05100000

237	1347.04300000	699.82610000
238	1370.47420000	119.86410000
239	1380.15440000	0.02910000
240	1380.82460000	0.14490000
241	1381.21000000	0.17840000
242	1381.57210000	0.23160000
243	1381.65400000	0.31640000
244	1383.87620000	0.63720000
245	1384.33290000	0.11240000
246	1388.61620000	366.17760000
247	1398.38210000	0.45240000
248	1398.45820000	1.32370000
249	1398.71130000	0.61240000
250	1398.79590000	2.59120000
251	1400.64660000	15.83650000
252	1401.21790000	5.06760000
253	1403.95630000	30.90410000
254	1414.37060000	78.42710000
255	1415.28250000	114.55570000
256	1420.86510000	14.26530000
257	1421.58900000	16.29890000
258	1423.02620000	14.58320000
259	1425.42480000	14.82470000
260	1429.33900000	33.32390000
261	1442.95740000	158.83860000
262	1447.45500000	3.88510000
263	1447.54440000	0.49400000
264	1449.12960000	1.57380000
265	1449.31810000	1.48110000
266	1450.61330000	1.88960000
267	1451.03870000	2.75800000
268	1454.42820000	28.74930000
269	1456.70490000	18.12800000
270	1458.30990000	8.75640000
271	1458.49940000	9.08880000
272	1458.59100000	10.29420000
273	1458.66860000	9.38960000
274	1458.72770000	9.30510000
275	1458.89720000	10.44370000
276	1460.19420000	0.65390000
277	1460.44020000	0.63020000
278	1461.76000000	22.11580000
279	1465.38210000	0.83510000
280	1465.42320000	0.80120000
281	1465.66140000	32.01050000
282	1465.75240000	0.17980000
283	1465.90900000	45.43900000
284	1473.48830000	4.87860000
285	1473.60340000	6.05170000
286	1476.73800000	142.28830000
287	1476.79640000	53.18990000

288	1477.07280000	18.14330000
289	1477.72040000	12.14650000
290	1477.80790000	24.00320000
291	1478.48130000	4.91410000
292	1490.44540000	355.74070000
293	1501.84510000	342.18480000
294	1520.87850000	279.13410000
295	1526.94910000	68.70780000
296	1570.52490000	31.36570000
297	1638.52130000	511.12610000
298	1643.01530000	3.62860000
299	1677.32000000	102.89000000
300	1678.81600000	68.28620000
301	1699.52030000	173.93190000
302	1767.32670000	1094.22320000
303	1779.75770000	507.85070000
304	1809.35350000	659.95000000
305	1825.37940000	391.67370000
306	2172.06000000	2986.98290000
307	2210.39710000	32.43610000
308	3043.93460000	40.73310000
309	3043.97960000	25.25740000
310	3044.50140000	35.14670000
311	3044.78460000	30.02150000
312	3044.90850000	33.08180000
313	3045.73950000	34.84180000
314	3047.65850000	20.25750000
315	3047.69690000	22.27390000
316	3047.97440000	21.79570000
317	3048.04960000	27.85020000
318	3048.13760000	42.76760000
319	3048.31010000	31.37090000
320	3057.07420000	32.69410000
321	3057.16400000	24.59380000
322	3057.60460000	30.29380000
323	3059.67090000	32.57860000
324	3059.81960000	27.91370000
325	3061.14780000	36.19830000
326	3064.94210000	47.42270000
327	3065.18080000	34.05780000
328	3065.59580000	20.91360000
329	3067.12630000	32.47210000
330	3067.20960000	28.56430000
331	3071.36330000	24.54570000
332	3085.15140000	28.23590000
333	3087.12720000	28.48300000
334	3088.93180000	11.74240000
335	3089.09800000	12.36730000
336	3089.38500000	11.43090000
337	3089.84720000	11.58650000
338	3090.17980000	7.00480000

339	3093.16750000	3.62520000
340	3114.65650000	7.00710000
341	3114.77270000	8.57800000
342	3115.64530000	41.78620000
343	3116.29630000	22.25930000
344	3116.72380000	21.81820000
345	3117.87580000	13.93600000
346	3130.34370000	16.64910000
347	3130.46370000	15.54370000
348	3133.66160000	11.71080000
349	3133.95000000	4.76190000
350	3134.27770000	3.58060000
351	3135.30910000	1.68520000
352	3137.12230000	55.64620000
353	3138.05940000	60.85980000
354	3138.09190000	56.90530000
355	3138.21500000	30.54600000
356	3138.22590000	94.13430000
357	3138.39570000	63.52870000
358	3145.24300000	34.45140000
359	3146.87740000	34.97560000
360	3146.98380000	37.77880000
361	3147.05440000	26.17600000
362	3147.11260000	36.56350000
363	3147.27890000	30.26000000
364	3177.68530000	8.65740000
365	3178.79420000	7.56980000
366	3201.08620000	7.93360000
367	3206.50650000	0.17120000
368	3215.56040000	5.52730000
369	3220.16870000	26.20350000
370	3228.28190000	19.06370000
371	3233.22380000	2.07780000
372	3235.60410000	2.60890000
373	3237.36600000	1.38810000
374	3245.75160000	0.86540000
375	3248.07850000	0.71270000

4: Ph-CC-Pt-CC-NDI in CH_2Cl_2, TRIPLET

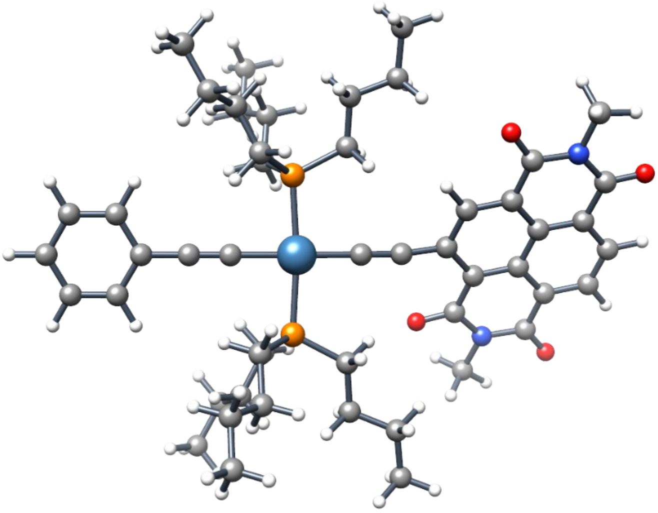

Optimised triplet ground state geometry of Ph-CC-Pt-CC-NDI.

Route	:	#p opt freq genecp
	:	scrf=(cpcm,solvent=dichloromethane) geom=connectivity
	:	int=(acc2e=13,ultrafine) pbe1pbe
SMILES	:	CCCC[P](CCCC)(CCCC)[Pt]
	:	(C#Cc1ccccc1)(C#Cc2cc3c4c(ccc5c4c2C(=O)N(C5=O)C)
	:	C(=O)N(C3=O)C)[P](CCCC)(CCCC)CCCC
Formula	:	$C_{50}H_{68}N_2O_4P_2Pt_3$
Charge	:	0
Multiplicity	:	3
Dipole	:	55.9001 Debye
Energy	:	-3154.04923528 a.u.
Gibbs Energy	:	-3153.08501300 a.u.
Number of imaginary frequencies	:	0

Cartesian Co-ordinates (XYZ format)

127

Pt	1.43814504	0.08752200	-0.12758800
C	3.41612291	0.26642400	-0.03616200
P	1.33542597	2.43080902	0.17617400
P	1.78268898	-2.22886109	-0.42919999
C	4.64448309	0.37836501	0.05322400
C	6.05877495	0.50534499	0.14955500
C	6.87561703	-0.63515800	0.30435500
C	6.67431116	1.77414203	0.09217300
C	8.25692272	-0.50694001	0.39812300

H	6.40810585	-1.62133396	0.35035300
C	8.05643272	1.89325798	0.18607201
H	6.05035019	2.66275501	-0.02747300
C	8.85219955	0.75542903	0.33939600
H	8.87674999	-1.39873803	0.51825798
H	8.51923180	2.88199306	0.13974901
C	2.34109092	3.26679111	-1.11974597
H	3.15764809	2.56030607	-1.33642304
H	1.70136404	3.28155208	-2.01967907
C	-0.36243701	3.13009405	0.10298700
H	-0.85158300	2.82008505	1.04280400
H	-0.86584198	2.55455303	-0.68957901
C	2.04537392	2.80819392	1.83214903
H	1.67312801	2.00037408	2.48453689
H	3.12863493	2.62600207	1.72690701
C	0.25238901	-3.18777895	-0.74822700
H	-0.03458800	-2.93930697	-1.78542101
H	-0.51783299	-2.73206496	-0.10415700
C	2.91096807	-2.45385003	-1.86703706
H	2.57505798	-1.70472097	-2.60382295
H	3.89589810	-2.10016298	-1.51837206
C	2.58917308	-2.90605211	1.08077502
H	3.21825290	-2.08225989	1.45400596
H	1.77361596	-3.02448893	1.81569600
C	3.01479101	-3.81972790	-2.53752804
H	2.02627301	-4.12304401	-2.92196107
H	3.31267500	-4.59095192	-1.80905700
C	4.01297188	-3.81326389	-3.69175196
H	5.00354719	-3.50911999	-3.31004596
H	3.71929312	-3.03557992	-4.41864491
C	4.12367821	-5.15777588	-4.39357805
H	4.85065603	-5.12243080	-5.21915483
H	4.44919205	-5.94715023	-3.69689107
H	3.15488291	-5.46978092	-4.81601810
C	0.26688999	-4.69844198	-0.52947402
H	1.07562995	-5.18101215	-1.10127997
H	0.46992001	-4.91217422	0.53394198
C	-1.06443095	-5.33758783	-0.91324103
H	-1.26366496	-5.14169884	-1.98161197
H	-1.87251794	-4.83260679	-0.35714799
C	-1.10958695	-6.83412504	-0.64766699
H	-2.07846904	-7.26634216	-0.94096398
H	-0.32444000	-7.36558104	-1.20964205
H	-0.95685703	-7.05520678	0.42124701
C	3.42401910	-4.17926502	0.97949201
H	2.82498598	-5.01078701	0.57520801
H	4.25386477	-4.02074814	0.26960301
C	4.00178003	-4.59057617	2.33057809
H	4.59693480	-3.75423908	2.73785996
H	3.17375112	-4.74600077	3.04440594

C	4.85915804	-5.84440804	2.25801802
H	5.71533918	-5.70530605	1.57826102
H	5.25998497	-6.11553383	3.24653792
H	4.27933216	-6.70473194	1.88653004
C	1.76768100	4.15971518	2.48204803
H	0.68050802	4.29256201	2.61355710
H	2.10300207	4.98251009	1.83053696
C	2.44938707	4.29021597	3.84093499
H	2.11948299	3.46104789	4.49131298
H	3.53798699	4.15678692	3.71241593
C	2.17183995	5.62067890	4.52311182
H	2.52294111	6.46598387	3.90942907
H	1.09308505	5.76439619	4.69625521
H	2.67687511	5.68572903	5.49880886
C	-0.54609501	4.62354422	-0.14915600
H	0.00411600	5.22004223	0.59600103
H	-0.11862900	4.88702488	-1.13161397
C	-2.01687908	5.03103113	-0.12312400
H	-2.58425212	4.42301416	-0.84901702
H	-2.43951797	4.78392076	0.86686802
C	-2.23105502	6.50779676	-0.41665700
H	-3.29933596	6.77107000	-0.38865599
H	-1.71127200	7.14382792	0.31820101
H	-1.84877503	6.77670717	-1.41473401
C	2.92363310	4.64895821	-0.83956897
H	3.58081794	4.59943199	0.04545600
H	2.12765598	5.37016487	-0.59418899
C	3.72917008	5.17730808	-2.02295709
H	4.52500296	4.45225811	-2.26925206
H	3.07659793	5.22165394	-2.91263509
C	4.34144402	6.54546499	-1.76654506
H	3.56512189	7.29738092	-1.55140305
H	5.02644587	6.52223682	-0.90358901
H	4.91480923	6.89919090	-2.63686204
C	-0.50395298	-0.08463400	-0.11569500
C	-1.74744201	-0.19441800	-0.02948700
C	-3.14693093	-0.13141100	-0.02190900
C	-4.01205921	-1.15184104	0.54955798
C	-3.73813391	0.99527103	-0.62064701
C	-5.41390800	-0.99825001	0.45884001
C	-3.46655607	-2.31698298	1.24596500
C	-5.11489820	1.14986205	-0.71495402
H	-3.10042810	1.77057803	-1.04622495
C	-6.29336309	-1.97166097	0.99636298
C	-5.97886515	0.15240100	-0.18360400
O	-2.26735711	-2.52236104	1.39873099
N	-4.37661505	-3.24118209	1.76139295
C	-5.67190123	2.33483505	-1.36233497
C	-5.76450205	-3.15540910	1.67502904
C	-7.68656111	-1.81349897	0.88888198
C	-7.37051916	0.28992000	-0.27477801

C	-3.80213189	-4.38354492	2.45000005
O	-4.98862791	3.23601198	-1.83452797
N	-7.06300783	2.41332006	-1.42965806
O	-6.48345709	-4.02421188	2.15029407
C	-8.21960258	-0.70307302	0.26355600
H	-8.32638931	-2.58840609	1.31371701
C	-7.95820522	1.46445298	-0.92688900
H	-3.24590397	-5.01972723	1.74615896
H	-3.10468793	-4.04225922	3.22605109
H	-4.62344408	-4.95385313	2.89335704
C	-7.60313797	3.59396791	-2.07929301
H	-9.29834843	-0.56453598	0.17343201
O	-9.16673756	1.62244499	-1.03275502
H	-7.23352194	3.66125298	-3.11193609
H	-8.69392014	3.51314902	-2.07128191
H	-7.28565407	4.50100613	-1.54587305
H	9.93801975	0.85262001	0.41330099

Frequencies

Mode	IR frequency	IR intensity
1	7.60550000	0.12080000
2	8.98670000	0.12340000
3	10.52830000	0.14710000
4	11.18430000	0.18940000
5	16.25360000	0.10320000
6	16.95550000	0.22610000
7	19.84530000	0.37840000
8	22.17810000	0.54860000
9	25.78040000	0.02640000
10	34.66140000	0.63360000
11	39.56560000	0.03700000
12	40.09370000	0.02380000
13	40.67390000	0.09200000
14	41.51810000	0.00950000
15	45.08260000	0.76800000
16	51.20040000	0.02600000
17	53.93880000	0.06050000
18	56.68250000	0.32380000
19	61.59510000	0.64740000
20	63.55060000	0.21680000
21	66.79470000	0.58350000
22	68.12010000	0.76560000
23	70.03160000	0.21650000
24	74.21080000	0.23020000
25	77.98830000	0.18070000
26	80.55330000	0.35370000
27	81.90890000	0.36330000
28	87.58870000	2.64840000
29	90.84080000	1.58880000
30	96.03220000	0.65530000
31	100.12580000	0.38010000

32	105.11580000	5.24690000
33	106.92510000	0.94470000
34	113.04410000	6.04450000
35	114.80190000	0.39010000
36	116.76510000	0.02400000
37	122.04040000	2.03300000
38	123.33720000	6.47540000
39	135.02890000	14.33730000
40	139.04760000	9.89700000
41	139.47430000	15.95430000
42	143.64140000	1.62490000
43	149.17020000	3.21200000
44	150.93920000	3.93420000
45	161.03400000	1.35040000
46	164.00120000	0.25880000
47	164.97830000	10.36390000
48	167.04980000	14.25600000
49	168.39580000	35.44780000
50	173.18800000	1.90920000
51	174.54800000	2.49850000
52	177.03600000	2.86520000
53	198.50060000	7.23350000
54	202.72150000	62.24990000
55	214.80770000	267.07700000
56	218.50800000	2.95980000
57	219.67790000	32.61860000
58	223.01440000	27.15090000
59	224.02200000	28.88560000
60	225.93640000	36.92890000
61	233.64270000	0.84200000
62	234.65940000	0.34440000
63	257.13980000	0.13720000
64	257.86250000	0.15900000
65	258.26590000	0.04840000
66	258.41260000	0.24610000
67	259.02320000	0.07740000
68	259.84920000	0.25730000
69	269.18110000	2.88170000
70	282.60750000	0.51310000
71	294.05310000	58.06920000
72	300.92720000	1.39890000
73	311.86430000	4.33900000
74	328.83770000	120.31280000
75	337.14240000	4.85590000
76	338.74190000	46.44730000
77	341.73800000	7.02120000
78	343.36930000	11.85700000
79	348.80470000	11.18240000
80	371.07600000	97.74930000
81	374.71850000	56.69680000
82	377.93200000	15.47580000

83	383.42720000	72.26530000
84	389.19010000	151.55350000
85	404.82670000	1.51510000
86	410.43190000	9.46460000
87	411.03400000	18.92510000
88	412.34340000	82.53590000
89	413.69500000	11.69290000
90	414.05250000	2.16830000
91	420.66470000	21.63010000
92	421.56050000	94.19550000
93	437.65110000	37.16070000
94	442.44120000	24.13300000
95	462.00620000	88.79700000
96	484.42190000	10.83030000
97	502.27280000	55.23430000
98	507.99130000	83.05690000
99	520.92060000	364.17320000
100	523.98120000	53.99590000
101	546.24160000	9.16030000
102	549.36870000	4.20440000
103	565.04270000	9.19320000
104	572.56810000	0.52900000
105	577.43470000	14.87540000
106	587.27310000	127.69890000
107	592.74260000	27.50320000
108	607.72340000	251.24080000
109	630.30090000	48.27690000
110	630.83730000	217.35850000
111	661.42390000	10.56610000
112	691.82500000	30.67470000
113	692.94410000	27.40130000
114	702.87350000	8.14980000
115	706.22900000	21.80640000
116	711.99170000	51.35690000
117	722.54200000	23.36070000
118	722.91540000	22.92460000
119	723.52180000	4.43810000
120	728.33080000	14.97400000
121	741.21320000	1.47590000
122	743.65590000	2.15100000
123	756.27190000	1.50410000
124	756.62590000	3.10940000
125	757.48820000	22.39360000
126	762.59750000	1.56730000
127	763.04470000	5.64850000
128	763.56340000	6.24180000
129	765.45670000	16.88730000
130	765.65810000	42.26320000
131	779.41540000	4.57740000
132	788.17360000	77.24060000
133	789.72000000	3.99330000

134	792.30000000	15.40590000
135	792.48010000	4.46570000
136	794.01740000	8.73600000
137	794.65210000	65.83810000
138	797.17000000	26.71350000
139	818.96030000	1178.00690000
140	851.52040000	2.89050000
141	863.05870000	502.61490000
142	864.24350000	0.09500000
143	872.29490000	6.79570000
144	885.25250000	5.70120000
145	885.86210000	5.89120000
146	904.85350000	0.24550000
147	905.01920000	3.29470000
148	905.47820000	21.42630000
149	906.50280000	12.14840000
150	915.30930000	4.17240000
151	915.66790000	15.31270000
152	915.71970000	7.75040000
153	915.93100000	2.78690000
154	916.62630000	45.43570000
155	917.72100000	20.19670000
156	920.75960000	829.27310000
157	947.27170000	13.27190000
158	955.20750000	5.32250000
159	969.67320000	189.46950000
160	1010.63520000	202.06980000
161	1011.54900000	1.59660000
162	1014.64290000	0.08660000
163	1028.52010000	53.61420000
164	1034.08160000	0.06410000
165	1036.96800000	10.91600000
166	1038.84720000	28.20870000
167	1042.13430000	616.83970000
168	1051.66520000	2.19240000
169	1052.24600000	7.51180000
170	1052.68180000	4.74040000
171	1053.05980000	0.49700000
172	1053.31920000	4.57530000
173	1054.20690000	5.56840000
174	1055.04580000	29.41400000
175	1060.82670000	162.64780000
176	1075.21270000	2.84600000
177	1076.13130000	4.31930000
178	1076.82470000	25.41870000
179	1079.15960000	6.57480000
180	1087.36580000	102.66850000
181	1088.96090000	2.09410000
182	1089.89730000	0.30170000
183	1090.05910000	2.90290000
184	1090.27040000	4.43460000

185	1092.36370000	7.13980000
186	1092.76080000	1.75270000
187	1101.28720000	12.18730000
188	1116.28820000	5.91790000
189	1116.53230000	1.53120000
190	1117.22700000	40.31690000
191	1117.30970000	20.50090000
192	1120.69040000	121.14740000
193	1123.23360000	230.12680000
194	1140.37440000	1.55870000
195	1141.61900000	0.21760000
196	1156.54130000	0.40660000
197	1165.31880000	0.24510000
198	1177.01680000	2148.85290000
199	1181.34660000	2.88860000
200	1212.84620000	174.48930000
201	1213.92780000	0.97730000
202	1215.81720000	4.04200000
203	1216.82290000	2.29030000
204	1217.44930000	7.90820000
205	1217.63350000	6.07910000
206	1218.42320000	21.74230000
207	1227.74610000	121.68840000
208	1229.57830000	1.75940000
209	1230.16040000	1.91240000
210	1230.53540000	1.82150000
211	1234.23920000	5.55160000
212	1248.81210000	233.10700000
213	1250.71640000	221.24320000
214	1256.00450000	226.36840000
215	1261.80890000	19.92310000
216	1267.12150000	5197.03530000
217	1291.30920000	0.76670000
218	1292.88850000	1.60490000
219	1294.88950000	3.30970000
220	1296.90250000	12.01490000
221	1297.30260000	29.56370000
222	1299.66340000	4.90900000
223	1313.63940000	1274.02550000
224	1317.05850000	0.12990000
225	1319.15170000	2.59640000
226	1319.35350000	1.69980000
227	1319.60520000	15.88910000
228	1319.95610000	0.87120000
229	1319.97990000	5.27020000
230	1321.10280000	3.21370000
231	1321.56830000	252.10560000
232	1322.13300000	620.47790000
233	1322.30690000	23.20860000
234	1323.28640000	363.27190000
235	1324.14610000	418.39280000

236	1326.98700000	15.46060000
237	1330.09680000	19.16700000
238	1368.67570000	686.04150000
239	1372.65830000	4665.34050000
240	1381.44410000	0.38920000
241	1381.73190000	0.10380000
242	1381.93980000	0.20600000
243	1382.29740000	0.57140000
244	1384.27240000	0.04290000
245	1384.86070000	1.32240000
246	1385.05820000	1.92570000
247	1398.84630000	9.49670000
248	1398.90020000	77.85790000
249	1399.00550000	11.66920000
250	1399.39460000	1.44450000
251	1400.25850000	1279.96560000
252	1401.62800000	20.56920000
253	1401.74800000	108.32350000
254	1402.19450000	14.96740000
255	1416.40740000	27.54850000
256	1420.02360000	93.79980000
257	1420.72450000	86.12550000
258	1422.60730000	63.53670000
259	1422.69890000	1588.40580000
260	1424.97640000	14.23280000
261	1430.69200000	15.28090000
262	1447.46980000	6.15670000
263	1447.70650000	5.06660000
264	1449.36720000	1.47710000
265	1449.41820000	2.50110000
266	1450.02670000	28.10330000
267	1451.48050000	6.07720000
268	1452.44950000	120.38440000
269	1452.97060000	296.37130000
270	1456.30690000	25.91820000
271	1458.44010000	8.93710000
272	1458.74050000	8.57480000
273	1458.78680000	12.19510000
274	1458.82100000	8.11860000
275	1458.82560000	9.60290000
276	1459.09650000	6.78520000
277	1460.16770000	11.52360000
278	1460.41150000	3.07370000
279	1461.73260000	120.40900000
280	1465.37010000	0.10060000
281	1465.70090000	0.34460000
282	1465.73480000	0.29540000
283	1465.88100000	0.01170000
284	1467.04100000	49.35250000
285	1473.34500000	3.92320000
286	1473.84680000	8.50150000

287	1476.70200000	14.65740000
288	1477.42040000	15.53900000
289	1477.53520000	21.74620000
290	1478.38150000	14.54540000
291	1478.91110000	10.32570000
292	1481.72490000	8.61420000
293	1492.33750000	195.20730000
294	1514.63140000	1592.20400000
295	1523.98360000	1.67480000
296	1544.28530000	489.23830000
297	1560.70720000	9353.16790000
298	1601.15940000	2653.56690000
299	1638.70720000	1.58740000
300	1659.11750000	1164.09890000
301	1671.60200000	364.38990000
302	1712.21570000	796.47890000
303	1731.79730000	2066.39180000
304	1756.56000000	1590.32360000
305	1774.14520000	2739.63730000
306	2036.28790000	22708.39400000
307	2163.60860000	3422.82410000
308	3044.51140000	33.61460000
309	3045.01470000	30.76510000
310	3045.11050000	37.10360000
311	3045.45360000	33.39990000
312	3046.59680000	38.22870000
313	3046.86520000	37.76720000
314	3048.05520000	10.34220000
315	3048.65280000	29.33070000
316	3048.72280000	10.78920000
317	3048.79560000	54.76890000
318	3048.95330000	33.40720000
319	3049.33590000	10.79070000
320	3055.89210000	35.26330000
321	3058.94470000	28.41910000
322	3059.23710000	22.85890000
323	3060.78880000	15.65380000
324	3061.12860000	23.19020000
325	3061.97330000	44.72260000
326	3064.80600000	47.47860000
327	3067.06980000	46.77750000
328	3067.11540000	8.54190000
329	3067.63360000	29.44880000
330	3069.15380000	21.25000000
331	3071.48790000	14.91870000
332	3080.57690000	63.61940000
333	3081.48790000	31.87570000
334	3090.01530000	11.31070000
335	3090.05720000	11.27620000
336	3090.37130000	10.12040000
337	3090.60300000	8.89380000

338	3094.23970000	4.31210000
339	3096.40730000	2.67050000
340	3115.71150000	6.88580000
341	3116.24300000	30.26690000
342	3116.57480000	21.06350000
343	3118.61930000	13.41040000
344	3119.14740000	12.40430000
345	3120.10090000	13.24990000
346	3131.26970000	4.06820000
347	3131.42450000	13.09330000
348	3132.41620000	11.40830000
349	3135.13990000	2.76690000
350	3136.57140000	10.17650000
351	3136.89070000	2.10060000
352	3137.67030000	57.92850000
353	3138.79820000	53.92270000
354	3139.03150000	52.17480000
355	3139.14180000	53.53300000
356	3139.23630000	54.38810000
357	3139.26470000	64.35200000
358	3146.31650000	28.86150000
359	3147.65000000	27.79900000
360	3147.97900000	37.38590000
361	3148.03490000	24.38170000
362	3148.07140000	36.56440000
363	3148.30360000	30.82260000
364	3168.31500000	11.97030000
365	3172.22450000	11.30970000
366	3207.33550000	10.89740000
367	3213.27030000	0.19050000
368	3220.79820000	8.32970000
369	3226.36000000	16.07230000
370	3229.76500000	3.39930000
371	3230.02830000	1.35970000
372	3232.64810000	179.52190000
373	3233.77450000	5.25100000
374	3243.98030000	5.15800000
375	3246.54060000	22.45400000

5: PTZ-CH_2-Ph-CC-Pt-CC-NDI in CH_2Cl_2, SINGLET

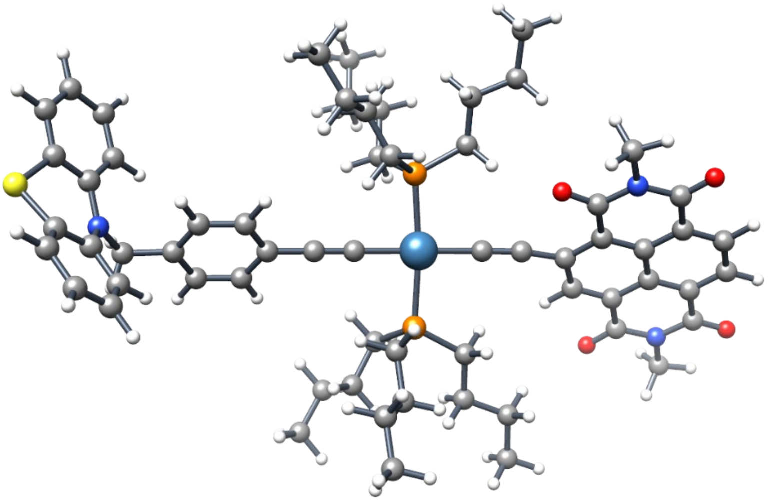

Optimised singlet ground state geometry of PTZ-CH_2-Ph-CC-Pt-CC-NDI.

Route	:	#p opt freq PBE1PBE/genecp
	:	scrf=(cpcm,solvent=dichloromethane) geom=connectivity
	:	int=acc2e=13
SMILES	:	CCCC[P](CCCC)(CCCC)[Pt]
	:	(C#Cc1ccc(cc1)CN2c3ccccc3Sc4c2cccc4)
	:	(C#Cc5cc6c7c(ccc8c7c5C(=O)N(C8=O)C)
	:	C(=O)N(C6=O)C)[P](CCCC)(CCCC)CCCC
Formula	:	$C_{63}H_{77}N_3O_4P_2PtS$
Charge	:	0
Multiplicity	:	1
Dipole	:	9.3905 Debye
Energy	:	-4106.57159541 a.u.
Gibbs Energy	:	-4105.43083300 a.u.
Number of imaginary frequencies	:	0

Cartesian Co-ordinates (XYZ format)

151

Pt	-0.67170203	0.09829000	-0.34161201
C	1.30708897	0.40870401	-0.47785801
P	-0.89738500	2.42610598	-0.10244000
P	-0.19926099	-2.18504190	-0.59729600
C	2.52306104	0.59723002	-0.55508399

C	3.93193412	0.81645697	-0.65006602
C	4.84685802	0.00463600	0.04656300
C	4.45505810	1.85690606	-1.44591796
C	6.21850491	0.22479101	-0.04627900
H	4.46972179	-0.80804300	0.67157900
C	5.82482195	2.06806111	-1.53534997
H	3.76765895	2.50255704	-1.99731505
C	6.73002911	1.25705898	-0.83768499
H	6.90265894	-0.41891900	0.51224101
H	6.20079184	2.88446593	-2.15947294
C	-0.08201200	3.28625488	-1.51364195
H	0.75430202	2.62433600	-1.78883696
H	-0.80576098	3.22989106	-2.34592891
C	-2.63480711	3.02665591	-0.02850700
H	-3.00999498	2.73025489	0.96646702
H	-3.17520595	2.38341308	-0.74067497
C	-0.05293200	2.92037702	1.45939004
H	-0.29134399	2.11179495	2.17061090
H	1.02423000	2.81282902	1.24495006
C	-1.65568697	-3.30384302	-0.54600197
H	-2.15949893	-3.17714691	-1.52051795
H	-2.33970594	-2.85921693	0.19497900
C	0.62866902	-2.40524602	-2.22922611
H	0.07973000	-1.73198199	-2.90928411
H	1.62470901	-1.94677103	-2.10649109
C	0.96107101	-2.72107196	0.73215002
H	1.55294096	-1.81970799	0.95608097
H	0.32749999	-2.90171409	1.61829305
C	0.72388101	-3.79379201	-2.85299802
H	-0.28872600	-4.20670700	-2.99960303
H	1.24617004	-4.49012709	-2.17723298
C	1.44505501	-3.76803589	-4.19738817
H	2.45931602	-3.35523200	-4.05468416
H	0.92653698	-3.06347203	-4.87143612
C	1.53517795	-5.13622093	-4.85524797
H	2.06264901	-5.08595181	-5.82000589
H	2.07712793	-5.85235310	-4.21653509
H	0.53398502	-5.55587482	-5.04492903
C	-1.43762696	-4.78069019	-0.23091900
H	-0.74865299	-5.24410200	-0.95574301
H	-0.95622700	-4.87988377	0.75721699
C	-2.74860001	-5.56175804	-0.21827000
H	-3.23516297	-5.46542883	-1.20504797
H	-3.43637490	-5.08963823	0.50433999
C	-2.56647205	-7.03257895	0.12236200
H	-3.52784896	-7.56875181	0.12282800
H	-1.90654099	-7.53372097	-0.60437602
H	-2.11439204	-7.15954590	1.11944795
C	1.90155303	-3.89616895	0.48288199
H	1.33756995	-4.80590916	0.22165599
H	2.54706407	-3.67269993	-0.38375601

C	2.78462791	-4.18824720	1.69248796
H	3.34156609	-3.27320194	1.96155000
H	2.14274192	-4.41537380	2.56191301
C	3.75895691	-5.33256578	1.45993400
H	4.43589687	-5.11581993	0.61780798
H	4.38182688	-5.51868916	2.34810400
H	3.22791505	-6.26888514	1.22366095
C	-0.36418900	4.27090597	2.09451890
H	-1.43991196	4.33071280	2.33235097
H	-0.15818600	5.09052992	1.38761103
C	0.43718800	4.50287724	3.37222099
H	0.23807500	3.67753100	4.07836580
H	1.51466095	4.44426203	3.13770604
C	0.12810799	5.83465576	4.03814411
H	0.35026500	6.67929697	3.36604905
H	-0.93595397	5.90554285	4.31596899
H	0.72124499	5.97421408	4.95471191
C	-2.94180393	4.49202585	-0.31968600
H	-2.39079595	5.15387678	0.36769399
H	-2.59742999	4.74809408	-1.33610797
C	-4.43365192	4.79674482	-0.21294101
H	-4.99302912	4.13551092	-0.89800900
H	-4.78129196	4.53887606	0.80313802
C	-4.76993608	6.24867678	-0.51599699
H	-5.85050583	6.43837786	-0.42726400
H	-4.25356913	6.93289185	0.17669600
H	-4.46717978	6.52482080	-1.53903496
C	0.43301901	4.71086407	-1.33167899
H	1.18956804	4.72824192	-0.52857101
H	-0.37573099	5.38550806	-1.00827301
C	1.05809999	5.26151896	-2.61006093
H	1.86403704	4.58216381	-2.94000006
H	0.30368000	5.24562883	-3.41635609
C	1.60790503	6.67024422	-2.44913101
H	0.81607300	7.37770319	-2.15420794
H	2.38964009	6.70837116	-1.67329001
H	2.05124497	7.03948784	-3.38645601
C	-2.62609410	-0.19777900	-0.17229600
C	-3.83880806	-0.37827399	-0.00998600
C	-5.24877787	-0.38290501	0.09035600
C	-6.02207422	-1.25317705	0.88652402
C	-5.93658686	0.61157203	-0.67545700
C	-7.43511009	-1.11496103	0.92600298
C	-5.39231777	-2.31830597	1.69321597
C	-7.30048990	0.75185603	-0.64104700
H	-5.35515499	1.28682005	-1.30435705
C	-8.23536968	-1.96398699	1.72664499
C	-8.08397293	-0.11134600	0.16211200
O	-4.19531679	-2.53424597	1.70923400
N	-6.22992516	-3.12105608	2.47594309
C	-7.95087624	1.81745803	-1.44192195

C	-7.61338806	-3.02428102	2.54946589
C	-9.61487103	-1.81497598	1.75712395
C	-9.48499775	0.02453500	0.20386900
C	-5.56975508	-4.15168905	3.26034498
O	-7.32376909	2.59680891	-2.13207197
N	-9.33726692	1.90028596	-1.37229800
O	-8.26985836	-3.77090597	3.25098705
C	-10.24542427	-0.82277399	0.99644601
H	-10.19440079	-2.49004197	2.38929796
C	-10.15807438	1.07537699	-0.60038501
H	-5.11360884	-4.90290213	2.59963989
H	-4.77619219	-3.70487905	3.87274599
H	-6.32353306	-4.62560177	3.89531708
C	-9.95708466	2.94719601	-2.16911507
H	-11.32955170	-0.70083702	1.01495099
O	-11.36376667	1.21793795	-0.59119803
H	-9.69980335	2.81531692	-3.22867393
H	-11.03987694	2.88021302	-2.03189492
H	-9.59505177	3.93286896	-1.84589100
C	8.21523476	1.53390396	-0.96979201
H	8.40491962	2.59406590	-0.73576999
H	8.51446247	1.41042304	-2.02337193
N	9.11505127	0.73758698	-0.16220200
C	9.54722309	-0.50446701	-0.65369397
C	9.34715557	1.12622499	1.16696703
C	10.69285965	-1.13446999	-0.12727500
C	8.86226940	-1.16055703	-1.68937194
C	10.47261429	0.65877801	1.87518597
C	8.47498894	2.00070596	1.83539903
C	11.10865211	-2.37840390	-0.60101199
S	11.70860767	-0.29369599	1.04760396
C	9.30634499	-2.38588595	-2.18623400
H	7.96060514	-0.71690702	-2.11223197
C	10.68902302	1.02840602	3.20226407
C	8.71822929	2.39793205	3.15007210
H	7.58397818	2.37202191	1.32912195
C	10.42598248	-3.00729489	-1.64145696
H	11.99429798	-2.84252596	-0.15952800
H	8.75304317	-2.85975790	-3.00038791
H	11.56552982	0.63540602	3.72376108
C	9.82005215	1.90927196	3.84536600
H	8.02229118	3.08795500	3.63284397
H	10.77033138	-3.97241592	-2.01871896
H	10.00801563	2.20783710	4.87874794

Frequencies

Mode	IR frequency	IR intensity
1	3.81110000	0.08340000
2	5.90140000	0.07250000
3	8.41870000	0.06200000
4	9.27230000	0.02980000
5	11.98640000	0.14650000
6	13.52570000	0.05880000
7	15.26280000	0.20550000
8	18.06000000	0.00910000
9	21.71330000	0.26080000
10	25.39590000	0.47050000
11	27.54480000	0.31960000
12	36.11930000	0.05470000
13	40.30210000	0.02550000
14	40.81730000	0.01170000
15	41.30720000	0.08130000
16	42.48310000	0.11470000
17	44.38030000	0.13840000
18	47.15690000	0.10230000
19	51.24540000	0.21780000
20	53.29120000	0.81690000
21	54.85700000	0.13560000
22	55.81960000	0.39720000
23	61.86430000	0.04590000
24	62.54040000	0.10040000
25	64.89720000	0.19120000
26	66.71730000	0.22040000
27	69.20530000	0.31020000
28	73.82620000	0.33490000
29	76.41120000	1.66990000
30	80.68460000	1.44760000
31	83.71260000	1.30360000
32	87.21580000	1.28070000
33	88.98050000	2.01260000
34	89.68900000	1.03770000
35	94.76530000	2.19870000
36	94.85250000	4.14440000
37	100.96990000	0.77110000
38	103.64340000	5.70560000
39	106.43050000	6.16660000
40	114.69040000	0.03750000
41	117.85060000	0.15690000
42	121.38960000	2.20550000
43	122.42700000	4.16260000
44	131.03490000	0.18840000
45	138.00770000	0.05760000
46	140.19680000	7.90660000
47	141.33570000	1.85570000
48	142.55570000	2.09460000

49	151.60010000	1.42090000
50	153.06380000	1.62790000
51	161.58510000	0.94850000
52	164.18850000	0.05190000
53	165.32230000	1.25830000
54	168.91300000	0.05180000
55	170.41070000	0.14850000
56	171.63400000	0.55050000
57	172.70660000	1.11970000
58	177.37900000	0.82460000
59	183.67080000	0.57770000
60	194.89400000	0.10590000
61	198.32490000	0.28180000
62	204.95690000	0.39740000
63	213.22940000	13.21080000
64	218.57420000	1.19260000
65	218.79760000	0.44230000
66	222.79840000	0.87160000
67	224.49690000	0.41170000
68	232.22990000	2.84980000
69	234.12220000	0.29430000
70	235.30820000	0.08790000
71	244.04060000	0.67860000
72	247.61580000	2.98710000
73	254.22750000	0.07160000
74	257.71700000	0.24130000
75	258.77730000	0.06800000
76	260.03890000	0.09820000
77	260.24270000	0.08610000
78	262.05670000	0.09910000
79	274.29650000	0.12980000
80	284.49110000	3.23860000
81	299.17290000	2.30050000
82	303.09620000	2.52270000
83	311.88860000	2.48350000
84	324.33480000	0.54040000
85	329.20960000	2.99220000
86	335.25620000	0.77640000
87	337.26220000	0.54120000
88	337.99730000	3.16530000
89	340.36020000	9.31140000
90	341.77840000	23.92900000
91	358.00940000	6.27480000
92	368.13610000	6.85750000
93	373.80060000	33.13790000
94	376.45810000	13.43540000
95	378.97810000	17.20530000
96	384.11840000	0.91310000
97	386.65040000	5.52170000
98	391.54900000	21.06670000
99	405.05990000	0.07400000

100	409.76090000	6.00260000
101	411.42620000	13.56700000
102	412.21040000	98.04390000
103	414.62920000	16.61160000
104	418.65270000	0.59370000
105	421.55430000	2.61060000
106	423.51110000	0.62460000
107	433.57320000	6.20550000
108	447.13370000	2.27790000
109	452.15120000	5.95490000
110	454.00090000	12.52160000
111	455.65910000	0.68790000
112	462.82270000	0.45650000
113	464.78830000	0.61700000
114	485.33470000	7.91510000
115	498.13550000	78.05510000
116	505.13050000	46.40220000
117	516.02360000	17.24820000
118	521.15020000	20.87260000
119	524.04920000	13.86790000
120	532.88880000	1.01460000
121	549.47290000	7.20050000
122	551.90670000	0.42490000
123	552.37740000	14.85990000
124	569.78590000	9.90390000
125	578.89170000	1.79160000
126	579.46580000	0.38540000
127	587.78690000	6.02530000
128	596.90960000	16.36330000
129	612.73430000	1.26050000
130	615.41550000	4.50830000
131	635.24380000	1.30030000
132	642.82950000	5.50290000
133	665.30610000	12.28200000
134	686.93410000	1.46480000
135	691.51220000	16.72580000
136	693.42670000	9.89650000
137	694.18700000	12.09790000
138	702.21610000	3.26190000
139	704.29760000	2.84020000
140	715.83330000	2.49400000
141	720.27000000	28.01800000
142	721.61110000	16.11800000
143	724.39140000	22.39870000
144	726.85560000	15.92560000
145	739.42180000	0.74700000
146	742.05190000	7.56570000
147	749.68350000	0.00900000
148	754.78620000	25.12390000
149	755.77430000	1.04710000
150	756.17360000	1.18380000

151	757.10510000	0.06840000
152	760.57860000	54.58880000
153	760.93230000	2.24090000
154	763.24810000	6.70920000
155	763.93250000	5.69750000
156	765.02220000	8.11030000
157	767.69710000	0.33710000
158	769.88980000	21.28220000
159	770.40880000	23.11490000
160	774.98580000	102.40280000
161	786.60700000	4.99140000
162	787.57440000	9.21870000
163	788.75350000	11.00220000
164	791.55300000	45.53500000
165	793.24270000	91.28220000
166	794.29230000	21.03590000
167	800.03430000	0.25090000
168	813.71720000	42.90520000
169	841.44210000	14.75560000
170	855.02190000	3.72450000
171	858.44470000	0.02690000
172	861.47110000	0.08770000
173	869.33760000	4.40810000
174	881.05620000	3.89630000
175	881.66940000	2.71860000
176	884.14100000	2.29760000
177	884.88830000	2.92010000
178	888.91230000	76.32610000
179	903.34790000	22.13160000
180	904.61850000	2.30360000
181	905.22450000	29.27060000
182	906.34160000	10.30400000
183	906.50380000	23.33070000
184	913.46110000	10.20780000
185	914.84550000	13.47310000
186	915.01310000	1.27150000
187	915.30450000	11.29160000
188	915.37590000	9.84170000
189	916.34560000	4.51660000
190	929.32890000	5.75630000
191	965.66670000	3.73410000
192	966.13830000	6.21310000
193	973.49630000	15.01340000
194	976.33340000	0.28280000
195	986.63220000	1.82760000
196	1007.15050000	0.14430000
197	1010.47800000	0.34150000
198	1010.65380000	0.07500000
199	1027.09740000	0.07110000
200	1028.58730000	6.19980000
201	1029.80730000	23.79720000

202	1030.61220000	83.54470000
203	1036.45280000	2.46410000
204	1036.73950000	1.49760000
205	1047.86870000	28.59790000
206	1051.26160000	3.61800000
207	1051.56770000	3.65560000
208	1051.93340000	5.62490000
209	1053.51780000	5.96280000
210	1053.69920000	7.04910000
211	1054.59300000	0.87560000
212	1067.94630000	104.44440000
213	1073.05240000	8.03430000
214	1074.68890000	1.48730000
215	1075.76230000	14.42810000
216	1075.84810000	23.40930000
217	1075.89460000	23.45020000
218	1076.42440000	16.84710000
219	1078.48730000	4.54300000
220	1089.60940000	3.60580000
221	1089.73180000	2.01500000
222	1090.64730000	2.29200000
223	1090.88320000	1.37310000
224	1093.01400000	4.02830000
225	1093.78170000	0.94820000
226	1095.11240000	25.20910000
227	1096.02080000	1.74970000
228	1116.30880000	12.44100000
229	1116.84490000	8.84820000
230	1117.25250000	37.01200000
231	1117.73240000	25.44060000
232	1120.98230000	118.93080000
233	1124.02390000	4.19420000
234	1127.16010000	14.66650000
235	1141.68820000	0.97330000
236	1142.23070000	0.23060000
237	1151.33890000	15.34350000
238	1151.86990000	22.20400000
239	1158.48390000	11.69730000
240	1162.14840000	87.49700000
241	1170.79620000	5.08900000
242	1175.58190000	3.44020000
243	1186.58180000	9.09360000
244	1189.06630000	13.15700000
245	1205.19020000	198.66300000
246	1213.41040000	3.90010000
247	1214.57440000	2.34950000
248	1215.70170000	2.33280000
249	1216.68900000	2.20580000
250	1217.89400000	4.20260000
251	1218.34210000	7.81660000
252	1226.96800000	1.03830000

253	1228.63180000	2.76650000
254	1228.77790000	42.36790000
255	1229.43160000	1.76410000
256	1231.60640000	0.47260000
257	1231.77970000	0.48280000
258	1247.83690000	31.63510000
259	1248.65280000	9.92780000
260	1251.89530000	124.75180000
261	1252.97540000	40.96300000
262	1261.18240000	42.52050000
263	1275.12640000	4.51180000
264	1289.45710000	37.14850000
265	1291.04820000	3.28990000
266	1292.09710000	2.62740000
267	1293.90100000	2.57450000
268	1295.71560000	1.68360000
269	1296.10980000	7.56380000
270	1298.44880000	9.42370000
271	1306.08490000	300.66840000
272	1307.31120000	1.71410000
273	1316.54780000	0.74290000
274	1317.10330000	2.48700000
275	1317.59570000	2.76340000
276	1320.02230000	1.30020000
277	1320.57860000	4.64350000
278	1321.15920000	1.03710000
279	1322.13390000	1.32600000
280	1324.41660000	0.70520000
281	1324.76970000	4.60490000
282	1325.80090000	2.74220000
283	1326.36080000	16.62790000
284	1328.77290000	14.14260000
285	1331.96540000	38.25300000
286	1334.71960000	332.44690000
287	1344.72770000	125.97920000
288	1347.04230000	673.45350000
289	1370.43400000	124.34670000
290	1375.32350000	99.71570000
291	1380.65430000	0.21650000
292	1381.39740000	0.42140000
293	1381.50070000	0.24680000
294	1381.94620000	0.18620000
295	1383.78190000	0.25120000
296	1384.04240000	0.45200000
297	1388.63640000	383.71640000
298	1392.07600000	108.31740000
299	1396.42930000	9.92190000
300	1398.20950000	1.06230000
301	1398.92660000	3.03960000
302	1398.98070000	1.08190000
303	1399.55020000	1.12350000

304	1401.66060000	6.08850000
305	1402.02370000	12.04370000
306	1404.65950000	24.10860000
307	1409.17140000	99.29020000
308	1415.98450000	140.67850000
309	1420.03820000	31.51260000
310	1421.27900000	8.27430000
311	1421.38460000	24.51400000
312	1423.04980000	14.30600000
313	1426.16520000	24.09680000
314	1428.95440000	31.83370000
315	1443.06000000	158.53820000
316	1444.03460000	40.47610000
317	1447.13760000	1.90000000
318	1447.68600000	2.42690000
319	1449.28870000	1.79040000
320	1449.89980000	1.32030000
321	1450.88040000	2.22250000
322	1451.78320000	2.52630000
323	1454.61570000	32.79680000
324	1455.36910000	17.33170000
325	1456.78880000	8.83010000
326	1457.32340000	17.03330000
327	1458.07290000	10.72860000
328	1458.72010000	9.24200000
329	1458.94450000	9.43130000
330	1459.11340000	9.97320000
331	1459.31760000	7.78030000
332	1459.88900000	1.03670000
333	1460.30160000	2.37260000
334	1462.30800000	24.11610000
335	1465.31050000	0.24440000
336	1465.71790000	0.52080000
337	1466.10550000	10.57560000
338	1466.59280000	7.26020000
339	1466.66970000	60.47100000
340	1473.41560000	5.00040000
341	1473.91610000	6.23250000
342	1476.41190000	23.85070000
343	1476.76880000	182.44860000
344	1477.38910000	14.14090000
345	1478.14850000	14.36620000
346	1478.68880000	22.34750000
347	1484.36260000	24.76120000
348	1485.13500000	23.85080000
349	1489.87470000	348.65640000
350	1501.79440000	337.73530000
351	1514.32900000	701.52370000
352	1520.60620000	279.87250000
353	1543.10680000	70.29300000
354	1549.27090000	89.88660000

355	1570.17170000	31.27910000
356	1629.97560000	12.72280000
357	1638.27010000	507.80720000
358	1644.20330000	0.39220000
359	1656.51730000	66.46620000
360	1661.18660000	0.49800000
361	1676.95770000	100.21800000
362	1683.06770000	71.81200000
363	1691.95260000	37.30580000
364	1699.22710000	172.15480000
365	1768.01350000	1104.55360000
366	1779.77070000	503.38990000
367	1809.86330000	660.22000000
368	1825.41160000	389.48920000
369	2172.60980000	3048.98410000
370	2210.26780000	35.46700000
371	3043.77570000	36.65380000
372	3044.29090000	33.19680000
373	3044.84980000	33.57080000
374	3045.13690000	29.49470000
375	3045.21080000	31.16540000
376	3046.09400000	37.20160000
377	3047.98340000	26.29500000
378	3048.48400000	11.36030000
379	3048.49320000	27.97960000
380	3048.56240000	48.73530000
381	3048.73190000	37.50890000
382	3048.84830000	11.74900000
383	3057.63930000	43.06430000
384	3058.50590000	14.07300000
385	3058.56550000	23.21850000
386	3059.11380000	42.33880000
387	3059.54190000	32.07160000
388	3059.99340000	35.96020000
389	3065.14940000	28.41970000
390	3066.00190000	35.23240000
391	3066.01270000	35.91610000
392	3066.85090000	31.90300000
393	3067.04770000	33.47040000
394	3067.14500000	25.67110000
395	3071.97600000	19.34020000
396	3085.88180000	28.02390000
397	3087.44740000	28.48880000
398	3088.79690000	16.67760000
399	3089.21730000	15.03270000
400	3089.67000000	10.98950000
401	3090.14910000	11.31590000
402	3090.89060000	7.78460000
403	3094.57970000	2.94150000
404	3114.47160000	5.88530000
405	3114.72770000	7.11860000

406	3114.94030000	7.94450000
407	3115.47950000	41.74680000
408	3118.16480000	22.38030000
409	3118.83220000	16.59820000
410	3119.41360000	17.96990000
411	3130.24990000	17.34880000
412	3130.49020000	18.82050000
413	3132.91900000	15.22580000
414	3135.33610000	1.47260000
415	3136.54350000	0.59260000
416	3137.30130000	44.33410000
417	3137.34580000	21.03510000
418	3138.67170000	56.54030000
419	3138.81190000	57.92310000
420	3138.95980000	62.60470000
421	3138.98870000	52.05330000
422	3139.03140000	70.62360000
423	3145.60750000	34.64500000
424	3146.99520000	33.95890000
425	3147.30520000	35.09230000
426	3147.47180000	27.68990000
427	3147.51720000	37.53000000
428	3147.67930000	30.61250000
429	3178.43080000	8.43120000
430	3179.23630000	7.55290000
431	3194.91480000	16.14570000
432	3209.17080000	9.27600000
433	3209.71200000	0.35770000
434	3209.83090000	0.85710000
435	3217.65540000	4.80400000
436	3217.78170000	6.14890000
437	3219.74940000	14.91230000
438	3224.08710000	4.22430000
439	3232.78290000	21.32760000
440	3232.92460000	5.13870000
441	3234.50920000	2.05580000
442	3235.83120000	2.54720000
443	3238.03780000	1.38660000
444	3241.53280000	2.59760000
445	3244.19170000	1.61680000
446	3246.88920000	0.91270000
447	3248.90040000	0.79040000

6: PTZ-CH_2-Ph-CC-Pt-CC-NDI in CH_2Cl_2, TRIPLET

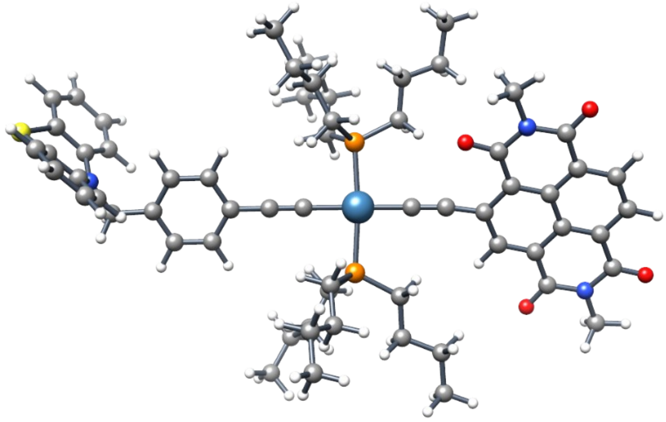

Optimised triplet ground state geometry of PTZ-CH_2-Ph-CC-Pt-CC-NDI, PTZ group puckered.

Route	:	#p opt freq PBE1PBE/genecp
	:	scrf=(cpcm,solvent=dichloromethane) geom=connectivity
	:	int=acc2e=13 scf=noincfock
SMILES	:	CCCC[P](CCCC)(CCCC)[Pt]
	:	(C#Cc1ccc(cc1)CN2c3ccccc3Sc4c2cccc4)
	:	(C#Cc5cc6c7c(ccc8c7c5C(=O)N(C8=O)C)
	:	C(=O)N(C6=O)C)[P](CCCC)(CCCC)CCCC
Formula	:	$C_{63}H_{77}N_3O_4P_2PtS^3$
Charge	:	0
Multiplicity	:	3
Dipole	:	50.5373 Debye
Energy	:	-4106.51453695 a.u.
Gibbs Energy	:	-4105.37663300 a.u.
Number of imaginary frequencies	:	0

Cartesian Co-ordinates (XYZ format)

151

Pt	0.66355300	-0.20258600	-0.33628601
C	-1.29860306	-0.52213901	-0.38247001
P	0.90930200	-2.54487610	-0.09127900
P	0.18364200	2.09471202	-0.59590203
C	-2.51704311	-0.73401803	-0.39062500
C	-3.91734695	-0.98296201	-0.40762499
C	-4.83838511	0.02075100	-0.04715700
C	-4.42453003	-2.24599504	-0.78378302

C	-6.20573616	-0.23009600	-0.05912800
H	-4.46667814	1.00408995	0.24898800
C	-5.79093218	-2.48397589	-0.79912001
H	-3.72742295	-3.03866005	-1.06439197
C	-6.70307493	-1.48194003	-0.43687901
H	-6.89968491	0.56152397	0.23277500
H	-6.16087389	-3.47025204	-1.09410596
C	0.07653000	-3.39815092	-1.49394798
H	-0.76841599	-2.74205089	-1.75582802
H	0.78736198	-3.33376288	-2.33659911
C	2.65189099	-3.12491608	-0.03955100
H	3.04054403	-2.80787110	0.94388402
H	3.17489100	-2.49394608	-0.77506799
C	0.08778500	-3.02779293	1.48430705
H	0.33820400	-2.21634698	2.18811703
H	-0.99255800	-2.92181706	1.28611195
C	1.66441298	3.15423203	-0.81848198
H	2.01388001	2.95052099	-1.84635901
H	2.43000507	2.72946501	-0.14841799
C	-0.88637400	2.29324007	-2.08208203
H	-0.46964300	1.58678901	-2.81977892
H	-1.86420000	1.87208605	-1.79385102
C	-0.73461002	2.67606306	0.88941503
H	-1.33669400	1.80776298	1.20156801
H	0.03443300	2.80951405	1.67049396
C	-1.03735399	3.66888595	-2.72208309
H	-0.04836200	4.04603577	-3.03262711
H	-1.43102801	4.39757490	-1.99529397
C	-1.95715904	3.63298512	-3.93927693
H	-2.94735694	3.25268888	-3.63229299
H	-1.56545496	2.89950204	-4.66590595
C	-2.11325598	4.98881721	-4.61003304
H	-2.78168297	4.93194389	-5.48254824
H	-2.53534293	5.73257780	-3.91497993
H	-1.14261305	5.37702990	-4.95869303
C	1.54976296	4.65498686	-0.56428099
H	0.73877198	5.10488701	-1.15895605
H	1.28972197	4.82745600	0.49424800
C	2.85597992	5.38011312	-0.87455499
H	3.10737610	5.22975779	-1.93927300
H	3.66951895	4.90401793	-0.30120799
C	2.80455208	6.86761093	-0.56299800
H	3.75790095	7.36288118	-0.80258799
H	2.01322007	7.37219095	-1.14086902
H	2.59679389	7.04487991	0.50483602
C	-1.63084197	3.90642500	0.78482401
H	-1.05714703	4.78233910	0.44239801
H	-2.41136694	3.73025298	0.02498800
C	-2.30263996	4.23759508	2.11429596
H	-2.87347388	3.35734701	2.45918107
H	-1.52437699	4.40965223	2.87854910

C	-3.22076988	5.44718122	2.03561592
H	-4.03129816	5.28837919	1.30601001
H	-3.68726110	5.66211081	3.00901389
H	-2.66895795	6.34889507	1.72458899
C	0.40824199	-4.37588978	2.12065411
H	1.48694503	-4.43479681	2.34393191
H	0.19135600	-5.19866991	1.42071795
C	-0.37577999	-4.59982300	3.41048694
H	-0.16599301	-3.77089190	4.10920095
H	-1.45633197	-4.54183722	3.19054103
C	-0.05776400	-5.92827702	4.07874012
H	-0.28919399	-6.77623987	3.41406608
H	1.00986898	-5.99783278	4.34259892
H	-0.63856000	-6.06263494	5.00387096
C	2.96133089	-4.59472799	-0.30789900
H	2.39641905	-5.24764109	0.37632999
H	2.63453293	-4.86014414	-1.32770395
C	4.45024920	-4.90052319	-0.16789401
H	5.03081083	-4.24076986	-0.83586502
H	4.77324390	-4.64547205	0.85704303
C	4.79036283	-6.35291815	-0.46402600
H	5.86830711	-6.54312086	-0.34921399
H	4.25656319	-7.03756094	0.21495400
H	4.51290321	-6.62732697	-1.49470103
C	-0.42517099	-4.82707691	-1.30744600
H	-1.16832900	-4.85227585	-0.49210599
H	0.39450601	-5.49560308	-0.99965900
C	-1.06684303	-5.37705612	-2.57780790
H	-1.88431394	-4.70330906	-2.89037895
H	-0.32621199	-5.35071707	-3.39638710
C	-1.60069501	-6.79150105	-2.41376996
H	-0.79738301	-7.49281406	-2.13594699
H	-2.36880493	-6.84060287	-1.62505996
H	-2.05635095	-7.16017580	-3.34531403
C	2.58818889	0.09544000	-0.21024400
C	3.81237793	0.28739500	-0.03711400
C	5.20807123	0.32106701	0.07717100
C	5.95223808	1.38073695	0.74213099
C	5.92239809	-0.74621600	-0.49569201
C	7.36374807	1.31935203	0.77144200
C	5.27441883	2.49281001	1.40878296
C	7.30909586	-0.81068498	-0.47038200
H	5.37652588	-1.54878795	-0.99270701
C	8.12787628	2.33202291	1.40441298
C	8.05644989	0.22392400	0.15818000
O	4.05603123	2.62052202	1.45647001
N	6.07388115	3.45805502	2.02278996
C	7.99667120	-1.94111800	-1.08902097
C	7.46636820	3.46228600	2.05759096
C	9.53241158	2.26478601	1.41839099
C	9.45647812	0.17613700	0.18952300

C	5.36809015	4.54360008	2.68059206
O	7.41738415	-2.86967611	-1.64040697
N	9.39022541	-1.93273902	-1.02907395
O	8.08361435	4.36209679	2.61149311
C	10.18902206	1.20637202	0.82126302
H	10.08054638	3.06738210	1.91413999
C	10.17425823	-0.94278902	-0.42948800
H	4.83533287	5.16182804	1.94333994
H	4.62877607	4.13862181	3.38384891
H	6.10824108	5.15247011	3.20769000
C	10.06062603	-3.06225204	-1.64729404
H	11.27818298	1.13789201	0.82495701
O	11.39506626	-1.02244794	-0.42846400
H	9.80641174	-3.11751390	-2.71504307
H	11.13823509	-2.92364693	-1.52140403
H	9.73944378	-4.00023317	-1.17323196
C	-8.18557453	-1.79583395	-0.46911401
H	-8.36692524	-2.71943903	0.10419900
H	-8.47998714	-2.03447890	-1.50414097
N	-9.08964920	-0.78104901	0.02535200
C	-9.53220367	0.21987200	-0.85506803
C	-9.31595993	-0.70084602	1.40948200
C	-10.68016148	0.98456699	-0.56576002
C	-8.85495186	0.49195901	-2.05477905
C	-10.44141102	-0.02581700	1.92331302
C	-8.43860054	-1.29702795	2.32963991
C	-11.10548210	1.99374199	-1.42898905
S	-11.68607235	0.58586502	0.82993299
C	-9.30811405	1.47616994	-2.93314195
H	-7.95272303	-0.06379000	-2.31130409
C	-10.65188408	0.07274300	3.29828000
C	-8.67636490	-1.22936499	3.70238400
H	-7.54825115	-1.81753004	1.97667098
C	-10.42993832	2.23897505	-2.62379694
H	-11.99260426	2.57513094	-1.16502404
H	-8.76049995	1.65045702	-3.86221790
H	-11.52830982	0.61599600	3.66070509
C	-9.77756119	-0.53758699	4.19693518
H	-7.97677279	-1.71559203	4.38619900
H	-10.78175545	3.01875210	-3.30236006
H	-9.96133137	-0.47121301	5.27123117

Frequencies

Mode	IR frequency	IR intensity
1	3.82050000	0.69640000
2	6.09540000	0.34210000
3	7.65960000	0.76480000
4	10.78520000	0.09120000
5	12.24600000	0.18920000
6	14.37080000	0.17520000
7	15.50700000	0.04770000
8	18.86190000	0.35740000
9	24.70120000	0.17290000
10	26.06370000	0.09790000
11	28.56090000	0.05140000
12	36.06320000	0.44120000
13	40.07940000	0.80520000
14	40.35230000	2.01000000
15	40.95790000	0.06810000
16	42.89180000	2.31230000
17	43.97530000	0.20440000
18	46.08320000	0.45430000
19	52.00870000	0.12310000
20	52.65730000	0.97080000
21	55.06840000	0.06830000
22	56.32970000	0.57590000
23	61.15410000	0.34810000
24	61.51780000	0.05290000
25	64.17480000	1.24700000
26	66.53080000	0.97160000
27	71.41720000	0.19980000
28	73.37370000	0.11350000
29	75.10850000	1.65160000
30	78.77750000	0.34620000
31	80.78180000	0.22630000
32	83.55260000	0.74800000
33	90.36010000	0.27790000
34	90.40830000	0.15000000
35	94.69730000	32.66760000
36	97.34540000	0.11380000
37	99.81260000	2.91660000
38	102.68850000	6.31790000
39	109.54410000	6.76530000
40	116.13720000	0.41880000
41	116.64260000	0.37180000
42	121.63260000	0.64280000
43	124.49850000	5.05530000
44	132.18350000	19.66910000
45	137.73780000	1.31510000
46	140.53950000	1.47490000
47	142.16980000	13.14190000
48	143.66570000	2.39900000

49	149.84540000	1.85510000
50	153.49920000	1.45330000
51	161.21490000	1.62890000
52	162.89370000	2.76660000
53	163.67200000	0.70690000
54	167.38410000	18.00220000
55	168.77750000	5.40360000
56	172.02310000	4.88550000
57	173.29830000	2.40300000
58	176.06970000	4.88050000
59	183.46760000	31.01710000
60	192.09880000	3.99510000
61	197.23760000	13.43490000
62	205.08270000	2.94150000
63	211.41330000	69.33400000
64	216.64670000	3.41080000
65	217.58070000	0.34740000
66	222.08450000	0.39480000
67	225.96770000	0.33590000
68	230.64700000	229.90240000
69	234.26170000	0.24740000
70	235.40210000	0.05040000
71	240.45250000	6.20560000
72	245.38890000	2.38230000
73	254.09380000	0.21860000
74	258.35320000	0.39330000
75	259.98490000	0.05580000
76	260.14010000	0.11640000
77	260.68430000	0.04930000
78	261.63950000	0.10060000
79	282.44610000	0.70250000
80	285.57310000	7.20690000
81	298.52920000	28.84010000
82	303.02650000	11.16440000
83	305.69230000	0.92650000
84	314.50770000	1.37610000
85	329.54100000	123.86020000
86	336.06690000	0.55960000
87	337.43510000	0.62480000
88	340.01560000	43.39900000
89	340.96750000	7.92130000
90	344.14670000	14.89660000
91	351.72220000	13.12630000
92	364.52260000	77.31480000
93	372.51740000	3.38790000
94	375.92950000	71.19720000
95	378.01130000	17.31010000
96	383.26060000	86.57130000
97	387.37210000	39.23350000
98	389.36530000	109.84860000
99	405.47650000	2.15690000

100	410.76210000	40.73060000
101	411.40130000	24.24600000
102	411.92970000	49.53440000
103	414.39540000	15.64960000
104	415.31760000	17.93980000
105	421.46400000	72.83800000
106	423.08540000	3.88270000
107	428.89330000	84.93700000
108	441.86700000	39.55920000
109	450.94130000	3.75300000
110	453.60800000	4.01490000
111	461.50500000	4.37640000
112	461.72480000	85.27300000
113	467.23050000	4.73230000
114	484.98130000	18.10040000
115	504.13970000	92.25170000
116	507.83180000	36.66370000
117	516.58160000	12.30630000
118	520.01660000	349.15330000
119	524.07320000	50.72300000
120	535.77260000	5.62690000
121	548.70030000	21.93750000
122	552.38240000	0.10520000
123	555.64560000	5.12600000
124	565.21040000	11.55540000
125	577.28770000	13.14050000
126	580.34990000	0.37520000
127	586.62600000	122.18630000
128	592.76910000	29.38280000
129	607.35900000	268.51460000
130	616.09470000	4.89930000
131	630.68890000	272.24730000
132	641.19250000	8.12020000
133	661.71710000	12.15990000
134	665.21910000	15.75560000
135	692.56500000	35.33140000
136	693.67780000	11.93550000
137	694.75830000	24.47680000
138	702.27660000	6.49420000
139	704.90800000	20.35980000
140	715.86880000	2.33750000
141	720.97710000	14.56920000
142	723.58560000	19.57480000
143	725.07340000	18.88200000
144	728.76630000	16.09420000
145	737.85970000	4.17880000
146	740.93120000	2.07740000
147	743.42660000	3.42040000
148	755.13150000	19.16510000
149	756.47460000	9.78800000
150	756.85520000	8.04230000

151	756.98820000	16.18860000
152	757.32060000	4.63850000
153	760.77120000	301.97610000
154	763.66950000	7.47100000
155	764.56340000	0.36290000
156	765.38100000	4.36540000
157	765.68090000	8.53360000
158	768.18380000	35.12220000
159	770.38990000	22.82340000
160	775.63360000	58.23960000
161	779.25430000	4.52540000
162	788.06710000	76.61560000
163	789.84390000	5.90630000
164	792.45700000	46.58430000
165	793.10400000	0.96830000
166	794.44670000	27.37750000
167	799.52790000	24.71770000
168	800.28720000	30.87540000
169	840.59430000	18.78500000
170	851.62720000	5.90890000
171	853.26840000	1230.72000000
172	860.51970000	4.36400000
173	863.52310000	275.43920000
174	871.23260000	6.23470000
175	880.98010000	21.40510000
176	881.25170000	10.10400000
177	885.09830000	5.75100000
178	887.07220000	5.53210000
179	889.17110000	75.94450000
180	904.59930000	3.15370000
181	905.21080000	13.74990000
182	906.27430000	0.67120000
183	906.72160000	19.29090000
184	915.28440000	10.33420000
185	915.49200000	27.44010000
186	915.78410000	1.50800000
187	916.54320000	31.76680000
188	918.23880000	0.61690000
189	919.65150000	110.25320000
190	920.35800000	760.35580000
191	946.44460000	12.98900000
192	966.09670000	3.48010000
193	966.25380000	19.71360000
194	969.60530000	182.83220000
195	990.70860000	2.92970000
196	1010.24740000	2.33180000
197	1011.59420000	3.22000000
198	1011.77230000	0.10140000
199	1013.25610000	0.19540000
200	1027.50640000	148.97380000
201	1028.47250000	49.73380000

202	1029.38780000	95.52510000
203	1037.92300000	7.88310000
204	1038.74750000	22.04030000
205	1042.29790000	601.45080000
206	1052.28230000	6.42150000
207	1052.44870000	4.56010000
208	1053.51970000	6.33560000
209	1054.28630000	3.29630000
210	1054.48240000	0.95460000
211	1054.86500000	1.66410000
212	1060.35130000	184.17750000
213	1074.50410000	3.81350000
214	1075.93890000	2.97290000
215	1076.20530000	2.84480000
216	1076.63910000	52.24340000
217	1076.70220000	26.34140000
218	1079.47670000	20.52620000
219	1080.46130000	3.94650000
220	1087.10940000	116.89250000
221	1089.66390000	5.63180000
222	1089.71470000	2.81320000
223	1090.36340000	1.00250000
224	1090.91700000	0.42820000
225	1092.78410000	4.15820000
226	1094.43240000	3.06830000
227	1096.66230000	4.18330000
228	1116.61880000	3.06920000
229	1117.10460000	1.72940000
230	1117.38340000	44.27280000
231	1117.65150000	18.64950000
232	1120.99190000	120.80110000
233	1123.52650000	213.75680000
234	1128.86820000	16.24060000
235	1140.54370000	1.70920000
236	1142.18970000	0.29260000
237	1152.11720000	122.45040000
238	1152.73230000	22.12670000
239	1156.67090000	0.24690000
240	1159.01480000	40.03200000
241	1172.63100000	5.22220000
242	1177.05070000	265.24080000
243	1177.74360000	1949.92050000
244	1184.66370000	150.38600000
245	1212.85520000	170.72880000
246	1213.76090000	0.06620000
247	1215.53030000	6.63200000
248	1216.05630000	5.09820000
249	1217.63720000	11.94900000
250	1218.70560000	5.86100000
251	1219.31610000	1.85440000
252	1226.04280000	135.92120000

253	1227.95920000	72.31270000
254	1229.56550000	7.01440000
255	1231.33850000	2.43140000
256	1232.48020000	0.19230000
257	1234.17420000	4.84150000
258	1249.45550000	225.06460000
259	1250.90400000	204.04610000
260	1251.07940000	125.55970000
261	1256.07810000	209.80110000
262	1266.30060000	1278.71850000
263	1268.23780000	3805.64620000
264	1276.44440000	3.71210000
265	1291.52260000	1.97150000
266	1291.81740000	53.57930000
267	1292.26560000	2.39880000
268	1294.41710000	2.72350000
269	1296.47900000	23.45520000
270	1296.94770000	12.75000000
271	1300.09010000	5.33280000
272	1307.13480000	43.42510000
273	1314.13020000	1172.33980000
274	1318.13320000	4.21270000
275	1319.46410000	12.58490000
276	1320.54570000	15.50230000
277	1320.67280000	12.66990000
278	1322.83750000	610.06110000
279	1323.02070000	395.72050000
280	1323.39310000	226.28830000
281	1323.92270000	337.11770000
282	1324.66950000	159.53290000
283	1325.17850000	0.73800000
284	1325.35510000	89.63760000
285	1326.87830000	26.51100000
286	1329.53870000	22.21070000
287	1332.44260000	41.79960000
288	1345.91590000	423.36010000
289	1368.47400000	666.06350000
290	1373.06510000	4886.31110000
291	1375.65740000	96.92990000
292	1381.67590000	0.26870000
293	1382.01590000	0.19620000
294	1382.21690000	0.04000000
295	1383.16300000	0.71890000
296	1384.35750000	0.25500000
297	1385.13800000	1.78710000
298	1392.73230000	356.00010000
299	1396.90710000	234.48600000
300	1399.10820000	344.32800000
301	1399.26460000	1.41400000
302	1399.56860000	597.35020000
303	1399.77790000	1.17740000

304	1400.01840000	189.85850000
305	1401.93380000	22.78060000
306	1402.02540000	7.35370000
307	1402.38050000	28.23030000
308	1409.21640000	102.23210000
309	1419.51030000	36.98330000
310	1420.49000000	124.26710000
311	1420.99470000	252.39970000
312	1422.09310000	1352.66930000
313	1422.57070000	80.24460000
314	1425.69080000	20.84310000
315	1430.73550000	14.23600000
316	1439.97140000	223.47080000
317	1447.57630000	7.45300000
318	1447.67450000	6.45260000
319	1449.18050000	17.01050000
320	1449.31250000	4.28680000
321	1449.88400000	1.55550000
322	1451.80390000	3.81690000
323	1452.13630000	153.06050000
324	1453.00860000	258.20520000
325	1456.44700000	17.96760000
326	1457.54710000	37.35260000
327	1457.71280000	9.73750000
328	1457.80910000	9.14790000
329	1458.68110000	10.48960000
330	1459.21100000	6.56950000
331	1459.46810000	9.50880000
332	1460.00900000	5.35940000
333	1460.17160000	4.27050000
334	1460.81900000	13.47680000
335	1461.83800000	129.53170000
336	1465.32400000	0.30650000
337	1465.62000000	0.26620000
338	1465.83140000	0.12660000
339	1466.12060000	0.08070000
340	1467.34270000	51.27720000
341	1473.00030000	4.08270000
342	1473.92770000	8.54980000
343	1476.47820000	17.91750000
344	1477.04400000	15.71350000
345	1477.39340000	17.09670000
346	1478.44630000	14.91850000
347	1481.66990000	9.21810000
348	1484.74320000	22.88030000
349	1485.84410000	23.15360000
350	1492.33320000	222.60430000
351	1514.76530000	1493.99280000
352	1514.87710000	735.01470000
353	1543.57580000	327.46140000
354	1543.87230000	538.37570000

355	1545.23260000	32.86510000
356	1561.58390000	9516.04140000
357	1600.93570000	2761.07080000
358	1623.05560000	6.75160000
359	1645.02700000	0.42620000
360	1657.08410000	64.29800000
361	1658.71610000	1224.13510000
362	1662.06200000	2.50520000
363	1680.76040000	1078.87010000
364	1684.69740000	124.69980000
365	1713.83710000	966.84600000
366	1732.08270000	1970.63700000
367	1757.16360000	1532.78630000
368	1774.35110000	2742.06290000
369	2036.07250000	23993.49420000
370	2163.93870000	3576.69470000
371	3044.37640000	34.12990000
372	3045.66170000	42.66650000
373	3045.70440000	26.19440000
374	3045.82120000	35.66180000
375	3046.75370000	39.26540000
376	3047.17210000	32.56480000
377	3048.29890000	15.06130000
378	3049.00670000	29.92810000
379	3049.31380000	24.61360000
380	3049.38400000	40.15300000
381	3049.45250000	11.02030000
382	3049.84120000	34.73230000
383	3056.55080000	27.80650000
384	3059.96510000	22.82190000
385	3060.83480000	21.52560000
386	3061.17970000	41.42960000
387	3061.30910000	27.57580000
388	3063.54300000	17.89040000
389	3065.64330000	26.55380000
390	3066.53960000	47.56280000
391	3067.85360000	25.34410000
392	3067.89890000	27.59710000
393	3068.83390000	25.05700000
394	3070.89360000	29.73190000
395	3071.88600000	12.85410000
396	3081.39270000	68.47580000
397	3081.60300000	26.19560000
398	3089.82630000	14.42710000
399	3090.86800000	8.34030000
400	3090.90030000	11.26890000
401	3091.18800000	10.85400000
402	3094.58090000	5.25180000
403	3096.76920000	2.48850000
404	3115.30970000	4.47940000
405	3116.07510000	7.63380000

406	3116.97990000	39.17240000
407	3119.03970000	11.67140000
408	3119.39140000	9.59620000
409	3120.77010000	11.09680000
410	3121.73540000	13.71070000
411	3131.80450000	14.64350000
412	3132.01650000	3.97870000
413	3134.86550000	6.61960000
414	3135.71370000	4.47200000
415	3137.52160000	20.36820000
416	3137.63320000	1.43830000
417	3137.89330000	58.41280000
418	3139.67120000	61.35430000
419	3139.71070000	56.16780000
420	3139.89350000	47.90310000
421	3140.02880000	47.33080000
422	3140.15490000	55.62780000
423	3146.33400000	28.96700000
424	3147.91650000	27.69540000
425	3148.46330000	34.81340000
426	3148.52250000	30.49540000
427	3148.55060000	32.60620000
428	3148.65390000	29.94910000
429	3169.12040000	12.02920000
430	3172.45260000	10.99180000
431	3200.26810000	44.10620000
432	3210.27630000	0.48340000
433	3210.38920000	2.54890000
434	3214.96670000	4.12800000
435	3218.18970000	4.22840000
436	3218.40460000	4.17130000
437	3224.65450000	39.46150000
438	3228.59880000	77.39650000
439	3230.35380000	1.68630000
440	3230.37530000	2.57280000
441	3233.33950000	21.14020000
442	3233.46640000	5.59150000
443	3234.76680000	6.33300000
444	3241.93860000	2.62900000
445	3242.81430000	5.11610000
446	3243.65660000	1.89560000
447	3247.34690000	21.53430000

6 Crystallographic data

Identification code	NDI-Pt-PTZ
Empirical formula	$C_{77}H_{105}N_3O_4P_2PtS$
Formula weight	1425.72
Temperature/K	100
Crystal system	triclinic
Space group	P-1
a/Å	9.6512(8)
b/Å	19.5530(12)
c/Å	20.0708(7)
α/°	71.675(4)
β/°	89.523(4)
γ/°	88.863(6)
Volume/Å^3	3594.8(4)
Z	2
ρ_{calc}g/cm^3	1.317
μ/mm^{-1}	1.911
F(000)	1488.0
Crystal size/mm^3	0.119 × 0.017 × 0.0085
Radiation	Synchrotron (λ = 0.6889 Å)
2Θ range for data collection/°	3.404 to 40.29
Index ranges	$-7 \leq h \leq 9$, $-19 \leq k \leq 19$, $-20 \leq l \leq 20$
Reflections collected	22160
Independent reflections	7084 [R_{int} = 0.1700, R_{sigma} = 0.1098]
Data/restraints/parameters	7084/678/800
Goodness-of-fit on F^2	1.098
Final R indexes [I>=2σ (I)]	R_1 = 0.1078, wR_2 = 0.3157
Final R indexes [all data]	R_1 = 0.1246, wR_2 = 0.3265
Largest diff. peak/hole / e Å^{-3}	3.90/-1.86

Table S2. Crystal data and structure refinement for NDI-Pt-PTZ.

**Atom**	***x***	***y***	***z***	**U(eq)**
Pt1	5489.3(12)	2632.2(5)	3222.8(5)	48.9(5)
S1	14265(8)	-713(3)	1256(3)	53.7(18)
P1	5853(8)	3506(4)	2174(3)	51.5(18)
P2	5255(8)	1688(3)	4228(3)	50.5(18)
O1	5530(16)	5047(8)	3421(7)	41(4)
O2	4592(16)	6915(8)	4254(7)	39(4)
O3	454(18)	3006(8)	5763(7)	47(4)
O4	-439(19)	4922(8)	6524(8)	57(5)
N1	5090(20)	5967(9)	3861(9)	43(4)
N2	-40(20)	3969(10)	6125(9)	43(4)
N3	11140(20)	-413(10)	1264(10)	46(4)
C1	4600(30)	3329(12)	3619(13)	48(6)
C2	4160(30)	3751(12)	3930(12)	44(6)
C3	3510(20)	4151(11)	4322(11)	36(5)
C4	3770(20)	4838(11)	4312(10)	30(4)
C5	4850(20)	5278(11)	3830(11)	35(5)
C6	4360(20)	6308(11)	4284(10)	31(4)
C7	3320(20)	5861(11)	4754(10)	34(4)
C8	3060(20)	5163(11)	4742(10)	32(4)
C9	2630(30)	6154(13)	5216(12)	49(6)
C10	1600(30)	5745(12)	5682(12)	45(6)
C11	1330(30)	5073(12)	5675(11)	42(5)
C12	1990(30)	4754(12)	5224(12)	43(5)
C13	220(20)	4670(12)	6134(11)	37(5)
C14	630(30)	3635(12)	5728(11)	40(5)
C15	1750(20)	4060(11)	5226(10)	33(4)
C16	2450(20)	3753(12)	4783(11)	41(5)
C17	6050(30)	6411(12)	3338(11)	44(5)
C18	5360(30)	6815(13)	2645(12)	50(6)
C19	6390(30)	7265(14)	2101(13)	57(6)
C20	7230(30)	6855(15)	1741(14)	64(6)
C21	6650(30)	6576(14)	1229(13)	59(6)
C22	7510(40)	6121(18)	917(16)	84(8)
C23	6990(50)	5860(19)	378(17)	103(10)
C24	8070(60)	5430(30)	80(20)	158(18)
C25	-1080(30)	3558(12)	6617(11)	43(5)
C26	-510(30)	3182(12)	7364(11)	44(5)
C27	-1680(30)	2894(12)	7870(11)	44(5)
C28	-1290(30)	2523(13)	8621(12)	52(6)
C29	-2560(30)	2358(16)	9089(13)	68(7)
C30	-2280(40)	1951(18)	9874(15)	84(8)
C31	-1410(40)	2306(19)	10210(16)	91(8)
C32	-1870(40)	3060(20)	10180(20)	111(12)
C33	6300(30)	1923(14)	2801(15)	63(7)
C34	6880(30)	1523(13)	2498(13)	52(6)
C35	7600(30)	1053(12)	2194(12)	47(5)
C36	6820(30)	718(12)	1761(12)	48(5)
C37	7530(30)	263(13)	1461(13)	49(5)
C38	8960(30)	150(12)	1538(12)	43(5)
C39	9630(30)	516(12)	1915(12)	48(5)

C40	8950(30)	981(12)	2194(12)	46(5)
C41	9610(30)	-351(12)	1203(12)	43(4)
C42	11980(30)	166(12)	809(11)	41(4)
C43	11290(30)	782(12)	379(12)	53(5)
C44	12090(30)	1327(14)	-105(13)	56(6)
C45	13530(30)	1270(13)	-118(13)	55(6)
C46	14160(30)	655(12)	327(12)	50(5)
C47	13380(30)	95(12)	782(13)	49(5)
C48	11740(30)	-840(12)	1897(12)	42(4)
C49	10820(30)	-1150(12)	2502(12)	46(5)
C50	11420(30)	-1587(12)	3126(13)	52(5)
C51	12870(30)	-1718(13)	3198(14)	58(6)
C52	13670(30)	-1412(12)	2622(13)	53(5)
C53	13120(30)	-995(11)	1991(12)	43(4)
C54	5190(30)	4393(14)	2070(14)	61(6)
C55	3700(30)	4472(13)	2007(13)	56(6)
C56	3090(30)	5248(15)	1924(18)	79(8)
C57	1510(30)	5287(14)	1794(16)	71(7)
C58	5180(30)	3251(13)	1469(12)	54(5)
C59	3720(30)	2933(14)	1559(13)	58(6)
C60	3500(30)	2373(16)	1275(16)	75(8)
C61	2070(40)	2090(19)	1340(20)	98(11)
C62	7740(30)	3546(17)	1993(15)	72(6)
C63	8660(40)	3560(20)	2617(17)	97(10)
C64	10090(40)	3270(20)	2630(19)	96(9)
C65	10980(40)	3290(20)	3250(20)	105(11)
C66	4250(30)	1874(13)	4921(13)	54(6)
C67	4020(30)	1266(13)	5620(13)	61(7)
C68	3130(40)	1519(15)	6104(15)	77(9)
C69	2840(40)	922(17)	6773(17)	99(12)
C70	4370(30)	945(13)	4041(14)	60(6)
C71	3080(40)	1159(16)	3696(19)	86(9)
C72	2330(40)	560(18)	3500(20)	98(9)
C73	3130(40)	302(18)	2990(20)	98(10)
C74	6900(30)	1319(13)	4565(14)	57(5)
C75	7840(30)	1847(15)	4743(15)	65(6)
C76	9330(40)	1560(20)	4970(30)	137(16)
C77	9920(50)	940(20)	5270(30)	180(20)

Table S3. Fractional atomic coordinates (× 10^4) and equivalent isotropic displacement parameters ($Å^2 \times 10^4$) for NDI-Pt-PTZ. U_{eq} is defined as 1/3 of the trace of the orthogonalised U_{IJ} tensor.

Atom	U_{11}	U_{22}	U_{33}	U_{23}	U_{13}	U_{12}
Pt1	66.3(9)	43.7(6)	39.7(6)	-17.8(5)	8.1(5)	2.7(5)
S1	61(5)	45(3)	50(4)	-9(3)	17(3)	-5(3)
P1	60(5)	53(4)	43(4)	-17(3)	7(3)	-6(3)
P2	70(5)	36(3)	46(4)	-14(3)	-2(3)	2(3)
O1	41(9)	44(8)	35(7)	-10(6)	8(6)	-3(6)
O2	43(10)	39(6)	33(8)	-10(5)	-1(7)	-3(6)
O3	66(11)	42(6)	32(8)	-11(6)	22(8)	-16(6)
O4	81(11)	44(8)	44(9)	-12(7)	31(8)	-7(7)
N1	52(9)	40(6)	34(7)	-10(5)	10(7)	-5(5)
N2	49(9)	44(6)	36(7)	-12(5)	8(7)	-4(6)
N3	55(7)	39(7)	45(7)	-14(5)	9(5)	-8(5)
C1	52(15)	42(8)	51(10)	-17(8)	2(9)	4(8)
C2	53(13)	34(9)	44(11)	-11(8)	16(10)	-8(8)
C3	41(10)	36(6)	28(8)	-7(6)	-1(7)	3(6)
C4	32(8)	35(6)	18(7)	-4(5)	-8(6)	6(5)
C5	37(9)	40(6)	26(8)	-6(6)	-2(7)	2(6)
C6	33(9)	37(6)	19(7)	-3(5)	-12(6)	5(6)
C7	38(9)	36(6)	25(8)	-6(5)	-4(7)	2(6)
C8	33(9)	36(6)	25(8)	-7(5)	-5(7)	4(5)
C9	62(12)	40(8)	49(10)	-18(8)	19(10)	-10(8)
C10	57(12)	43(7)	37(10)	-16(7)	13(9)	-9(7)
C11	54(10)	39(7)	32(9)	-12(6)	11(8)	-5(6)
C12	53(10)	41(6)	37(9)	-15(6)	11(8)	-7(6)
C13	44(10)	42(6)	22(8)	-6(6)	0(7)	0(6)
C14	44(10)	41(6)	33(9)	-11(6)	5(8)	-6(6)
C15	36(9)	35(6)	25(8)	-6(5)	-3(7)	1(6)
C16	48(10)	40(8)	34(9)	-11(7)	5(8)	-2(7)
C17	51(10)	43(9)	34(7)	-9(6)	8(7)	-5(7)
C18	55(12)	52(11)	36(7)	-5(7)	2(7)	-12(9)
C19	58(13)	63(11)	39(9)	1(7)	1(8)	-13(9)
C20	59(13)	73(14)	51(11)	-8(10)	6(9)	-11(10)
C21	64(15)	61(13)	41(11)	2(10)	13(10)	-21(11)
C22	97(19)	89(17)	58(14)	-15(13)	9(12)	6(14)
C23	160(30)	85(19)	63(15)	-18(15)	0(15)	3(17)
C24	220(40)	170(40)	100(30)	-60(30)	-10(20)	60(30)
C25	40(10)	47(9)	36(7)	-6(6)	5(6)	3(7)
C26	47(11)	43(11)	36(7)	-5(7)	3(6)	-1(9)
C27	55(12)	40(11)	35(7)	-10(7)	7(7)	-10(9)
C28	71(13)	46(12)	36(7)	-7(7)	4(7)	-19(10)
C29	80(14)	70(16)	48(8)	-8(8)	17(8)	-23(11)
C30	100(20)	91(15)	48(9)	-6(9)	14(9)	-26(13)
C31	110(20)	106(17)	57(13)	-19(12)	14(13)	-29(15)
C32	130(30)	113(17)	100(30)	-38(16)	10(20)	-23(16)
C33	70(17)	57(9)	70(11)	-31(8)	11(11)	1(9)
C34	59(12)	51(11)	53(13)	-25(11)	5(10)	-3(9)
C35	56(9)	42(10)	45(11)	-17(9)	9(7)	-6(7)
C36	59(9)	45(11)	42(11)	-16(10)	3(8)	-1(7)
C37	51(8)	48(11)	54(12)	-24(10)	10(7)	-11(7)
C38	51(8)	38(9)	41(11)	-14(9)	9(7)	-11(7)

C39	52(9)	47(11)	50(12)	-23(10)	10(8)	-11(7)
C40	56(9)	45(10)	41(12)	-18(10)	7(8)	-7(7)
C41	53(7)	39(9)	37(10)	-12(8)	9(6)	-9(6)
C42	55(7)	38(7)	35(8)	-17(6)	10(6)	-8(5)
C43	66(10)	44(8)	46(10)	-12(7)	11(7)	-2(6)
C44	69(10)	51(9)	45(11)	-7(8)	8(8)	-8(7)
C45	69(10)	49(9)	47(11)	-15(8)	6(8)	-9(7)
C46	55(9)	46(8)	48(10)	-13(7)	10(7)	-14(7)
C47	56(7)	42(7)	50(9)	-16(6)	12(6)	-9(5)
C48	52(7)	36(9)	40(6)	-14(6)	12(5)	-5(6)
C49	57(9)	37(10)	45(7)	-15(6)	16(6)	-7(7)
C50	68(10)	39(11)	50(8)	-14(7)	10(7)	-7(8)
C51	68(10)	47(12)	54(8)	-10(8)	9(7)	-3(9)
C52	61(10)	43(11)	52(7)	-12(7)	6(6)	-6(8)
C53	51(7)	30(9)	49(7)	-16(6)	12(5)	-7(6)
C54	78(11)	55(8)	53(13)	-23(8)	8(9)	-8(7)
C55	79(11)	49(10)	43(14)	-21(10)	10(9)	-6(8)
C56	91(13)	54(11)	100(20)	-34(12)	9(12)	-1(9)
C57	90(13)	42(13)	80(20)	-20(13)	13(12)	-5(10)
C58	76(11)	48(11)	36(8)	-10(8)	7(7)	-9(8)
C59	74(11)	53(12)	46(13)	-13(11)	8(9)	-10(9)
C60	94(16)	64(13)	73(18)	-29(13)	10(12)	-16(11)
C61	99(17)	100(20)	110(30)	-60(20)	20(15)	-35(15)
C62	70(8)	88(15)	71(12)	-41(11)	20(7)	-22(7)
C63	76(11)	150(30)	76(15)	-53(16)	18(10)	-30(12)
C64	71(12)	130(20)	99(18)	-49(16)	19(11)	-39(13)
C65	93(18)	120(30)	120(20)	-59(19)	-5(15)	-15(17)
C66	72(13)	39(9)	50(8)	-12(7)	7(8)	-3(8)
C67	83(18)	47(11)	51(9)	-10(7)	3(9)	-18(10)
C68	110(20)	55(12)	60(11)	-10(9)	22(12)	-14(12)
C69	140(30)	67(16)	71(12)	2(11)	35(16)	-8(17)
C70	76(12)	47(9)	58(13)	-20(9)	-6(9)	-5(8)
C71	89(13)	67(13)	110(20)	-43(13)	-35(13)	4(10)
C72	108(17)	72(16)	120(20)	-45(16)	-40(14)	-4(12)
C73	110(20)	80(20)	130(20)	-53(18)	-41(18)	-4(17)
C74	71(8)	42(10)	59(12)	-16(9)	1(7)	-5(7)
C75	82(12)	62(12)	55(15)	-26(12)	12(10)	-22(9)
C76	98(14)	94(18)	240(40)	-70(20)	-44(18)	-2(13)
C77	150(30)	97(19)	310(60)	-90(30)	-100(30)	10(18)

Table S4. Anisotropy displacement parameters $(\text{Å}^2 \times 10^3)$ for NDI-Pt-PTZ. The anisotropy displacement factor exponent takes the form: $-2\pi^2[h^2a^{*2}U_{11} + 2hka^*b^*U_{12} + \ldots]$.

Atom	Atom	Length/Å	Atom	Atom	Length/Å
Pt1	P1	2.285(7)	C23	C24	1.56(5)
Pt1	P2	2.280(7)	C25	C26	1.55(3)
Pt1	C1	1.96(2)	C26	C27	1.50(3)
Pt1	C33	1.98(3)	C27	C28	1.50(3)
S1	C47	1.77(3)	C28	C29	1.51(4)
S1	C53	1.79(2)	C29	C30	1.55(4)
P1	C54	1.79(3)	C30	C31	1.40(4)
P1	C58	1.78(2)	C31	C32	1.51(5)
P1	C62	1.86(3)	C33	C34	1.25(3)
P2	C66	1.81(3)	C34	*C35*	1.42(3)
P2	C70	1.84(2)	C35	*C36*	1.46(3)
P2	C74	1.78(3)	C35	*C40*	1.31(3)
O1	C5	1.23(2)	C36	*C37*	1.39(3)
O2	C6	1.19(2)	C37	*C38*	1.40(3)
O3	C14	1.22(2)	C38	*C39*	1.36(3)
O4	C13	1.22(3)	C38	*C41*	1.48(3)
N1	C5	1.39(3)	C39	*C40*	1.36(3)
N1	C6	1.41(3)	C42	*C43*	1.40(3)
N1	C17	1.47(3)	C42	*C47*	1.36(3)
N2	C13	1.40(3)	C43	*C44*	1.43(3)
N2	C14	1.34(3)	C44	*C45*	1.39(4)
N2	C25	1.46(3)	C45	*C46*	1.39(4)
N3	C41	1.48(3)	C46	*C47*	1.42(3)
N3	C42	1.47(3)	C48	*C49*	1.47(3)
N3	C48	1.40(3)	C48	*C53*	1.37(3)
C1	C2	1.25(3)	C49	*C50*	1.40(3)
C2	C3	1.41(3)	C50	*C51*	1.42(4)
C3	C4	1.37(3)	C51	*C52*	1.36(4)
C3	C16	1.44(3)	C52	*C53*	1.38(3)
C4	C5	1.50(3)	C54	*C55*	1.44(4)
C4	C8	1.39(3)	C55	*C56*	1.57(4)
C6	C7	1.47(3)	C56	*C57*	1.55(4)
C7	C8	1.40(3)	C58	*C59*	1.53(4)
C7	C9	1.40(3)	C59	*C60*	1.41(4)
C8	C12	1.47(3)	C60	*C61*	1.48(4)
C9	C10	1.43(3)	C62	*C63*	1.55(4)
C10	C11	1.35(3)	C63	*C64*	1.47(5)
C11	C12	1.39(3)	C64	*C65*	1.52(5)
C11	C13	1.48(3)	C66	*C67*	1.55(3)
C12	C15	1.38(3)	C67	*C68*	1.48(4)
C14	C15	1.54(3)	C68	*C69*	1.51(4)
C15	C16	1.39(3)	C70	*C71*	1.42(4)
C17	C18	1.52(3)	C71	*C72*	1.55(4)
C18	C19	1.54(3)	C72	*C73*	1.47(5)
C19	C20	1.47(4)	C74	*C75*	1.52(3)
C20	C21	1.43(4)	C75	*C76*	1.55(5)
C21	C22	1.48(4)	C76	*C77*	1.30(5)
C22	C23	1.43(4)			

Table S5. Bond lengths for NDI-Pt-PTZ.

Atom	Atom	Atom	Angle/°	Atom	Atom	Atom	Angle/°
P2	Pt1	P1	174.5(2)	N1	C17	C18	113(2)
C1	Pt1	P1	91.5(7)	C17	C18	C19	112(2)
C1	Pt1	P2	93.9(7)	C20	C19	C18	115(2)
C1	Pt1	C33	177.3(12)	C21	C20	C19	121(3)
C33	Pt1	P1	88.1(8)	C20	C21	C22	120(3)
C33	Pt1	P2	86.5(8)	C23	C22	C21	122(3)
C47	S1	C53	98.3(12)	C22	C23	C24	115(4)
C54	P1	Pt1	118.0(9)	N2	C25	C26	114(2)
C54	P1	C62	109.5(14)	C27	C26	C25	111(2)
C58	P1	Pt1	110.9(8)	C28	C27	C26	117(2)
C58	P1	C54	106.4(13)	C27	C28	C29	111(2)
C58	P1	C62	102.8(13)	C28	C29	C30	116(3)
C62	P1	Pt1	108.1(11)	C31	C30	C29	115(3)
C66	P2	Pt1	115.7(8)	C30	C31	C32	116(3)
C66	P2	C70	105.4(13)	C34	C33	Pt1	175(2)
C70	P2	Pt1	110.1(9)	C33	C34	C35	176(3)
C74	P2	Pt1	111.0(9)	C34	C35	C36	119(2)
C74	P2	C66	109.2(13)	C40	C35	C34	124(2)
C74	P2	C70	104.7(12)	C40	C35	C36	117(2)
C5	N1	C6	125.8(19)	C37	C36	C35	118(2)
C5	N1	C17	116.7(18)	C36	C37	C38	121(2)
C6	N1	C17	116.9(17)	C37	C38	C41	117(2)
C13	N2	C25	117.6(18)	C39	C38	C37	117(2)
C14	N2	C13	125(2)	C39	C38	C41	126(2)
C14	N2	C25	117.3(19)	C40	C39	C38	123(3)
C42	N3	C41	118.9(19)	C35	C40	C39	123(2)
C48	N3	C41	119.2(19)	C38	C41	N3	114.5(19)
C48	N3	C42	117(2)	C43	C42	N3	118(2)
C2	C1	Pt1	173(2)	C47	C42	N3	121(2)
C1	C2	C3	172(2)	C47	C42	C43	121(2)
C2	C3	C16	112.6(19)	C42	C43	C44	118(3)
C4	C3	C2	129(2)	C45	C44	C43	121(3)
C4	C3	C16	119(2)	C46	C45	C44	118(2)
C3	C4	C5	120.7(19)	C45	C46	C47	121(3)
C3	C4	C8	122(2)	C42	C47	S1	122.0(18)
C8	C4	C5	116.9(18)	C42	C47	C46	120(2)
O1	C5	N1	120(2)	C46	C47	S1	118(2)
O1	C5	C4	122.5(19)	N3	C48	C49	118(2)
N1	C5	C4	117.9(19)	C53	C48	N3	124(2)
O2	C6	N1	121(2)	C53	C48	C49	117(2)
O2	C6	C7	124(2)	C50	C49	C48	118(2)
N1	C6	C7	114.8(18)	C49	C50	C51	123(2)
C8	C7	C6	120.8(19)	C52	C51	C50	117(3)
C9	C7	C6	117.5(19)	C51	C52	C53	122(3)
C9	C7	C8	122(2)	C48	C53	S1	119.4(19)
C4	C8	C7	124(2)	C48	C53	C52	123(2)
C4	C8	C12	118.9(19)	C52	C53	S1	118(2)
C7	C8	C12	117.4(19)	C55	C54	P1	115.0(18)
C7	C9	C10	120(2)	C54	C55	C56	116(2)

C11	C10	C9	119(2)	C57	C56	C55	111(2)
C10	C11	C12	123(2)	C59	C58	P1	117.4(18)
C10	C11	C13	119(2)	C60	C59	C58	117(2)
C12	C11	C13	118(2)	C59	C60	C61	116(3)
C11	C12	C8	118.6(19)	C63	C62	P1	115(2)
C15	C12	C8	118(2)	C64	C63	C62	117(3)
C15	C12	C11	123(2)	C63	C64	C65	116(3)
O4	C13	N2	120(2)	C67	C66	P2	119.6(18)
O4	C13	C11	122(2)	C68	C67	C66	111(2)
N2	C13	C11	118.1(19)	C67	C68	C69	112(2)
O3	C14	N2	124(2)	C71	C70	P2	112.9(19)
O3	C14	C15	118(2)	C70	C71	C72	115(3)
N2	C14	C15	117.3(19)	C73	C72	C71	113(3)
C12	C15	C14	117.8(19)	C75	C74	P2	114.3(19)
C12	C15	C16	122(2)	C74	C75	C76	115(2)
C16	C15	C14	120.2(19)	C77	C76	C75	136(4)
C15	C16	C3	120(2)				

Table S6. Bond angles for NDI-Pt-PTZ.

**Atom**	***x***	***y***	***z***	**U(eq)**
H9	2840	6625	5221	59
H10	1115	5944	5993	54
H16	2227	3282	4782	49
H17A	6805	6097	3251	52
H17B	6483	6764	3532	52
H18A	4623	7138	2730	60
H18B	4917	6464	2454	60
H19A	5859	7644	1743	69
H19B	7013	7508	2339	69
H20A	8007	7168	1509	77
H20B	7652	6441	2110	77
H21A	6299	6992	839	71
H21B	5830	6292	1447	71
H22A	8354	6396	728	100
H22B	7816	5694	1308	100
H23A	6646	6276	-13	123
H23B	6194	5547	568	123
H24A	7641	5273	-288	237
H24B	8397	5003	458	237
H24C	8863	5734	-118	237
H25A	-1456	3187	6430	51
H25B	-1855	3886	6647	51
H26A	122	2780	7356	53
H26B	18	3530	7522	53
H27A	-2318	3301	7855	53
H27B	-2199	2551	7699	53
H28A	-669	2834	8783	63
H28B	-783	2068	8656	63
H29A	-3195	2067	8907	82

H29B	-3048	2818	9055	82
H30A	-3174	1865	10126	101
H30B	-1853	1475	9912	101
H31A	-475	2332	9998	109
H31B	-1326	2010	10709	109
H32A	-2086	3342	9695	167
H32B	-1118	3291	10353	167
H32C	-2691	3035	10475	167
H36	5855	810	1687	58
H37	7028	22	1198	59
H39	10597	444	1985	57
H40	9488	1266	2397	56
H41A	9359	-188	699	51
H41B	9215	-835	1417	51
H43	10311	837	409	64
H44	11636	1734	-422	68
H45	14061	1643	-424	66
H46	15146	610	326	60
H49	9846	-1056	2471	55
H50	10840	-1804	3517	63
H51	13268	-2007	3629	69
H52	14649	-1490	2658	63
H54A	5479	4536	2478	73
H54B	5610	4731	1646	73
H55A	3280	4129	2429	67
H55B	3413	4333	1596	67
H56A	3287	5374	2354	95
H56B	3558	5603	1525	95
H57B	1204	5793	1629	106
H57A	1034	5036	2232	106
H57C	1303	5058	1438	106
H58A	5172	3681	1045	65
H58B	5828	2893	1376	65
H59A	3510	2753	2067	70
H59B	3050	3328	1342	70
H60A	4141	1969	1505	90
H60B	3739	2547	770	90
H61A	1432	2464	1062	147
H61B	1781	1950	1838	147
H61C	2047	1669	1179	147
H62A	8022	3122	1851	86
H62B	7926	3982	1591	86
H63A	8713	4068	2615	116
H63B	8194	3286	3058	116
H64A	10559	3551	2191	115
H64B	10039	2768	2631	115
H65A	11239	3788	3188	158
H65B	11820	2995	3263	158
H65C	10457	3099	3684	158
H66A	4705	2274	5034	65
H66B	3329	2056	4725	65
H67A	3575	855	5523	74
H67B	4926	1095	5845	74

H68A	2248	1714	5868	92
H68B	3603	1914	6219	92
H69A	1933	716	6736	148
H69B	2822	1115	7168	148
H69C	3559	546	6849	148
H70A	4978	747	3744	72
H70B	4210	555	4488	72
H71A	3243	1567	3264	104
H71B	2466	1336	4005	104
H72A	2167	150	3925	117
H72B	1417	748	3290	117
H73A	3143	681	2533	147
H73B	2687	-127	2938	147
H73C	4076	179	3159	147
H74A	6758	904	4993	69
H74B	7372	1134	4214	69
H75A	7409	1995	5125	78
H75B	7910	2282	4327	78
H76A	9876	1754	4535	165
H76B	9608	1851	5274	165
H77A	10916	1004	5321	265
H77B	9511	722	5737	265
H77C	9783	624	4985	265

Crystal structure determination of NDI-Pt-PTZ

Crystal Data for $C_{77}H_{105}N_3O_4P_2PtS$ (M =1425.72 g/mol): triclinic, space group P-1 (no. 2), a = 9.6512(8) Å, b = 19.5530(12) Å, c = 20.0708(7) Å, α = 71.675(4)°, β = 89.523(4)°, γ = 88.863(6)°, V = 3594.8(4) Å^3, Z = 2, T = 100 K, μ(?) = 1.911 mm^{-1}, $Dcalc$ = 1.317 g/cm^3, 22160 reflections measured (3.404° ≤ 2Θ ≤ 40.29°), 7084 unique (R_{int} = 0.1700, R_{sigma} = 0.1098) which were used in all calculations. The final R_1 was 0.1078 (I > 2σ(I)) and wR_2 was 0.3265 (all data).

Additional experimental details

Single crystals suitable for diffraction experiments were obtained by slow evaporation from DCM. Single crystal diffraction data was collected at beamline I19 at Diamond Light Source.[6–8] A single crystals of **NDI-Pt-PTZ (3)** was taken from the mother liquor and placed in fomblin oil on a glass slide. A crystal was picked and mounted on a MiTeGen 100 µm microloop. The single crystal, in turn, was placed directly onto the Newport diffractometer at beamline I19. All full hemisphere was collected using three 130 ° omega scans, two 120 ° phi scans and one 240 ° phi scan with a 0.4 ° increment. Data was collected on a Newport diffractometer equipped with a Pilatus 300 K detector and an Oxford Cryosystems Cryostream to maintain the sample temperature at 100 K. Data was collected at 0.6889(1) Å. The data were processed using CryAlisPro[9] using a predetermined instrument model determined from a strongly diffracting reference crystal to enable more accurate determination of the orientation matrix used for integration of diffraction intensities. Data were corrected for absorption using empirical methods (SADABS) based on symmetry-equivalent reflections and measurements at different azimuthal angles.[10–12] Structure solution was achieved by direct methods and the crystal structure was refined using full-matrix least-squares against weighted F^2 values using SHELXL[13] within Olex2.[14] Non-hydrogen atoms were refined anisotropically. Hydrogen atoms were placed in calculated positions, refined to idealized geometries (riding model) and assigned a fixed isotropic displacement parameter.

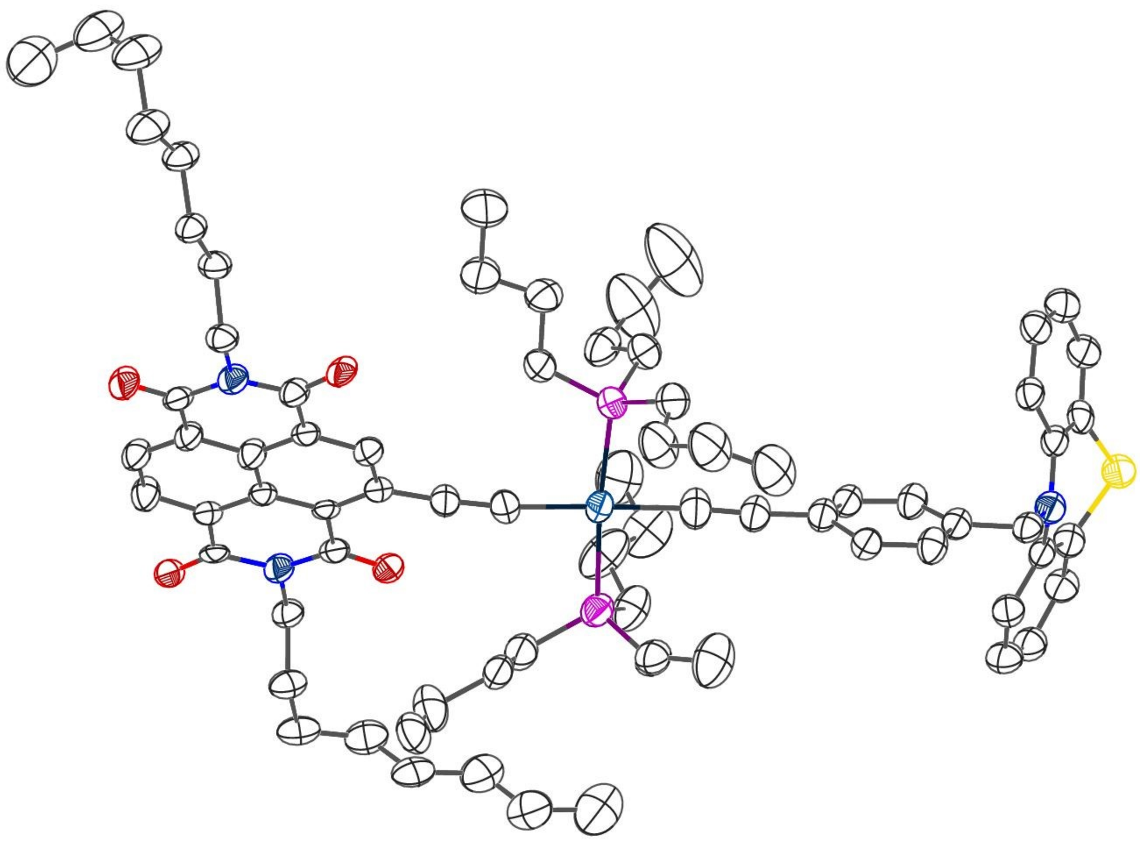

Fig.S18. The x-ray crystal structure of complex **3**, including the alkyl chains. The hydrogen atoms have been omitted for clarity.

7 References

1 K. Xiong and Y. Xiao, *Tetrahedron Lett.*, 2013, **54**, 3171–3175.

2 P. A. Scattergood, M. Delor, I. V. Sazanovich, O. V. Bouganov, S. A. Tikhomirov, A. S. Stasheuski, A. W. Parker, G. M. Greetham, M. Towrie, E. S. Davies, A. J. H. M. Meijer and J. A. Weinstein, *Dalt. Trans.*, 2014, **43**, 17677–17693.

3 Y. Liu, S. Jiang, K. Glusac, D. H. Powell, D. F. Anderson and K. S. Schanze, *J. Am. Chem. Soc.*, 2002, **124**, 12412–12413.

4 T. Cardolaccia, A. M. Funston, M. E. Kose, J. M. Keller, J. R. Miller and K. S. Schanze, *J. Phys. Chem. B*, 2007, **111**, 10871–10880.

5 K. Onitsuka, A. Shimizu, F. Takei and S. Takahashi, *J. Inorg. Organomet. Polym. Mater.*, 2009, **19**, 98–103.

6 H. Nowell, S. A. Barnett, K. E. Christensen, S. J. Teat and D. R. Allan, *J. Synchrotron Radiat.*, 2012, **19**, 435–441.

7 S. A. Barnett, H. Nowell, M. R. Warren, A. Wilcox and D. R. Allan, *Protein Pept. Lett.*, 2015, **23**, 211–216.

8 D. R. Allan, S. P. Collins, G. Evans, D. Hall, K. McAuley, R. L. Owen, T. Sorensen, C. C. Tang, F. von Delft, A. Wagner and H. Wilhelm, *Eur. Phys. J. Plus*, 2015, **130**, 1–20.

9 CrysAlisPRO, *Oxford Diffr. /Agilent Technol. UK Ltd,* Yarnton, England.

10 G. M. Sheldrick, *SADABS,Empirical Absorpt. Correct. program, Univ. Göttingen*, 1995, based on the methods of Blessing.

11 L. Krause, R. Herbst-Irmer, G. M. Sheldrick and D. Stalke, *J. Appl. Crystallogr.*, 2015, **48**, 3–10.

12 R. H. Blessing, *Acta Crystallogr. Sect. A Found. Adv.*, 1995, **A51**, 33–38.

13 G. M. Sheldrick, *Acta Crystallogr. Sect. C Struct. Chem.*, 2015, **71**, 3–8.

14 O. V. Dolomanov, L. J. Bourhis, R. J. Gildea, J. A. K. Howard and H. Puschmann, *J. Appl. Crystallogr.*, 2009, **42**, 339–341.

15 I. H. M. Van Stokkum, D. S. Larsen and R. Van Grondelle, *Biochim. Biophys. Acta - Bioenerg.*, 2004, **1657**, 82–104.

16 J. J. Snellenburg, S. P. Laptenok, R. Seger, K. M. Mullen and I. H. M. van Stokkum, *J. Stat. Softw.*, 2012, **49**.

17 P. Dedecker, *J. Chemom.*, 2014, **28**, 137–138.
